# Resistance Training Interventions for Lower Limb Tendinopathies: A Scoping Review of Resistance Training Reporting Content, Quality, and Scientific Implementation

**DOI:** 10.1155/2022/2561142

**Published:** 2022-03-10

**Authors:** Ian Burton, Aisling McCormack

**Affiliations:** ^1^MSK Service, Fraserburgh Physiotherapy Department, Fraserburgh Hospital, NHS Grampian, Aberdeen, UK; ^2^Aberdeen City Council, Aberdeen, UK

## Abstract

The objectives of this scoping review were as follows: (1) to describe what exercises and intervention variables are used in resistance training interventions for lower limb tendinopathy,  (2) to assess the completeness of reporting as assessed by the Consensus on Exercise Reporting Template (CERT) and the Toigo and Boutellier framework, and (3) to assess the implementation of scientific resistance training principles. We searched MEDLINE, CINAHL, AMED, Embase, SPORTDiscus, and Cochrane Library databases. Randomized controlled trials, cohort studies, case series, case reports, and observational studies that reported using resistance exercises for lower limb tendinopathies were considered for inclusion, with 194 studies meeting the inclusion criteria. Completeness of the reporting of exercise descriptors and programme variables was assessed by the CERT and the Toigo and Boutellier framework. Reporting of exercise descriptor items from the Toigo and Boutellier framework ranged from 0 to 13, with an average score of 9/13, with only 9 studies achieving a full 13/13. Reporting of items from the CERT ranged from 0 to 18, with an average score of 13/19. No study achieved a full 19/19; however, 8 achieved 18/19. Scoring for resistance training principles ranged from 1 to 10, with only 14 studies achieving 10/10. Eccentric heel-drops were the most common exercise (75 studies), followed by isotonic heel raises (38), and single-leg eccentric decline squats (27). The reporting of exercise descriptors and intervention content was high across studies, with most allowing exercise replication, particularly for Achilles and patellar tendinopathy. However, reporting for some tendinopathies and content items such as adherence was poor, limiting optimal translation to clinical practice.

## 1. Introduction

Tendinopathy represents a spectrum of potential changes to healthy tendons, leading to tendon damage and disease, with changes characterised by abnormal tendon composition and cellularity, ultimately leading to altered tendon microstructure [1]. In tendinopathic tendons, the normal arrangement of collagen fibres and organisation of tenocytes become altered by mechanical overuse, which leads to tendinopathy symptoms of pain, inflammation, swelling, and impaired physical function and performance [2]. Despite all healthy tendons having the capability to progress to tendinopathy, tendons of the lower limb may have an increased risk for mechanical overload which causes tendon changes, such as disruption to the normal collagen matrix structure [3]. Although the aetiology of tendinopathy has yet to be fully elucidated, it is considered the result of a disrupted tendon healing process, with the hallmarks of collagen derangement, neovascularisation, altered tendon structure, and tissue calcification [4]. Tendinopathies account for up to 30% of all sports-related injuries with a range of extrinsic and intrinsic risk factors identified, suggesting that each individual pathogenesis of tendinopathy is multifactorial [5, 6]. The higher prevalence of patellar and Achilles tendinopathy found in athletes may be related to repetitive tendon microtrauma from repeated athletic movements such as running, jumping, and landing [7]. Prevalence of Achilles and patellar tendinopathy have been reported to be as high as 23% and 45% in runners and jumping athletes, respectively. Plantar heel pain was reported to be found in up to 18% of running athletes in one cohort [8–10].

Despite a recent proliferation in clinical research investigating effectiveness of a range of treatment options for tendinopathy, it remains unclear which treatments are most effective, with exercise-based treatments such as resistance training currently being the most recommended due to consistent findings of efficacy [11, 12]. Common adjunctive treatments to exercise in clinical practice, which have conflicting findings of effectiveness include shockwave therapy, ultrasound, low-level laser therapy, manual therapy, and corticosteroid injections [13]. Isolated eccentric resistance training and heavy slow resistance training involving isotonic contractions have been shown to have favourable outcomes for common lower limb tendinopathies including Achilles tendinopathy, patellar tendinopathy, and plantar heel pain [14–17]. The high loads encountered during resistance training may stimulate tendon healing by counteracting structural tendon alteration, leading to reorganization and remodelling of collagen fibres, therefore improving the mechanical properties of tendons [18]. Despite positive outcomes, a limitation of some resistance training interventions in tendinopathy studies is that description, prescription, and progression of exercises and program variables may be poorly defined and reported, making translation to clinical practice difficult [19]. If the exercise dosage and training parameters prescribed clinically is insufficient, then, the mechanobiological stimulus may not be adequate to initiate tendon healing and positive outcomes from the intervention [20]. Despite the optimal dosage of resistance training for tendinopathy being unknown [21], research has shown an association between positive outcomes and higher exercise dosages in other musculoskeletal disorders [22].

In recent years, several guidelines or frameworks have been developed for reporting exercise interventions and specific exercise details within research studies in order to enhance reproducibility of exercise interventions and their translation to clinical practice [23]. The need to standardise reporting of components of exercise interventions has been highlighted by the development of the Consensus on Exercise Reporting Template (CERT), which advocates reporting detailed descriptions of exercises and their variables such as progression and tailoring, to allow clinical replication [24]. However, a limitation of the CERT is its omission of mechanobiological resistance training descriptors such as those included in the Toigo and Boutellier framework, such as rest intervals, time under tension, and relative load [25]. Holden et al. [26] recently highlighted how the poor reporting of exercise interventions in patellofemoral pain limits the clinical translation of exercise research findings in this population, with the authors recommending that future studies should use both the CERT and Toigo and Boutellier framework in conjunction as they report different aspects of exercise prescription and would therefore be complementary. It is unclear if a similar issue exists within the interventional exercise literature in lower limb tendinopathies as no previous reviews have been conducted investigating the reporting of exercise descriptors using recommended frameworks. Although reporting of exercise interventions using the CERT has been recommended in tendinopathy to improve transparency and clinical translation, it is unclear if this recommendation has been widely adopted in research studies [27–29]. Both the CERT and Toigo and Boutellier framework are recommended templates and have been used in several review studies evaluating exercise descriptions and variables in rehabilitation for musculoskeletal disorders other than tendinopathy [30–32].

Although there has been a proliferation of clinical research examining resistance training treatment interventions for lower limb tendinopathies in recent years, it is unclear if these interventions have been sufficiently reported to allow clinical replication. Therefore, conducting a comprehensive scoping review of the current literature is warranted to investigate this question [29]. A search of MEDLINE, CINAHL, Cochrane Library, and PEDro identified no reviews with the objective of evaluating the reporting of resistance exercises and variables within interventions for lower limb tendinopathies using recommended frameworks such as the CERT or Toigo and Boutellier framework [33]. Therefore, the aims of this scoping review were to evaluate the reporting of exercise descriptors and programme variables used within resistance training interventions for treating lower limb tendinopathies. The scoping review was guided by addressing the following review questions on specific aspects of exercise reporting within lower limb tendinopathy resistance training interventions: (1) What exercises and program variables are used in resistance training interventions for lower limb tendinopathy? (2) How complete is the reporting of the exercise descriptors and programme variables as assessed by the CERT and the Toigo and Boutellier framework? (3) Do studies implement scientific resistance training principles?

## 2. Methods

Due to the exploratory nature of the research questions of this review, a scoping review was conducted as they are recommended for mapping key concepts, evidence gaps, and types of evidence within a particular field [34]. Scoping reviews can help guide future research and the possibility of conducting systematic reviews on the topic. As the aim of this review was to evaluate reporting across all study designs implementing resistance training for lower limb tendinopathies, rather than evaluate effectiveness, a scoping review was more appropriate than a systematic review. This scoping review was conducted in accordance with the Joanna Briggs Institute (JBI) methodology for scoping reviews [34]. The scoping review was reported in accordance with the Preferred Reporting Items for Systematic reviews and Meta-Analysis extension for Scoping Reviews (PRISMA-ScR) [34]. The findings allowed for dissemination of the parameters of research exercise interventions to clinical practitioners, allowing increased likelihood of implementation in clinical practice [26]. The review also outlined future research and exercise reporting needs within lower limb tendinopathy resistance training interventions.

### 2.1. Study Identification

The inclusion criteria for this scoping review were guided by a modified PICO, which includes population, concept, and context (PCoCo), as recommended for scoping reviews [34]. The review included adults aged eighteen years or older with a diagnosis of a lower limb tendinopathy for any time duration. All lower limb tendinopathies were included, such as gluteal, hamstring, patellar, Achilles, tibialis posterior, and peroneal tendinopathy. Plantar heel pain was included as it is considered to have a similar pathophysiology to tendinopathy and should therefore be treated in accordance with other lower limb tendinopathies according to recent literature [19]. The concept of interest was resistance training for the treatment of lower limb tendinopathies, including any type or format of resistance training. The resistance training may be used as a first- or second-line intervention and may be delivered in isolation or combined with other treatments. Resistance training may be delivered across any healthcare or exercise setting, delivered by health or exercise professionals, in a supervised or unsupervised manner, using any methods for training progression and monitoring. This scoping review considered both experimental and quasiexperimental study designs including randomized controlled trials and nonrandomized controlled trials. In addition, prospective and retrospective cohort studies, case series, and case reports were considered for inclusion. Unpublished studies, reviews, or reports were not considered.

### 2.2. Search Strategy

A 3-step search strategy was implemented in this scoping review. It incorporated the following: (1) A limited search of MEDLINE and CINAHL using initial keywords, followed by analysis of the text words in the title or abstract and those used to describe articles to develop a full search strategy. (2) The full-search strategy was adapted to each database and applied to MEDLINE, CINAHL, AMED, Embase, SPORTDiscus, Cochrane Library (controlled trials and systematic reviews), and PEDro. The following trial registries were searched: ClinicalTrials.gov, ISRCTN, The Research Registry, EU-CTR (European Union Clinical Trials Registry), and ANZCTR (Australia and New Zealand Clinical Trials Registry). Databases were searched from inception to December 31, 2021. Although Stanish and Curwin [35] first published on the concept of eccentric resistance training for tendinopathy in 1986, it was only following the publication of the Alfredson protocol in 1998 [36] that resistance training became widespread in lower limb tendinopathy rehabilitation. Despite this, databases were searched from inception to ensure key articles and seminal studies on the topic published before 1998 were not omitted, which may influence overall findings. (3) For each article located in steps 1 and 2, a search of cited and citing articles using Scopus and hand-searching, where necessary, was conducted. Studies published in a language other than English were only included if a translation was available as translation services were not available to the authors.

### 2.3. Study Selection

**Figure 1:**
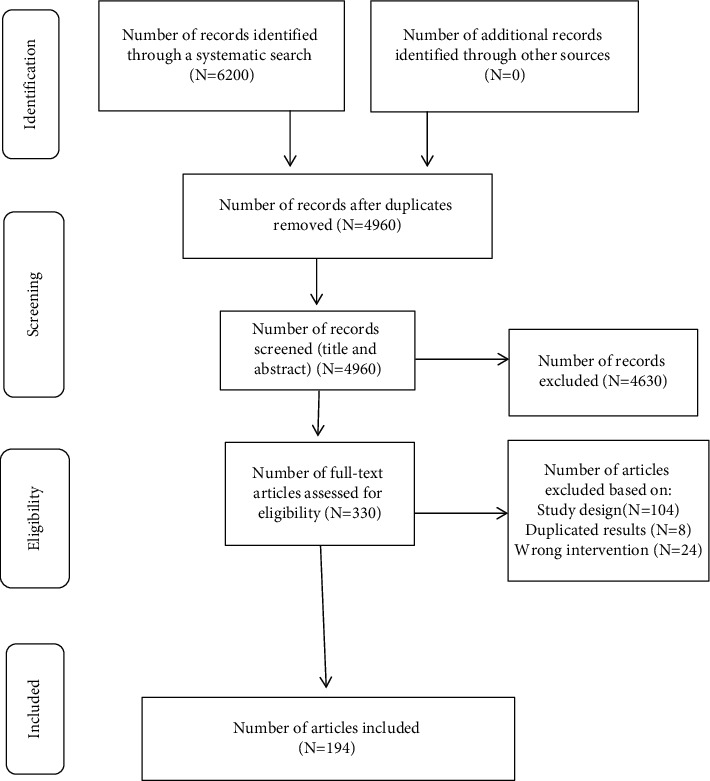
PRISMA study flow diagram.

Following the search, all identified citations were collated and uploaded into RefWorks, with duplicates being removed. Titles and abstracts were then screened by two independent reviewers (IB and AM) for assessment against the inclusion criteria for the review. Potentially relevant studies were retrieved in full, and their citation details imported into Covidence (Veritas Health Innovation, Melbourne, Australia). Two independent reviewers (IB and AM) then assessed the full text of selected citations in detail against the inclusion criteria. Any disagreements that arose between the reviewers at each stage of the study selection process were resolved through discussion or by input from a third reviewer. The results of the search were reported in accordance with the PRISMA-ScR (Figure 1) [34].

### 2.4. Data Extraction

Data were extracted from sources included in the scoping review by one reviewer, with independent data extraction by a second reviewer for 10% of studies. The data were extracted using an excel spreadsheet developed specifically by the reviewers for each source type. The data extracted included specific details regarding the population, concept, context, study methods, and key findings relevant to the review questions. Any disagreements that arose between the reviewers were resolved through discussion. The data extracted included dimensions such as authors, year of publication, study type, purpose, population and sample size, methods, details of resistance training intervention, specific exercises, and outcome measures used. Details of the resistance training interventions included setting, mode of delivery, type, dosage, and methods used to progress and adjust the training stimulus. The contents and variables of the specific resistance training exercises were extracted using the 13-item Toigo and Boutellier framework for exercise mechanobiological determinants and included parameters such as repetitions, load magnitude, and time under tension. General information from the resistance training interventions such as exercise supervision and delivery methods were extracted using the CERT tool. An evaluation of the implementation of scientific resistance training principles was also conducted, by extracting data on the principles of specificity, overload, progression, individualisation, and adherence. The definitions and scoring criteria for these principles are provided in Tables 1 and 2. In accordance with guidance on conducting scoping reviews, critical appraisal was not conducted [34].

### 2.5. Data Analysis

The extracted data were analysed using descriptive statistics, with findings presented in tabular form as tables and figures, in a manner that aligns with the objective of this scoping review. A narrative summary accompanies the tabulated results and describes how the results relate to the review objective and questions. Completeness of information regarding the resistance training interventions is presented as the number of complete items of the CERT, Toigo and Boutellier framework, and resistance training principles for each study. Resistance training exercises were categorised as eccentric, concentric, isometric, isotonic, heavy slow resistance training (HSRT), low-load blood flow restricted training (BFRT), isoinertial, manually resisted, hip strength, and general strength exercise.

## 3. Results

### 3.1. Study Characteristics

A total of 194 studies were included (Table 3), of which 109 were randomized controlled trials (RCTs), 32 were cohort studies, 15 were case series, 26 were case reports, with 12 being other designs, quasiexperimental (4), before-after design (2), case-control (4), and observational (2). The publication year ranged from 1989 to 2021, with 42 of the included studies being published since the year 2020, and 62 since 2019. The tendinopathy most frequently treated was Achilles (99), followed by patellar (58), plantar heel pain (16), gluteal (8), posterior tibial (6), hamstring (5), peroneal (1), extensor hallucis longus (1), and iliopsoas (1). The sample sizes of included studies ranged from 1 to 204 patients. The duration of included resistance training interventions ranged from a single session to 32 weeks, with 12 being the most common, implemented in 122 studies (63%). All studies evaluated intervention outcomes for at least one of pain and function, with the vast majority evaluating both outcomes. Pain was assessed by a visual analogue scale (VAS) in 90 (46%) studies, and pain numeric rating scale (NRS-P) in 21 (11%) studies. Pain and function were assessed by the Victorian Institute of Sport Assessment-Achilles (VISA-A) in 66 (34%) studies, Victorian Institute of Sport Assessment-Patellar (VISA-P) in 45 (23%) studies, Victorian Institute of Sport Assessment-Gluteal (VISA-G) in 5 (3%) studies, Foot Function Index (FFI) in 10 (5%) studies, and Lower Extremity Function Scale (LEFS) in 10 (5%) studies. Six (3%) studies also assessed the tendon structure through ultrasonography and four (2%) studies used quality of life outcome measures.

### 3.2. Content and Completeness of Exercise Description

Eccentric training was the most common type of resistance training, implemented in 130 (67%) studies, followed by general strength exercise in 35 (18%) studies, HSRT in 15 (8%) studies, isometric in 14 (7%) studies, isotonic in 11 (6%) studies, concentric in 6 (3%) studies, isoinertial in 4 (2%) studies, hip strength exercises in 4 (2%) studies, BFRT in 2 (1%) studies and manually resisted exercise in 1 (0.5%) study. In terms of specific resistance training exercises, the Alfredson eccentric heel-drop was the most common exercise with 75 (39%) studies implementing it, followed by isotonic heel raises in 38 (20%) studies, single-leg eccentric decline squats in 27 (14%) studies, knee extension in 16 (8%) studies, leg press in 13 (7%) studies, hip abduction in 10 (5%) studies, hip bridging in 9 (5%) studies, deadlifts in 8 (4%) studies, ankle inversion in 7 (4%) studies, plyometric jump exercises in 6 (3%) studies, and lunges in 5 (3%) studies.

**Figure 2:**
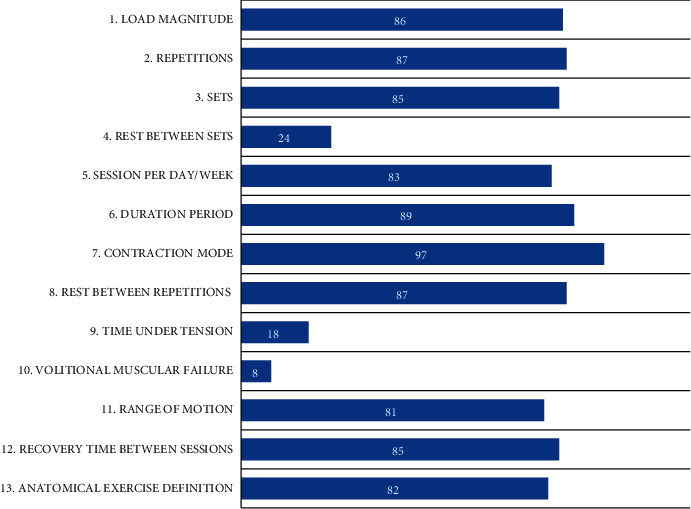
Percentage of studies (out of 194) with complete reporting for each item of the Toigo and Boutellier framework.

**Figure 3:**
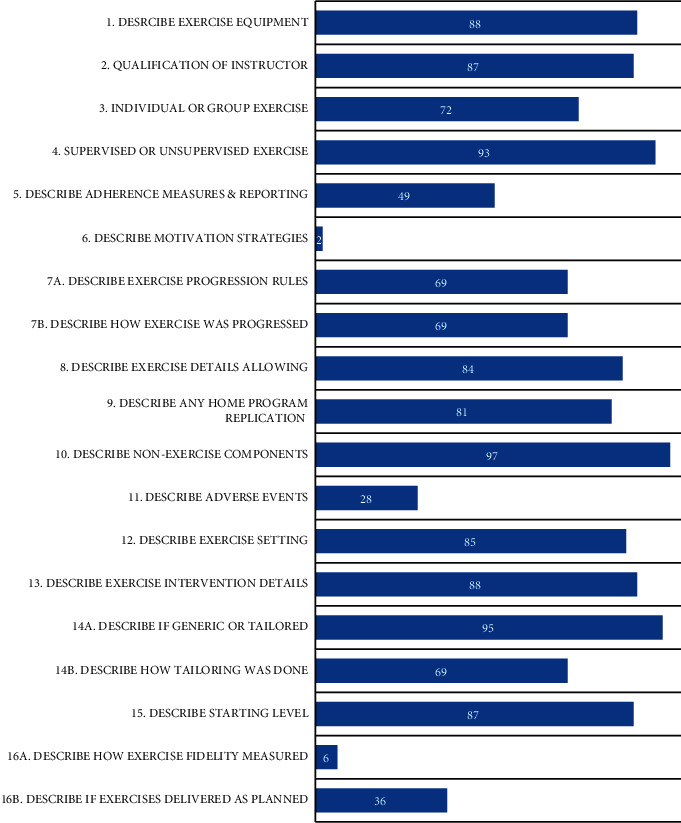
Percentage of studies (out of 194) with complete reporting for each item of the Consensus on Exercise Reporting Template (CERT).

The number of items that described the Toigo and Boutellier framework exercise descriptors ranged from 0 to 13 out of a possible 13, with an average score across the 194 studies of 9/13. Only 9 [19, 50, 79, 155, 175, 177, 201, 217, 221] (5%) studies achieved a full 13/13 for reporting items from the framework, with three of these from the same author [19, 217, 221]. Overall reporting across all studies for each item is reported in Figure 2. Only 3 items were reported by less than 80% of studies, rest between sets 47 (24%), time under tension 34 (18%), and volitional muscular failure 15 (8%). The item with the highest percentage of reporting at 97% was the contraction mode of the exercise employed in the intervention, reported in 188 studies. Of the 19 items included in the CERT, reporting among included studies ranged from 0 to 18, with an average score of 13/19. No study achieved a full score of 19, but 8 [15, 50, 79, 110, 111, 137, 177, 221] (4%) studies achieved a high score of 18/19. Four of these studies [50, 79, 177, 221] also achieved a full score of 13/13 for reporting exercise descriptors. Overall reporting for each item is presented in Figure 3. Most items were well-reported across studies, with seven items being reported in less than 70% of studies, progression rules 133 (69%), tailored how 134 (69%), adherence measures 94 (49%), exercise delivered as planned 70 (36%), adverse events 55 (28%), fidelity measured 11 (6%), and motivation strategies 4 (2%), with the latter two items particularly poorly reported across the studies. Previous studies assessing the completeness of CERT items in musculoskeletal rehabilitation determined that reporting completeness of items could be regarded as high (>75%), moderate (60 to 74%), or low (<60%) [225, 226]. Based on this classification, 10 items can be rated as high, 4 as moderate, and 5 as low.

### 3.3. Application of Resistance Training Principles

An evaluation of the implementation of scientific resistance training principles was conducted by evaluating the design and reporting of the key principles of specificity, overload, progression, individualisation, and adherence (Table 1). One point each was given for the design and reporting of each of the 5 principles, with a maximum score of 10/10 available. The scoring system was based on scales used in previous reviews with the same objective [11, 227, 228]. Scoring for resistance training principles ranged from 1 to 10 across the 194 studies, with only 14 studies achieving a full score of 10/10 [49, 60, 66, 67, 78, 90, 113, 137, 150, 151, 155, 179, 187, 200]. Only one study did not implement and report the principle of specificity, whereas 193 (99%) studies implemented specificity by targeting the prescribed resistance training to the specific tendinopathy with the aim to improve pain and function. The principle of overload was not adequately implemented or reported in 45 studies, with 149 (77%) studies implementing overload by progressively increasing training resistance throughout the intervention. The principle of progression was not adequately implemented or reported in 57 studies, with 137 (71%) studies implementing progression, most commonly by increasing resistance through small increases in external weight. However, only 35 (18%) studies accurately reported the exact amount of weight implemented in progression increments. Incremental increases in resistance ranged from 0.9 to 5 kg, with 5 kg being the most common, implemented in 27 (14%) studies. The principle of individualisation was not adequately implemented or reported in 59 studies, with 135 (70%) studies implementing individualisation, most commonly by adjusting training resistance based on pain response as implemented in 118 (61%) studies. Other reported methods for individually tailored training included exercise technique in 6 (3%) studies, as much volume as possible in 3 (2%) studies, increasing exercise difficulty in 8 (4%) studies, and level of fatigue in 7 (4%) studies, typically measured by using rating of perceived exertion (RPE) scales. The principle of adherence was not adequately implemented or reported in 68 studies, with 126 (65%) studies implementing adherence, most commonly by using an individual exercise diary as reported in 72 (37%) studies. However, only 46 (24%) studies reported the percentage of participants who achieved what each study authors determined to be an acceptable level of resistance training adherence, which ranged from 40 to 100%. Although the median value of adherence was reasonably high at 70%, it is concerning that only 24% of included studies reported adherence levels.

## 4. Discussion

The main findings from this scoping review are that the description and reporting of resistance training exercises and intervention parameters used in the rehabilitation of lower limb tendinopathies were generally well reported across studies. However, some reporting areas of weakness were identified, with items such as adherence, fidelity, and specific exercise progression parameters of interventions, being poorly reported. A broad range of resistance training types were implemented with eccentric training the most common at 67%, with Alfredson eccentric heel-drops (39%), isotonic heel raises (20%), and eccentric decline single-leg squats (14%) being the most implemented exercises. Most studies included sufficient information on exercise dosage (load, repetitions, sets, and frequency) to allow replication of the exercises in both research and clinical settings. However, not all studies provided sufficient detail to allow replication, suggesting there is room for improvement in future research, with items such as adherence and fidelity of interventions being poorly reported. Whereas the scientific resistance training principles of specificity and overload were well implemented and reported throughout the studies, the principals of progression, individualisation, and adherence had poorer reporting, preventing complete clinical replication of these principles in some studies. Despite these issues, the overall moderate to high quality of reporting was better for lower limb tendinopathies than for other musculoskeletal conditions as assessed in other reviews applying the CERT. For example, the quality of exercise content reporting has been found to be considerably lower in exercise rehabilitation interventions for hamstring strains [225], groin injury [226], Achilles tendon ruptures [30], rotator cuff disorders [32], knee osteoarthritis [229, 230], patellofemoral pain [26], knee injuries [31], fibromyalgia [231], juvenile arthritis [232], hand osteoarthritis [233], pelvic floor dysfunction [234, 235], low back pain [236, 237], ACL injury [238], and femoral-acetabular impingement [239].

The only other review reporting an overall moderate–high quality for CERT reporting like this review, was for hip osteoarthritis, which reported an average CERT score of 13/19 [240]. Item 8 of the CERT relates to describing exercises to a level that allows replication, which was met by 85% of studies included in this review. In comparison, reporting of this item was much lower in the reviews for hamstring strains [225] (43%), knee osteoarthritis [230] (26%), rotator cuff disorders [32] (29%), groin injuries [226] (15%), and Achilles tendon ruptures [30] (26%). These findings highlight the comparative quality of exercise reporting for lower limb tendinopathies. The reasons for exercise reporting quality being higher for lower limb tendinopathies compared to other musculoskeletal conditions are unclear but could be related to the fact that resistance training has been considered the gold standard first-line intervention which has been recommended for many years [11].

This review employed two common tools to describe resistance exercise implementation and reporting; the CERT which evaluates general information about the specific exercise intervention; and the Toigo and Boutellier framework which evaluates mechanobiological and exercise dosage descriptors, alongside an evaluation of five key scientific resistance training principles. The completeness of exercise reporting was high overall across the included studies, but the poor reporting of some items and key resistance training principles is concerning and limits the true translation of the findings regarding the resistance exercises to clinical practice. For example, the poor overall reporting on the specific loading employed during resistance exercise for progression, makes this principle difficult to replicate and translate to clinical practice. Although the Toigo and Boutellier framework is a well-accepted tool for reporting exercise descriptors in the literature, some of the items which were poorly reported in the included studies could theoretically be considered too detailed and impractical to properly implement both clinically and in research. The items, rest between sets, time under tension, and volitional muscular failure were all poorly reported; however, it could be argued these items are the least relevant for exercise replication. Despite this, their inclusion would allow for a complete replication of the resistance training interventions implemented in the studies, which may be a more optimal scenario for prescribing resistance exercise to patients to ensure complete translation of the protocols. As a tool that was developed in the sports science literature, it is not rehabilitation specific, so key aspects of resistance exercises in the rehabilitation setting are not accounted for such as the patients psychological state, level of pain, and tolerance to exercise. Similarly, while most items on the CERT were well reported, several items were very poorly reported such as motivational strategies, fidelity measures, adherence, adverse events, and if exercises were delivered as planned. Although the absence of these items does not prevent exercise replication, their inclusion would optimise replication in the clinical setting. It is concerning that only 2% of studies reported using motivational strategies, which may impact on exercise adherence, and overall reporting of adherence was lacking, despite many studies stating they employed adherence tracking measures such as exercise diaries. The very poor reporting of fidelity and adherence highlighted in this review, highlights the need for future studies to focus on practical implementation issues to ensure translation to clinical practice. If fidelity and adherence of the interventions are not monitored and reported, then, the quality of the exercise intervention reported in studies may be of less value. While the combination of both scales would allow exercise replication in a clinical setting, in isolation, they would likely not be sufficient. Therefore, the development of a more rehabilitation specific scale for implementing and reporting resistance training interventions should be explored in future research to optimise clinical translation of research resistance exercise interventions.

### 4.1. Clinical Implications

For many years, progressive resistance training has been considered the gold standard intervention for rehabilitating lower limb tendinopathies. Optimising rehabilitation outcomes for patients with lower limb tendinopathies requires implementing the most effective evidence-based resistance training interventions. However, interventions shown to be efficacious in high-quality research must also be replicable and translatable to the clinical setting. To achieve this, research interventions must follow scientific resistance training principles and include enough detail to be reproducible. Many of the studies included in this review which have shown good outcomes also score highly for replicability on the scales employed, particularly those using eccentric heel raises for Achilles tendinopathy and eccentric decline squats for patellar tendinopathy. These protocols should remain the gold standard first-line intervention for clinicians as they have not only been found to be effective in the individual studies but can also be fully replicated clinically. The supplementary material for this review provides all the extracted data and key prescription content from the interventions and can help to guide clinicians in clinical practice. Several studies [50, 79, 177, 221] scored highly across all the reporting tools employed, so the authors recommend these as a starting point for clinicians requiring fully reproducible resistance training programs for implementing in rehabilitation for lower limb tendinopathies.

### 4.2. Implications for Research

In comparison to reviews on other musculoskeletal pathologies in physical therapy, which have evaluated exercise reporting, this review has found that reporting of resistance exercise for lower limb tendinopathies is generally of high quality, despite shortcomings in areas such as adherence reporting. This is in stark contrast to reviews on other common musculoskeletal disorders where the reporting was considered poor overall. Indeed, the average scores found in this review for the CERT and Toigo and Boutellier framework are higher than for all the other pathologies previously listed. However, this review has highlighted potential weaknesses in the delivery of exercise, with poor reporting of adherence, fidelity, exercise motivational strategies, and specific progression parameters. The implementation of these items should be addressed in future research to optimise clinical translation and outcomes for resistance training interventions in lower limb tendinopathies. Until a rehabilitation specific exercise reporting scale is validated, and available, future studies should continue to design and implement resistance training protocols using scales such as the CERT and Toigo and Boutellier framework, to ensure they are clinically reproducible.

### 4.3. Strengths and Limitations

This scoping review has included a broad range of study designs from RCTs to individual case reports, with a broad range of interventions, so there is therefore vast heterogeneity in findings across all the studies, so findings should be interpreted with caution. However, determining effectiveness of interventions through meta-analysis techniques was not the objective of the review, with the aims focused on the description, reporting, and implementation of resistance training in interventions for lower limb tendinopathies. Only studies available in English language were included, which may introduce language bias. To provide a comprehensive analysis and state of the art review on reporting of resistance training interventions for lower limb tendinopathies, all primary study designs were considered. Despite all primary designs being included, this review did not consider review papers or clinical practice guidelines, which may have included detailed exercise reporting. Databases were searched from inception, and there was no limitation on sample size or intervention duration. Although many studies included were published before the publication of the CERT (2016) and Toigo and Boutellier framework (2006), there was no obvious reporting discrepancies from earlier to more recent studies, despite the culture of reporting becoming more widespread in recent years [23, 26]. Both scales are transparent and contain sufficient exercise details to allow 100% replication if fully followed, despite not being rehabilitation or tendinopathy specific. Most of the studies included in this review were for Achilles and patellar tendinopathies which also had the highest quality reporting, with other lower limb tendinopathies poorly represented and with comparatively poorer overall reporting quality. Therefore, the findings of this review cannot be generalised to all lower limb tendinopathies, with future research required to address the dearth of resistance training interventions for non-Achilles and patellar lower limb tendinopathies.

## 5. Conclusion

Resistance training interventions and specific exercises are generally well reported across all primary study designs for treating lower limb tendinopathies, particularly eccentric training for Achilles and patellar tendinopathy. However, certain reporting items and training principles related to the delivery of exercise were poorly reported and implemented, including adherence, fidelity, exercise motivational strategies, and specific progression parameters. While most studies provided sufficient details to allow clinical exercise replication, the weaknesses highlighted must be addressed in future research to allow resistance training interventions and exercises to be fully clinically reproducible to enhance rehabilitation outcomes.

## Figures and Tables

**Table 1:** Resistance training principles and training intervention criteria assessed.

Principle	Criteria for this review
Specificity: training and desired adaptations should be specific to the tendinopathy and relevant to desired outcomes	Appropriate population targeted and intervention designed to improve primary outcome
Progression: to allow for continuous adaptations, resistance or load must be increased providing a greater stress to the body	Training intervention was stated to be progressive with gradual increases in frequency, sets, repetitions, resistance, or loading throughout intervention
Overload: for the intervention to improve strength, greater than normal stress and training volume must occur above current training levels	Interventions included baseline strength testing or rationale that intervention was of sufficient intensity and volume relative to baseline capacity
Individualisation: training is tailored to the individual to allow for consideration of individual factors and training response	Training intervention considered methods to individually tailor exercises stimulus based on an individual's own factors or training response
Component of training	Description
Frequency	How many times per week or day
Intensity	Measurement methods: RM, %RM, RPE, pain level
Time	Duration of session
Sets	How many sets of each exercise
Repetitions	How many repetitions of each exercise or target number of repetitions
Exercise selection	Outline and description of specific exercises used in intervention
Adherence	Was adherence to the training intervention monitored and reported?

**Table 2:** Application and reporting of key training principles.

Principle/criterion	Description	Score	
Specificity	Design: have the authors designed the intervention to achieve desired outcomes? 1/10	Reporting: have the authors adequately described the intervention specificity? 1/10	2/10
Overload	Design: have the authors appropriately manipulated training variables to achieve desired outcomes? 1/10	Reporting: have the authors adequately described the intervention training variables? 1/10	2/10
Progression	Design: have the authors appropriately manipulated training variables to adequately progress the intervention? 1/10	Reporting: have the authors adequately described how intervention progression was achieved and assured? 1/10	2/10
Individualisation	Design: have the authors appropriately manipulated training variables to tailor the intervention adequately individually? 1/10	Reporting: have the authors adequately described how individually tailoring the intervention was achieved and assured? 1/10	2/10
Adherence	Design: have the authors appropriately designed and described methods for monitoring adherence? 1/10	Reporting: have the authors adequately reported individual adherence to training and training dose achieved? 1/10	2/10

**Table 3:** Characteristics and reporting scores of the 194 included studies.

Author	Study design	Tendinopathy	Resistance training type	Resistance training exercise	TBF/13	CERT/19	RTP/10
Abdelkader et al. [37]	RCT	Achilles	ECCT	Alfredson heel-drop	11	11	2
Alfredson et al. [36]	RCT	Achilles	ECCT	Alfredson heel-drop	10	14	7
Balius et al. [38]	RCT	Achilles	ECCT	Alfredson heel-drop	8	10	4
Bell et al. [39]	RCT	Achilles	ECCT	Alfredson heel-drop	7	14	6
Beyer et al. [14]	RCT	Achilles	HSRT, ECCT	Heel raises	12	17	9
Boesen et al. [40]	RCT	Achilles	ECCT	Alfredson heel-drop	10	15	8
Brown et al. [41]	RCT	Achilles	ECCT	Alfredson heel-drop	1	1	1
Chester et al. [42]	RCT	Achilles	ECCT	Alfredson heel-drop	10	15	7
Choudhary et al. [43]	RCT	Achilles	ECCT	NR	8	12	7
De jonge et al. [44]	RCT	Achilles	ECCT	Alfredson heel-drop	10	14	8
De jonge et al. [45]	RCT	Achilles	ECCT	Alfredson heel-drop	6	11	5
De Vos et al. [46]	RCT	Achilles	ECCT	Alfredson heel-drop	6	11	5
De vos et al. [47]	RCT	Achilles	ECCT	Alfredson heel-drop	10	16	9
Gatz et al. [48]	RCT	Achilles	ECCT, ECCT + ISOM	Alfredson heel-drop	10	15	8
Habets et al. [49]	RCT	Achilles	ECCT, CONCT-ECCT	Alfredson heel-drop, heel raises	10	16	10
Hasani et al. [50]	RCT	Achilles	ISOT	Heel raises	13	18	9
Herrington et al. [51]	RCT	Achilles	ECCT	Alfredson heel-drop	10	16	8
Horstmann et al. [52]	RCT	Achilles	ECCT	Alfredson heel-drop	11	15	7
Kearney et al. [53]	RCT	Achilles	ECCT	Alfredson heel-drop	10	15	7
Kedia et al. [54]	RCT	Achilles	ECCT	Alfredson heel-drop	10	15	8
Knobloch et al. [55]	RCT	Achilles	ECCT	Alfredson heel-drop	10	11	2
Koszalinski et al. [56]	RCT	Achilles	ECCT	Alfredson heel-drop	7	10	2
Mafi et al. [57]	RCT	Achilles	ECCT, CONCT	Alfredson heel-drop	10	15	7
Mansur et al. [58]	RCT	Achilles	ECCT	Alfredson heel-drop	10	12	4
McCormack et al. [59]	RCT	Achilles	ECCT	Alfredson heel-drop	10	15	5
Munteanu et al. [60]	RCT	Achilles	ECCT	Alfredson heel-drop	10	16	10
Niesen-Vertommen [61]	RCT	Achilles	ECCT, CONCT	Heel raises	10	17	9
Norregaard et al. [62]	RCT	Achilles	ECCT	Alfredson heel-drop	10	15	9
Notarnicola et al. [63]	RCT	Achilles	ECCT	NR	3	3	2
Pearson et al. [64]	RCT	Achilles	ECCT	Alfredson heel-drop	1	5	7
Petersen et al. [65]	RCT	Achilles	ECCT	Alfredson heel-drop	10	16	8
Praet et al. [66]	RCT	Achilles	ECCT	Alfredson heel-drop	10	17	10
Rabusin et al. [67]	RCT	Achilles	ECCT	Alfredson heel-drop	10	17	10
Romero-morales [68]	RCT	Achilles	ECCT	Alfredson heel-drop	10	15	8
Romero-morales [69]	RCT	Achilles	ECCT	Alfredson heel-drop	10	15	8
Rompe et al. [70]	RCT	Achilles	ECCT	Alfredson heel-drop	11	17	9
Rompe et al. [71]	RCT	Achilles	ECCT	Alfredson heel-drop	11	17	8
Rompe et al. [72]	RCT	Achilles	ECCT	Alfredson heel-drop	10	16	8
Roos et al. [73]	RCT	Achilles	ECCT	Alfredson heel-drop	10	16	9
Silbernagel et al. [74]	RCT	Achilles	General strength EX	Heel raises, plyometric heel raises	10	16	8
Silbernagel et al. [75]	RCT	Achilles	ECCT	Heel raises, plyometric heel raises	10	15	8
Solomons et al. [76]	RCT	Achilles	General strength EX	NR	1	11	6
Stefansson et al. [77]	RCT	Achilles	ECCT	Alfredson heel-drop	10	14	8
Stergioulas et al. [78]	RCT	Achilles	ECCT	Heel raises	11	16	10
Stevens and Tan [79]	RCT	Achilles	ECCT	Alfredson heel-drop	13	18	9
Tumilty et al. [80]	RCT	Achilles	ECCT	Alfredson heel-drop	10	17	9
Tumilty et al. [81]	RCT	Achilles	ECCT	Alfredson heel-drop	10	17	9
Van der vlist et al. [82]	RCT	Achilles	ECCT	Heel raises, plyometric heel raises	12	17	9
Yelland et al. [83]	RCT	Achilles	ECCT	Alfredson heel-drop	10	17	8
Yu et al. [84]	RCT	Achilles	ECCT, CONCT	Heel raises, alfredson heel-drop	10	15	8
Zhang et al. [85]	RCT	Achilles	ECCT	Alfredson heel-drop	10	14	8
Kanniappan et al. [86]	Quasi	Achilles	ECCT, ISOM	Alfredson heel-drop, static plantar flexion	11	11	4
Stasinopoulos et al. [89]	Quasi	Achilles	ECCT	Alfredson heel-drop, heel raises	11	17	8
Van der vlist et al. [88]	Quasi	Achilles	ISOM, ISOT	Heel raises	12	17	8
De vos et al. [89]	OBS	Achilles	ECCT	Alfredson heel-drop	11	14	8
Masood et al. [90]	L-CC	Achilles	ECCT	Alfredson heel-drop	10	16	10
Abate et al. [91]	Cohort	Achilles	ECCT	Alfredson heel-drop	10	16	9
Alfredson et al. [95]	Cohort	Achilles	ECCT	Alfredson heel-drop	10	14	8
Alfredson et al. [96]	Cohort	Achilles	ECCT	Alfredson heel-drop	10	14	7
Angermann et al. [94]	Cohort	Achilles	General strength EX	Heel raises	10	15	9
De jonge et al. [95]	Cohort	Achilles	ECCT	Alfredson heel-drop	3	2	2
Fahlstrom et al. [96]	Cohort	Achilles	ECCT	Alfredson heel-drop	10	14	7
Jonsson et al. [97]	Cohort	Achilles	ECCT	Alfredson heel-drop	10	14	8
Lagas et al. [98]	Cohort	Achilles	ECCT	NR	1	0	2
Maffulli et al. [103]	Cohort	Achilles	ECCT	Alfredson heel-drop	11	16	9
Mansur et al. [104]	Cohort	Achilles	ECCT	Alfredson heel-drop	10	14	8
Mantovani et al. [110]	Cohort	Achilles	ISOM	Heel raises	12	18	8
O'Neill et al. [102]	Cohort	Achilles	ISOM	Dynamometer heel raise	10	11	4
Ohberg et al. [103]	Cohort	Achilles	ECCT	Alfredson heel-drop	10	14	7
Ooi et al. [104]	Cohort	Achilles	ECCT	Alfredson heel-drop	1	2	2
Robinson et al. [110]	Cohort	Achilles	ECCT	Heel raises	8	12	2
Sancho et al. [111]	Cohort	Achilles	General strength EX	Heel raises, hip abduction and extension, hops, jumps	10	18	9
Sayana et al. [112]	Cohort	Achilles	ECCT	Alfredson heel-drop	11	16	9
Shalabi et al. [113]	Cohort	Achilles	ECCT	Alfredson heel-drop	10	16	8
Von Wehren et al. [109]	Cohort	Achilles	ECCT	Alfredson heel-drop	10	11	4
Wei et al. [110]	Cohort	Achilles	ECCT	Alfredson heel-drop	10	15	9
Wetke et al. [111]	Cohort	Achilles	ISOT	Heel raises	10	16	9
Wheeler et al. [112]	Cohort	Achilles	ECCT	NR	1	3	2
Langberg et al. [113]	CC	Achilles	ECCT	Alfredson heel-drop	10	15	10
Park et al. [114]	CC	Achilles	ECCT	Alfredson heel-drop	11	14	8
Ram et al. [115]	CC	Achilles	ECCT	Alfredson heel-drop	10	13	8
Benito et al. [116]	C. series	Achilles	ECCT	Alfredson heel-drop	10	13	6
Deans et al. [117]	C. series	Achilles	General strength EX	NR	1	4	2
Jayaseelan et al. [118]	C. series	Achilles	ECCT	Alfredson heel-drop	10	12	3
Pavone et al. [119]	C. series	Achilles	ECCT	NR	1	4	2
Silbernagel et al. [120]	C. series	Achilles	ECCT, plyometric EX	Heel raises, plyometric heel raises	10	14	7
Syverston et al. [121]	C. series	Achilles	ECCT	Alfredson heel-drop	10	11	4
Wheeler et al. [122]	C. series	Achilles	General strength EX	NR	1	5	2
Borda et al. [123]	C. report	Achilles	ECCT	Alfredson heel-drop	10	11	2
Cuddeford et al. [124]	C. report	Achilles	ECCT	Heel-drop on leg press	8	15	8
Eckenrode et al. [125]	C. report	Achilles	ECCT	Alfredson heel-drop, bridging, hip abduction, squat	12	14	7
Francis et al. [126]	C. report	Achilles	ECCT	Alfredson heel-drop	10	15	8
Greene et al. [127]	C. report	Achilles	General strength EX	Heel raises, squats, leg pulls	8	14	7
McCormack et al. [128]	C. report	Achilles	ECCT	Alfredson heel-drop	10	14	7
Papa et al. [129]	C. report	Achilles	ECCT	Alfredson heel-drop	10	13	5
Ross et al. [130]	C. report	Achilles	General strength EX	Heel raises, plantarflexion, dorsiflexion, plyometric	10	16	8
Thompson et al. [131]	C. report	Achilles	General strength EX	Squats, pelvic thrust, deadlift, heel raises, band walks, lunges	11	14	7
Gardin et al. [132]	BAD	Achilles	ECCT	Alfredson heel-drop	10	10	4
Croisier et al. [133]	Cohort	Achilles, patellar	Isokinetic ECCT	Dynamometer heel raise, knee extension	10	16	8
Pinkelman et al. [134]	C. report	EHL	Manually resisted EX	Great toe extension	8	14	7
Clifford et al. [15]	RCT	Gluteal	ISOM, ISOT	Isometric and isotonic hip abduction exercises	12	18	9
Cowan et al. [135]	RCT	Gluteal	General strength EX	Isometric and isotonic hip exercises	10	17	9
Ganderton et al. [136]	RCT	Gluteal	General strength EX	Isometric and isotonic hip exercises	10	17	9
Mellor et al. [137]	RCT	Gluteal	General strength EX	Isometric and isotonic hip exercises	11	18	10
Ramon et al. [138]	RCT	Gluteal	General strength EX	Bridging, hip abduction and extension	10	12	2
Thompson et al. [139]	RCT	Gluteal	ECCT	Lunges, squats	6	10	5
Wheeler et al. [140]	RCT	Gluteal	General strength EX	Hip abduction, bridging, clams	7	13	7
Van Rooy et al. [141]	C. report	Gluteal	ECCT	Hip abduction, lunges bridging	10	12	4
Cacchio et al. [142]	RCT	Hamstring	General strength EX	Leg curls, lunge, squat, CM jumps, deadlift, hip strength exercises	8	7	4
Cushman et al. [143]	C. report	Hamstring	ECCT	Hip extension	10	12	4
Jayaseelan et al. [144]	C. report	Hamstring	General strength EX	Leg curl, deadlift, bridging, hip abduction	10	14	7
Krueger et al. [145]	C. report	Hamstring	HSRT	Squat, deadlift, hip thrust, leg curl, reverse lunge	11	15	8
McCormack et al. [146]	C. report	Hamstring	ECCT	Leg curl, hip extension, bridging, lunges Nordics, deadlift	10	13	4
Rauseo et al. [147]	C. report	Iliopsoas	ECCT	Hip flexion, bridging, squats, deadlift	10	15	8
Alvarez et al. [148]	RCT	P. tibial	General strength EX	Heel raises, plantarflexion, adduction, inversion	10	17	9
Houck et al. [149]	RCT	P. tibial	General strength EX	Heel raises, plantarflexion, adduction, inversion	11	17	9
Kulig et al. [150]	RCT	P. tibial	Isokinetic ECCT, CONCT	Resisted adduction with plantarflexion	12	17	10
Kulig et al. [151]	C. series	P. tibial	ECCT	Resisted adduction with plantarflexion	11	15	10
Robinson et al. [152]	C. series	P. tibial	General strength EX	Heel raises, short foot	8	11	2
Patla et al. [153]	C. report	P. tibial	General strength EX	Heel raises, pronation and supination	8	13	3
Abat et al. [154]	RCT	Patellar	ECCT	DSL squat	9	8	2
Agergaard et al. [155]	RCT	Patellar	HSRT, M-HSRT	Leg press and extension	13	17	10
Bahr et al. [156]	RCT	Patellar	ECCT	DSL squat	11	14	8
Biernat et al. [157]	RCT	Patellar	ECCT	DSL squat	10	14	7
Breda et al. [158]	RCT	Patellar	HSRT, ECCT	DSL squat, leg press, knee extension, hip strength exercises	10	17	9
Cannell et al. [159]	RCT	Patellar	ECCT, ISOT	Drop squat, knee extension and curl	11	14	8
Da Cunha et al. [160]	RCT	Patellar	ECCT	DSL squat	10	14	8
Dimitrios et al. [161]	RCT	Patellar	ECCT	DSL squat	11	17	8
Dragoo et al. [162]	RCT	Patellar	ECCT	NR	1	5	2
Frohm et al. [163]	RCT	Patellar	ECCT	DSL squat	11	14	8
Holden et al. [164]	RCT	Patellar	ISOM, dynamic EX	Knee extension	12	13	5
Jensen et al. [165]	RCT	Patellar	Isokinetic ECCT	Dynamometer heel raise	11	16	8
Jonsson et al. [166]	RCT	Patellar	ECCT, CONCT	DSL squat	10	15	7
Kaux et al. [167]	RCT	Patellar	ECCT	Wall squat	11	13	5
Kongsgaard et al. [16]	RCT	Patellar	HSRT, ECCT	DSL squat, hack squat, leg press, squat	12	17	9
Lee et al. [168]	RCT	Patellar	ECCT	DSL squat	11	14	9
Lopez-royo et al. [169]	RCT	Patellar	ECCT	DSL squat	10	14	7
MacDonald et al. [170]	RCT	Patellar	ECCT, ECCT + hip	DSL squat, isotonic hip exercises	10	16	8
Olesen et al. [171]	RCT	Patellar	HSRT	Squat, leg press, knee extension, hack squat	10	14	7
Pietrosimone et al. [172]	RCT	Patellar	ISOM	Knee extension	12	12	4
Rio et al. [173]	RCT	Patellar	ISOM, ISOT	Knee extension	12	13	5
Rio et al. [174]	RCT	Patellar	ISOM, ISOT	Knee extension	12	16	9
Ruffino et al. [175]	RCT	Patellar	HSRT, isoinertial	Squat, leg press, knee extension, hack squat	13	17	9
Scott et al. [176]	RCT	Patellar	HSRT	NR	1	5	2
Sprague et al. [177]	RCT	Patellar	HSRT	Squat, leg press, knee extension, hack squat	13	18	9
Stasinopolous et al. [178]	RCT	Patellar	ECCT	DSL squat	10	14	7
Steunebrink et al. [179]	RCT	Patellar	ECCT	Alfredson heel-drop	10	15	10
Thijs et al. [180]	RCT	Patellar	ECCT	DSL squat	10	16	7
Van Ark et al. [181]	RCT	Patellar	ISOT, ISOM	Knee extension	12	16	8
Van Ark et al. [182]	RCT	Patellar	ISOM, ISOT	Knee extension	12	14	8
Van der Worp et al. [183]	RCT	Patellar	ECCT	DSL squat	9	16	8
Visnes et al. [184]	RCT	Patellar	ECCT	DSL squat	10	15	9
Wang et al. [185]	RCT	Patellar	ECCT	Quadriceps and hamstring strengthening	1	3	2
Warden et al. [186]	RCT	Patellar	ECCT	DSL squat	10	17	9
Young et al. [187]	RCT	Patellar	ECCT	DSL squat	10	16	10
Vander doelen et al. [188]	R chart	Patellar	General strength EX	Knee extension, leg press, squat, hack squat	11	14	5
Purdam et al. [189]	Quasi	Patellar	ECCT	DSL squat, squat	10	14	7
Abat et al. [190]	Cohort	Patellar	Isoinertial ECCT	NR	10	12	5
Abat et al. [191]	Cohort	Patellar	Isoinertial ECCT	Leg press	10	10	4
Basas et al. [192]	Cohort	Patellar	ISOT	NR	8	14	7
Kaux et al. [193]	Cohort	Patellar	ECCT	NR	3	4	2
Kaux et al. [194]	Cohort	Patellar	ECCT, ISOM	DSL squat	11	16	9
Kongsgaard et al. [195]	Cohort	Patellar	HSRT	Knee extension, leg press, hack squat, squat	12	15	7
Panni et al. [196]	Cohort	Patellar	General strength EX	NR	1	2	2
Bianco et al. [197]	C. series	Patellar	General strength EX	DSL squat, squat, drop squat, SL squat, jump downs, mini squat	8	14	5
Morton et al. [198]	C. series	Patellar	ECCT	DSL squat	1	5	2
Munoz fernandez [199]	C. series	Patellar	General strength EX	Clams, bridging, DSL squat, squat, deadlift, short foot, hip abduction, pelvic drops	10	12	4
Romero-rodriguez et al. [200]	C. series	Patellar	Isoinertial ECCT	Flywheel	12	15	10
Skovlund et al. [201]	C. series	Patellar	LL-BFRT	Knee extension, leg press	13	17	9
Van ark et al. [202]	C. series	Patellar	General strength EX	Heel raises, squats, hip abduction, SL squat, lunges, step-downs, bridging, jumps	11	16	8
Cuddeford et al. [203]	C. report	Patellar	LL-BFRT	DSL squat, leg press	12	15	8
Dumont et al. [204]	C. report	Patellar	ECCT	Drop squats	10	16	9
Goldman et al. [205]	C. report	Patellar	ECCT	DSL squats, leg press, knee extension, leg curls, step-downs, heel taps	11	14	7
McCreesh et al. [206]	C. report	Patellar	ECCT	DSL squat	10	13	5
Rowan et al. [207]	C. report	Patellar	ECCT	DSL squats	8	10	2
Silva et al. [208]	C. report	Patellar	Hip strength EX	Hip extension, birddog, deadlift, drop jumps	11	14	8
Morgan et al. [209]	BAD	Patellar	General strength EX	NR	4	6	6
Hensley et al. [210]	C. report	Peroneal	General strength EX	Heel raises, inversion and eversion	10	13	5
Chesterton et al. [211]	RCT	Plantar	General strength EX	Foot, calf and hip strength exercises	2	14	6
Cil et al. [212]	RCT	Plantar	General strength EX	Foot, ankle and hip exercises	9	10	5
Johannsen et al. [213]	RCT	Plantar	General strength EX	Heel raises, inversion	4	8	4
Johannsen et al. [214]	RCT	Plantar	HSRT	Heel raises, inversion	3	5	2
Kamonseki et al. [215]	RCT	Plantar	Foot, hip strength EX	Foot, ankle and hip exercises	10	13	5
Rasenberg et al. [216]	RCT	Plantar	General strength EX	NR	1	0	3
Rathleff et al. [17]	RCT	Plantar	HSRT	Heel raises	11	14	5
Riel et al. [19]	RCT	Plantar	HSRT	Heel raises	13	17	9
Riel et al. [217]	RCT	Plantar	ISOM, ISOT	Heel raises	13	14	7
Ryan et al. [218]	RCT	Plantar	General strength EX	Inversion and eversion	6	11	3
Thong-On et al. [219]	RCT	Plantar	General strength EX	Heel raises, inversion and eversion, toe curls	10	17	9
Wheeler et al. [220]	RCT	Plantar	General strength EX	Heel raises, foot strength exercises	0	8	2
Riel et al. [221]	Cohort	Plantar	HSRT	Heel raise	13	18	9
Wheeler et al. [222]	Cohort	Plantar	General strength EX	Heel raises, IFM strength	2	5	2
Dos Santos et al. [223]	C. report	Plantar	Hip strength EX	Hip abduction, extension, adduction and flexion	5	9	2
Lee et al. [224]	C. report	Plantar	Hip strength EX	NR	1	4	2

CERT: Consensus on Exercise Reporting Template; TBF: Toigo and Boutellier Framework; RTP: resistance training principles; ECCT: eccentric training; CONCT: concentric training; ISOM: isometric: ISOT: isotonic; EX: exercise; LL-BFRT: low-load blood flow restriction training; HSRT: heavy slow resistance training; C. series: case series; C. report: case report; CC: case control; BAD: before-after design; L-CC: longitudinal case-control; OBS: observational; QUASI: quasiexperimental; P.tibial: Posterior tibial; NR: not reported; DSL: decline single-leg; R chart: retrospective chart review; FHL: flexor hallucis longus.

## Data Availability

All data relevant to the study are included in the article or are available in the supplementary files or appendices.

## Supplementary Materials

Supplementary Materials S1: MEDLINE search strategy for the review. S2: study characteristics and reporting scores of included studies. S3: application of resistance training principles in included studies. S4: Toigo and Boutellier framework exercise descriptors reporting for each study. S5: Consensus on Exercise Reporting Template (CERT) items reporting for each study.
