# Supplementary material for: Resistance Training Interventions for Lower Limb Tendinopathies: A Scoping Review of Resistance Training Reporting Content, Quality, and Scientific Implementation
Source: Transl Sports Med. 2022 Mar 10;2022:2561142. doi: 10.1155/2022/2561142 (PMC11023730; doi:10.1155/2022/2561142)
Supplement: Supplementary Materials — S1: MEDLINE search strategy for the review. S2: study characteristics and reporting scores of included studies. S3: application of resistance training principles in included studies. S4: Toigo and Boutellier framework exercise descriptors reporting for each study. S5: Consensus on Exercise Reporting Template (CERT) items reporting for each study. [file 2561142.f1.docx]

**SUPPLEMENTARY MATERIAL (APPENDICES)**

**APPENDIX 1: MEDLINE search strategy:**

1. MH tendinopathy OR MH fasciitis, plantar KW tendin* OR KW tendon* OR KW tendinopath* OR KW plantar OR KW Achilles OR KW Patellar OR KW Gluteal OR KW Greater trochanter*)

2. MH resistance training OR MH exercise OR MH physical therapy modalities OR MH physical therapy specialty OR KW physiotherapy OR KW physical therapy OR KW exercis* OR KW strength training OR KW training

3. 1 AND 2

KW: Keyword, MH: MeSH heading

Dates inception-December 31^st^ 2021

Planned limits: English language only

**APPENDIX 2: Table 4: Study characteristics & reporting scores**

| Author | Tendinopathy | Intervention groups | Sample size | Intervention duration (wks) | Outcomes + measures | Follow-up length (weeks) | Outcomes/ results | TBF /13 | CERT /19 |
| --- | --- | --- | --- | --- | --- | --- | --- | --- | --- |
| RCT |  |  |  |  |  |  |  |  |  |
| Beyer et al. 2015 | Achilles | 1. HSRT  2. ECCT | 58 | 12 | Pain (VAS), Function (VISA-A), Ultrasound | 52 | Both interventions were effective, with HSRT having greater patient satisfaction at 12 but not 52 weeks. | 12 | 17 |
| Kongsgaard et al. 2009 | Patellar | 1. CSI 2. HSRT 3. ECCT | 37 | 12 | Pain (VAS), Function (VISA-P), Ultrasound | 26 | All groups improved, with only exercise groups maintaining improvements at 6 months. HSRT has good short- and long-term clinical effects. | 12 | 17 |
| Riel et al. 2019 | Plantar heel | 1. fixed HSRT 2. Self-dosed HSRT | 70 | 12 | Function (FHSQ), Pain (self-efficacy), ultrasound | 12 | Both groups improved pain and function, with no significant differences between groups. | 13 | 17 |
| Stevens & Tan 2014 | Achilles | 1. fixed ECCT 2. Self-dosed ECCT | 28 | 6 | Pain (VAS), Function (VISA-A) | 6 | Both groups improved pain and function, with no significant differences between groups. | 13 | 18 |
| Da Cunha et al. 2012 | Patellar | 1. ECCT pain 2. ECCT no pain | 17 | 12 | Pain (VAS), Function (VISA-P) | 12 | No difference between groups, both groups improved pain and function. | 10 | 14 |
| Kulig et al. 2009 | Posterior tibial | 1. ECCT 2. CONCT 3. Orthoses | 36 | 12 | Pain (VAS), function (FFI) | 12 | Eccentric program was more effective than concentric or orthoses alone. | 12 | 17 |
| Bahr et al. 2006 | Patellar | 1. ECCT 2. surgery | 35 | 12 | Pain, function (VISA-P) | 12 | Both groups improved, no significant difference between groups. Trend favouring ECCT. | 11 | 14 |
| Lee et al. 2020 | Patellar | 1. ECCT 2. ECCT + ESWT | 34 | 12 | Pain (VAS), function (VISA-P), ultrasound | 12 | Combining exercise and ESWT could not been shown to be more effective than exercise alone | 11 | 14 |
| Frohm et al. 2007 | Patellar | 1. Standard ECCT 2. Overload ECCT | 20 | 12 | Pain (VAS), function (VISA-P) | 12 | Both treatment groups improved in the short term, with no significant difference between groups. | 11 | 14 |
| Silbernagel et al. 2001 | Achilles | 1. Overload ECCT 2. control | 40 | 12 | Pain (VAS), function, task performance | 52 | No significant difference between groups, at 1-year ECCT group more satisfied with outcomes. | 10 | 15 |
| Balius et al. 2016 | Achilles | 1. ECCT 2. ECCT + supplement 3. Supplement + stretching | 59 | 12 | Pain (VAS), function (VISA-A), ultrasound | 12 | Reduction in pain at rest was greater in the groups who took the supplement than in the ECCT alone group | 8 | 10 |
| Mafi et al. 2001 | Achilles | 1. ECCT 2. CONCT | 44 | 12 | Pain (VAS), function | 12 | The results after treatment with eccentric training was significantly better (P<0.002) than after concentric training. | 10 | 15 |
| Norregaard et al. 2007 | Achilles | 1. ECCT 2. Stretching | 45 | 12 | Manually tested Pain, function | 52 | Marked improvement in symptoms and findings could be gradually observed in both groups during the 1-year follow-up period. | 10 | 15 |
| Stasinopolous et al. 2004 | Patellar | 1. ECCT 2. Ultrasound 3. MT | 30 | 4 | Pain | 4 | ECCT was statistically significantly better than the other two treatments at the end of treatment. | 10 | 14 |
| De Vos et al. 2007 | Achilles | 1. ECCT 2. ECCT +night splint | 70 | 12 | Pain, function (VISA-A) | 12 | Both groups improved pain and function, with no significant difference between groups | 10 | 16 |
| Johannsen et al. 2019 | Plantar Heel | 1. HSRT 2. CSI 3. HSRT + CSI | 90 | 12 | Pain (VAS), function (FFI), ultrasound | 26 | Combined treatment is superior both in the short- and in the long-term. | 3 | 5 |
| MacDonald et al. 2019 | Patellar | 1. ECCT 2. ECCT + hip exercises | 41 | 12 | Pain, function (VISA-P, LEFS) | 24 | Favourable effects were demonstrated with combined treatment of eccentric squat and hip muscle strengthening or squat only | 10 | 16 |
| Gatz et al. 2020 | Achilles | 1. ECCT 2. ECCT + isometric | 42 | 12 | Pain, function (VISA-A), shear wave elastography | 12 | Isometric exercises do not have additional benefit when combined with eccentric exercises, as assessed over a 3-month intervention period. | 10 | 15 |
| Ganderton et al. 2018 | Gluteal | 1. Ex 2. Sham Ex | 94 | 12 | Pain, function (VISA-G) | 52 | Lack of treatment effect was found with the addition of an exercise program to education | 10 | 17 |
| Silbernagel et al. 2007 | Achilles | 1. Rehab with continued sports 2. Control | 38 | 12 | Pain (VAS), function (VISA-A) | 26 | Significant improvement and no negative effects demonstrated from continuing Achilles tendon-loading activity, such as running and jumping, with the use of a pain-monitoring model, during treatment. | 10 | 16 |
| Clifford et al. 2019 | Gluteal | 1. isometric Ex 2. Isotonic Ex | 30 | 12 | Pain (NRS), function (VISA-G), QoL | 12 | Both groups effective in reducing pain and improving function, no difference between groups. | 12 | 18 |
| Stergioulas et al. 2008 | Achilles | 1. ECCT + LLLT 2. ECCT | 52 | 8 | Pain (VAS), function (VISA-A) | 12 | LLLT accelerates clinical recovery when added to ECCT | 11 | 16 |
| Rompe et al. 2008 | Achilles | 1. ECCT 2. ESWT | 50 | 12 | Pain, function (VISA-A) | 16 | ESWT superior to ECCT at 16 weeks. | 11 | 17 |
| Mellor et al. 2018 | Gluteal | 1. Ex, education 2. CSI 3. control | 204 | 8 | Pain (NRS), function (VISA-G), QoL (EQ5D), GROC | 52 | At 52-week follow-up, education plus exercise led to better global improvement than corticosteroid injection use, but no difference in pain intensity | 11 | 18 |
| Van Ark et al. 2016 | Patellar | 1. isotonic Ex 2. Isometric Ex | 29 | 4 | Pain (NRS), function (SLDS) | 4 | Both isometric and isotonic exercise programs improved pain and function | 12 | 16 |
| Roos et al. 2004 | Achilles | 1. ECCT 2. ECCT + night splint 3. Night splint | 44 | 6 | Pain, function (FAOS) | 52 | ECCT more effective than night splint for improving pain and function | 10 | 16 |
| Chester et al. 2008 | Achilles | 1. ECCT 2. Ultrasound | 16 | 12 | Pain (VAS), function (FILLA), QoL (EQ5D) | 12 | There were no significant differences between groups or clear trends over time. Both interventions proved acceptable with no adverse effects. | 10 | 15 |
| Rompe et al. 2007 | Achilles | 1. ECCT 2. ESWT 3. Control | 75 | 12 | Pain, function (VISA-A) | 16 | ECCT and ESWT showed comparable positive results. The wait-and-see strategy was ineffective. | 10 | 16 |
| Thijs et al. 2017 | Patellar | 1. ECCT + ESWT 2. ECCT | 52 | 12 | Pain, function (VISA-P) | 12 | No additional effect of ESWT to EECT for pain and function improvement. | 10 | 16 |
| Horstmann et al. 2013 | Achilles | 1. ECCT 2. Vibration training 3. control | 58 | 12 | Pain (VAS), function, tendon structure | 24 | Pain improvements were greatest in the eccentric group. | 11 | 15 |
| Alfredson et al. 1998 | Achilles | 1. ECCT 2. CT control | 30 | 12 | Pain (VAS) | 12 | Significant improvement with ECCT | 10 | 14 |
| Alvarez et al. 2006 | Posterior tibial | 1. Strength Ex + orthoses 2. Stretching + orthoses | 39 | 12 | Pain, function (FFI) | 12 | Both groups significantly improved in pain and function over the 12-week trial period. The self-report measures showed minimal differences between the treatment groups. | 10 | 17 |
| Kearney et al. 2013 | Achilles | 1. ECCT 2. PRP injection | 20 | 12 | Pain (VAS), function (VISA-A) | 26 | Both interventions effective, with PRP having better outcomes, however there was no significant difference. | 10 | 15 |
| Tumilty et al. 2012 | Achilles | 1. ECCT 2. ECCT + LLLT | 40 | 12 | Pain (VAS), function (VISA-A) | 52 | There was no statistically significant difference in VISA-A scores between groups. | 10 | 17 |
| Yelland et al. 2011 | Achilles | 1. ECCT 2. ECCT + prolotherapy 3. prolotherapy | 43 | 12 | Pain (VAS), function (VISA-A), costs | 52 | prolotherapy and particularly ECCT combined with prolotherapy give more rapid improvements in symptoms than ECT alone but long-term VISA-A scores are similar. | 10 | 17 |
| McCormack et al. 2016 | Achilles | 1. ECCT 2. ECCT + MT | 16 | 12 | Pain (NPRS), function (VISA-A) | 52 | ECCT + MT more effective than ECCT only at improving function during both short- and long-term follow-up | 10 | 15 |
| Tumilty et al. 2016 | Achilles | 1. ECCT 1 2. ECCT 1 + LLLT 3. ECCT 2 4. ECCT 2 +LLLT | 80 | 12 | Pain, function (VISA-A) | 12 | Twice-daily exercise sessions are not necessary as equivalent results can be obtained with two exercise sessions per week. The addition of LLLT can bring added benefit. | 10 | 17 |
| Cannell et al. 2001 | Patellar | 1. ECCT 2. Isotonic Ex | 19 | 12 | Pain (VAS), return to sport | 12 | Progressive drop squats  and leg extension/curl exercises both reduced pain and enable return to sport | 11 | 14 |
| Jonsson et al. 2005 | Patellar | 1. ECCT 2, CONCT | 19 | 12 | Pain (VAS), function, (VISA-P) | 12 | eccentric, but not concentric, quadriceps training on a decline board, seems to reduce pain in PT | 10 | 15 |
| Kedia et al. 2014 | Achilles | 1. CT 2. ECCT + CT | 36 | 12 | Pain (VAS), function (SF36) | 12 | No significant differences between groups. CT and ECCT both effective. | 10 | 15 |
| Herrington et al. 2007 | Achilles | 1. ECCT + US + MT 2. US + MT | 25 | 12 | Pain, function (VISA-A) | 12 | ECCT + CT was more effective than CT alone for pain and function. | 10 | 16 |
| Houck et al. 2015 | Posterior tibial | 1. Orthosis + stretching 2. + strength Ex | 39 | 12 | Pain, function (FFI) | 12 | Both groups significantly improved in pain and function over the 12-week trial period. minimal differences between the treatment groups. | 11 | 17 |
| Dimitrios et al. 2012 | Patellar | 1. ECCT 2. ECCT + stretching | 43 | 4 | Pain, function (VISA-P) | 24 | ECCT and static stretching exercises is superior to ECCT alone to reduce pain and improve function | 11 | 17 |
| Petersen et al. 2007 | Achilles | 1. ECCT 2. Brace 3. ECCT + brace | 100 | 12 | Pain (VAS), function (AOFAS), QoL (SF-36) | 54 | The VAS score for pain, AOFAS score, and SF-36 improved significantly in all 3 groups at all 3 follow-ups, no significant difference between groups | 10 | 16 |
| Steunebrink et al. 2013 | Patellar | 1. ECCT + GTN 2. ECCT | 33 | 12 | Pain, function (VISA-P) | 24 | GTN + ECCT does not improve clinical outcome compared to placebo patches + ECCT | 10 | 15 |
| Rompe et al. 2009 | Achilles | 1. ECCT + ESWT 2. ECCT | 68 | 12 | Pain, function (VISA-A) | 52 | Combined ECCT + ESWT more effective at 4 months follow-up | 11 | 17 |
| Young et al. 2005 | Patellar | 1. ECCT step 2. ECCT decline | 17 | 12 | Pain (VAS), function (VISA-P) | 52 | Both groups improved pain and sporting function at 12 months. Decline squat more effective. | 10 | 16 |
| De Jonge et al. 2010 | Achilles | 1. ECCT 2. ECCT + night splint | 58 | 12 | Pain, function (VISA-A) | 52 | ECCT with or without a night splint improved functional outcome at 1-year. no significant difference in clinical outcome between groups. | 10 | 14 |
| Praet et al. 2019 | Achilles | 1. ECCT + collagen peptides | 20 | 26 | Pain, function (VISA-A) | 26 | Oral supplementation of collagen peptides may accelerate the clinical benefits of ECCT. | 10 | 17 |
| Rathleff et al. 2015 | Plantar heel | 1. HSRT 2. stretching | 48 | 12 | Pain, function (FFI) | 52 | HSRT superior to plantar fascia stretching for pain and function | 11 | 14 |
| Knobloch et al. 2008 | Achilles | 1. ECCT + brace 2. ECCT | 116 | 12 | Pain (VAS), function (FAOS) | 12 | No additional effect of heel brace to ECCT alone. | 10 | 11 |
| Wheeler et al. 2017 | Plantar heel | 1. General Ex 2. Ex + night splint | 40 | 12 | Pain (VAS), Function (FFI, FAAM) | 12 | Improvement in both groups, with no significant differences between groups. | 0 | 8 |
| DeJonge et al. 2011 | Achilles | 1. PRP + ECCT 2. Placebo injection + ECCT | 54 | 12 | Pain & Function (VISA-A) | 52 | Both groups improved with no additional benefit of PRP over ECCT | 6 | 11 |
| De Vos et al. 2010 | Achilles | 1. PRP + ECCT 2. Placebo injection + ECCT | 54 | 12 | Pain & Function (VISA-A) | 24 | Both groups improved with no additional benefit of PRP over ECCT | 6 | 11 |
| Warden et al. 2008 | Patellar | 1. US + ECCT 2. Placebo US + ECCT | 37 | 12 | Pain: VAS-usual, VAS-worst | 12 | US did not provide any additional benefit over placebo + ECCT. | 10 | 17 |
| Visnes et al. 2005 | Patellar | 1. ECCT 2. Normal volleyball training | 29 | 12 | Function (VISA-P) | 26 | No effect of ECCT compared with those who continued volleyball training | 10 | 15 |
| Van Ark et al. 2018 | Patellar | 1. Isometric EX 2. Isotonic EX | 29 | 4 | Tendon US, Pain (NRS), Function (VISA-P) | 4 | Tendon structural properties did not change in either group despite positive clinical outcomes. | 12 | 14 |
| Thompson et al. 2019 | Gluteal | 1. PRP injection + ECCT 2. Saline + ECCT | 48 | 4 | Pain (NRS) | 52 | No significant differences in improvements between groups. | 6 | 10 |
| Cacchio et al. 2011 | Hamstring | 1. ESWT 2. Strength Ex + stretching | 40 | 3 | Pain (VAS) | 12 | ESWT significantly superior to exercise for pain and function. | 8 | 7 |
| Munteanu et al. 2014 | Achilles | 1. ECCT + custom orthoses 2. ECCT + sham orthoses | 140 | 12 | Pain (NRS), Function (VISA-A) | 52 | Custom orthoses no more effective than sham orthoses when combined with ECCT. | 10 | 16 |
| Van der Worp et al. 2014 | Patellar | 1. F-ESWT + ECCT 2. R-ESWT + ECCT | 43 | 12 | Pain (VAS), Function (VISA-P) | 14 | Both groups improved with no significant differences between groups. | 9 | 16 |
| Romero-morales et al. 2018 | Achilles | 1.ECCT + Vibration 2. ECCT + Cryotherapy | 61 | 12 | US Rectus anterior thickness & distance | 12 | ECCT + vibration superior to cryotherapy | 10 | 15 |
| Romero-morales et al. 2020 | Achilles | 1.ECCT + Vibration 2. ECCT + Cryotherapy | 61 | 12 | Pain & Function (VISA-A) | 12 | No significant differences between groups, both improved | 10 | 15 |
| Ryan et al. 2014 | Plantar Heel | 1. PT EX 2. CSI & stretching | 56 | 12 | Pain (VAS), Function (FADI) | 12 | Both groups improved, with no significant differences between groups. | 6 | 11 |
| Riel et al. 2018 | Plantar heel | 1. Isometric EX 2. Isotonic EX 3. Walking | 20 | 3 | Pain (VAS), PPI, US PF thickness | 3 | Isometric no better than isotonic or walking for reducing pain. | 13 | 14 |
| Koszalinski et al. 2020 | Achilles | 1. DN, MT, ECCT 2. MT, ECCT | 22 | 4 | Pain (NPRS), Function (FAAM), GROC | 12 | Both groups improved, with no significant difference between groups. | 7 | 10 |
| Pearson et al. 2012 | Achilles | 1. ABI + ECCT 2. ECCT | 33 | 12 | Function (VISA-A) | 12 | Small short-term improvement with addition of ABI to ECCT | 1 | 5 |
| Wang et al. 2007 | Patellar | 1. ESWT 2. ECCT | 50 | 12 | Function (VISA-P) | 52 | ESWT more effective than standard treatment including ECCT | 1 | 3 |
| Notarnicola et al. 2013 | Achilles | 1. CHELT + ECCT 2. ESWT + ECCT | 60 | 8 | Pain (VAS), Function (RMS) | 26 | CHELT group had quicker and better pain improvement and functional recovery. | 3 | 3 |
| Dragoo et al. 2014 | Patellar | 1. PRP, DN + ECCT 2. DN + ECCT | 23 | 12 | Pain (VAS), Function (VISA-P) | 12 | Addition of PRP improves short-term recovery, but no long-term difference | 1 | 5 |
| Kaux et al. 2019 | Patellar | 1. PRP +ECCT 2.HAI + ECCT | 33 | 12 | Pain (VAS), Function (VISA-P) | 12 | Both groups effective at medium-term, only PRP lead to pain decrease associated with strength increase | 11 | 13 |
| Abat et al. 2016 | Patellar | 1. Electro PT + ECCT 2. USGET + ECCT | 60 | 8 | Pain & Function (VISA-P) | 8 | USGET + ECCT had better outcomes for pain and function | 9 | 8 |
| Biernat et al. 2014 | Patellar | 1. ECCT 2. Normal training | 28 | 12 | Pain & Function (VISA-P) | 24 | ECCT group superior for pain and function improvement | 10 | 14 |
| Rio et al. 2015 | Patellar | 1. Isometric EX 2. Isotonic EX | 6 | Single session | Pain (SLD squat, VISA-P), MVIC | Single session | A single session of isometric EX significantly reduced pain & increased MVIC compared to isotonic EX. | 12 | 13 |
| Rio et al. 2017 | Patellar | 1. Isometric EX 2. Isotonic EX | 20 | 4 | Pain (SLD squat, VISA-P) | 4 | Both groups reduced pain, Isometric EX had significantly greater immediate analgesic effects | 12 | 16 |
| Choudhary et al. 2021 | Achilles | 1. Nutrition SUPP + ECCT 2. Diclofenac + ECCT | 40 | 12 | Pain (VAS), US | 12 | Both groups improved clinical outcomes, Nutrition SUPP + ECCT was superior. | 8 | 12 |
| Cowan et al. 2021 | Gluteal | 1.MHT + EX 2. EX + placebo 3. MHT + placebo 4. Placebo | 132 | 12 | Pain & function (VISA-G), GRoC | 52 | MHT or placebo combined with EX + education was effective for improving clinical outcomes. | 10 | 17 |
| Habets et al. 2021 | Achilles | 1. Alfredson ECCT 2. Silbernagel CONCT-ECCT | 40 | 52 | Pain (VAS), Function (VISA-A) | 52 | Both groups improved clinical outcomes, with no significant difference between groups. | 10 | 16 |
| Ruffino et al. 2021 | Patellar | 1. HSRT 2. Inertial Flywheel EX | 42 | 12 | Pain & function (VISA-P) | 12 | Both groups improved clinical outcomes, with no significant difference between groups. | 13 | 17 |
| Olesen et al. 2021 | Patellar | 1. HSRT + IGF-1 injection 2. HSRT + saline | 40 | 12 | Pain (VAS), Function (VISA-P) | 52 | Both groups improved clinical outcomes, with no significant difference between groups. | 10 | 14 |
| Hasani et al. 2021 | Achilles | 1. HI-LTUT EX 2. HI-HTUT EX 3. LI-HTUT EX 4. LI-LTUT EX | 48 | 12 | Trial measures, Pain & function (VISA-A) | 12 | A fully powered RCT would be feasible, with strategies to improve adherence & fidelity required. | 13 | 18 |
| Mansur et al. 2021 | Achilles | 1. ESWT + ECCT 2. ECCT | 119 | 12 | Pain (VAS), Function (VISA-A) | 24 | Both groups improved clinical outcomes, with no significant difference between groups. | 10 | 12 |
| Sprague et al. 2021 | Patellar | 1. HSRT + PGA 2. HSRT + PFA | 15 | 12 | Trial measures, Pain & function (VISA-P) | 12 | A fully powered RCT would be feasible, both groups improved clinical outcomes. | 13 | 18 |
| Agergaard et al. 2021 | Patellar | 1. HSRT 2.M-HSRT | 44 | 12 | Pain (NRS-P), Function (VISA-P) | 52 | Both groups improved clinical outcomes, with no significant difference between groups. | 13 | 17 |
| Lopez-Royo et al. 2021 | Patellar | 1, DN + ECCT 2. PNE + ECCT 3. ECCT | 48 | 10 | Pain (VAS), Function (VISA-P) | 22 | All groups improved clinical outcomes, with no significant difference between groups. | 10 | 14 |
| Abdelkader et al. 2021 | Achilles | 1. ESWT + ECCT 2. ECCT + SHAM | 50 | 4 | Pain (VAS), Function (VISA-A) | 56 | Both groups improved clinical outcomes, combined group had superior outcomes. | 11 | 11 |
| Van der Vlist et al. 2020 | Achilles | 1. HVIGI + ECCT 2. Placebo + ECCT | 80 | 24 | Pain & Function (VISA-A) | 24 | Both groups improved clinical outcomes, with no significant difference between groups. | 12 | 17 |
| Breda et al. 2020 | Patellar | 1. PTLE 2. ECCT | 76 | 24 | Pain & Function (VISA-P) | 24 | PTLE was superior for improving clinical outcomes compared to ECCT. | 10 | 17 |
| Rabusin et al. 2021 | Achilles | 1. Heel lifts 2. ECCT | 100 | 12 | Pain & Function (VISA-A) | 12 | Both groups improved clinical outcomes, heel lifts group had superior outcomes. | 10 | 17 |
| Solomons et al. 2020 | Achilles | 1. DN + EX 2. Sham DB + EX | 52 | 12 | Pain & Function (VISA-A) | 52 | Both groups improved clinical outcomes, with no significant difference between groups. | 1 | 11 |
| Ramon et al. 2020 | Gluteal | 1. F-ESWT + EX 2. Sham + EX | 103 | 4 | Pain (VAS), Function RMS), Harris hip score | 26 | F-ESWT combined with EX was superior for improving clinical outcomes, with a success rate of 87% at last follow-up. | 10 | 12 |
| Scott et al. 2019 | Patellar | 1. LR-PRP + HSRT 2. LP-PRP + HSRT 3. Saline + HSRT | 57 | 6 | Pain & Function (VISA-P), GRoC | 52 | PRP injections + HSRT no more effective than saline + HSRT for improving clinical outcomes. | 1 | 5 |
| Stefansson et al. 2019 | Achilles | 1. PM 2. ECCT 3. Both combined | 60 | 4 | Pain & Function (VISA-A) | 24 | All groups improved clinical outcomes, with no significant difference between groups. | 10 | 14 |
| Boesen et al. 2017 | Achilles | 1. HVIGI + ECCT 2. PRP + ECCT 3. Saline + ECCT | 60 | 6 | Pain (VAS), Function (VISA-A) | 24 | Treatment with HVIGI or PRP, with ECCT was more effective for improving clinical outcomes compared to saline + ECCT. | 10 | 15 |
| Chesterton et al. 2021 | Plantar heel | 1. Advice 2. Advice + EX 3. Advice + orthoses 4. Advice, EX & orthoses | 82 | 12 | Pain (NRS-P), Function (FFI), trial measures | 12 | A fully powered RCT would be feasible | 2 | 14 |
| Rasenberg et al. 2020 | Plantar heel | 1. Education + EX 2. Education, EX, insoles 3. Education, EX, sham insoles | 185 | 12 | Pain (NRS-P), Function (FFI), | 12 | All groups improved clinical outcomes, with no significant difference between groups. | 1 | 0 |
| Johannsen et al. 2020 | Plantar heel | 1. Surgery + strength EX 2. CSI + strength EX | 30 | 12 | Pain (VAS), Function (FFI) | 104 | Surgery + strength EX was superior for improving clinical outcomes. | 4 | 8 |
| Thong-On et al. 2019 | Plantar heel | 1. stretching 2. Strength EX | 84 | 8 | Pain (VAS) | 8 | Both groups improved clinical outcomes, with no significant difference between groups. | 10 | 17 |
| Cil et al. 2019 | Plantar heel | 1. Outpatient RX 2. Home EX | 47 | 8 | Pain (VAS), Function (FFI) | 8 | Both groups improved clinical outcomes, with the outpatient group having superior outcomes. | 9 | 10 |
| Kamonseki et al. 2016 | Plantar heel | 1. Foot EX 2. Foot & hip EX 3. Stretching | 83 | 8 | Pain (VAS), function (FAOS) | 8 | All groups improved clinical outcomes, with no significant difference between groups. | 10 | 13 |
| Brown et al. 2006 | Achilles | 1. Aprotinin + ECCT 2. Placebo + ECCT | 26 | 12 | Pain & Function (VISA-A) | 52 | Both groups improved clinical outcomes, with no significant difference between groups. | 1 | 1 |
| Niesen-Vertommen et al. 1992 | Achilles | 1. ECCT 2. CONCT | 17 | 12 | Pain (VAS) | 12 | ECCT was superior for improving clinical outcomes | 10 | 17 |
| Jensen et al. 1989 | Patellar | 1. Stretching 2. Stretching + Isokinetic ECCT | 8 | 31 | Pain (VAS), Quad strength | 8 | Quadriceps strength increased but knee pain increased with ECCT compared to healthy controls. | 11 | 16 |
| Yu et al. 2013 | Achilles | 1. ECCT 2. CONCT | 32 | 8 | Pain (VAS), muscle strength | 8 | ECCT was superior to CONCT for improving clinical outcomes | 10 | 15 |
| Wheeler et al. 2021 | Gluteal | 1. Max dose ESWT + Strength EX 2. Low dose ESWT + Strength EX | 120 | 6 | Pain & Function (VISA-G), Oxford hip score | 26 | Both groups improved clinical outcomes, with no significant difference between groups. | 7 | 13 |
| Zhang et al. 2013 | Achilles | 1. Accupunture  2. ECCT | 64 | 8 | Pain (VAS), Function (VISA-A) | 24 | Both groups improved clinical outcomes, with the acupuncture group being significantly superior. | 10 | 14 |
| Bell et al. 2013 | Achilles | 1. ABI + ECCT 2. Placebo + ECCT | 53 | 12 | Pain & function (VISA-A) | 26 | Both groups improved clinical outcomes, with no significant difference between groups. | 7 | 14 |
| Pietrosimone et al. 2020 | Patellar | 1. Isometric EX 2. Sham TENS | 28 | Single session | Pain & function (VISA-P), biomechanics | Single session | Single session isometric EX did not have acute effects on pai or landing biomechanics. | 12 | 12 |
| Holden et al. 2020 | Patellar | 1. Isometric EX 2. Dynamic EX | 21 | Single session | Pain (NRS, PPT) | Single session | Both groups immediately decreased pain but not after 45 mins, no difference between groups. | 12 | 13 |
| COHORT STUDIES |  |  |  |  |  |  |  |  |  |
| Sancho et al. 2019 | Achilles | 1. Multimodal EX program | 15 | 12 | Pain & Function (VISA-A) | 12 | Larger-scale RCT feasible, pain and function improved, high satisfaction. | 10 | 18 |
| Croisier et al. 2001 | Achilles & Patellar | 1. Isokinetic ECCT | 34 | 10 | Pain (VAS) | 10 | Isokinetic ECCT had positive short-term effects on pain & function | 10 | 16 |
| Ohberg et al. 2004 | Achilles | 1. ECCT | 25 | 12 | Pain, US | 12 | Reduction in neovascularisation associated with reduced pain following ECCT | 10 | 14 |
| Sayana et al. 2007 | Achilles | 1.ECCT | 34 | 12 | Pain & function (VISA-A) | 12 | ECCT was effective for most patients, 15 (44%) did not improve. | 11 | 16 |
| Abat et al. 2014 | Patellar | 1. EPI + Isoinertial ECCT | 33 | 12 | Pain & function (VISA-P) | 104 | Significant improvement in pain & function | 10 | 12 |
| Riel et al. 2019 | Plantar Heel | 1. HSRT, CSI, insoles, education | 20 | 8 | Pain (NRS), Function (FHSQ). | 8 | Combined HSRT & CSI was feasible and acceptable, 75% compliance. | 13 | 18 |
| Kongsgaard et al 2010 | Patellar | 1. HSRT | 8 | 12 | Pain (VAS), Function (VISA-P) | 12 | HSRT improved clinical outcomes, associated with changes toward normal fibril morphology. | 12 | 15 |
| Wetke et al. 2015 | Achilles | 1. Isotonic EX + CSI | 93 | 26 | Pain (VAS) | 26 | Significant improvements in symptoms, with 94% improving. | 10 | 16 |
| Maffulli et al. 2008 | Achilles | 1. ECCT | 45 | 12 | Pain & function (VISA-A) | 26 | Significant improvement seen in 60% of athletic patients after ECCT. | 11 | 16 |
| Shalabi et al. 2004 | Achilles | 1. ECCT | 25 | 12 | MRI, Pain & Function (Rolf & Movin 6-point scale) | 12 | ECCT resulted in decreased tendon volume, intratendinous signal and improved clinical outcomes. | 10 | 16 |
| Mansur et al. 2019 | Achilles | 1. ECCT + ESWT | 19 | 12 | Pain (VAS), Function (VISA-A, AOFAS) | 24 | Combination was effective for improving clinical outcomes. | 10 | 14 |
| Abat et al. 2015 | Patellar | 1. EPI + Isoinertial ECCT | 40 | 12 | Pain & Function (VISA-P) | 10 Years | Combination was effective for improving clinical outcomes. | 10 | 10 |
| Alfredson et al. 1999 | Achilles | 1. ECCT | 14 | 12 | Pain (VAS), BMD | 56 | ECCT led to clinical improvement but not significant BMD changes | 10 | 14 |
| O’Neill et al. 2019 | Achilles | 1. Isometric EX | 16 | Single session | Pain & Function (VISA-A), sensory response | Single session | No meaningful acute or sensory effect of intervention. | 10 | 11 |
| Ooi et al. 2019 | Achilles | 1. PRP + ECCT | 45 | 12 | Pain & Function (VISA-A), US | 52 | Achilles tendon stiffness correlated with improved clinical outcomes | 1 | 2 |
| Kaux et al. 2015 | Patellar | 1. PRP + ECCT | 20 | 5 | Pain (VAS), Function (VISA-P) | 12 | Combination was effective for improving clinical outcomes. | 3 | 4 |
| Alfredson et al. 2003 | Achilles | 1. ECCT | 6 | 12 | Pain (VAS), Function (VISA-A) | 12 | ECCT effective for improving clinical outcomes, no effect on intratendinous glutamate levels. | 10 | 14 |
| De Jonge et al. 2015 | Achilles | 1. PRP + ECCT 2. Saline + ECCT | 54 | 12 | Pain & Function (VISA-A), US | 52 | Restoration of tendon structure not required for effective symptom improvement | 3 | 2 |
| Panni et al. 2000 | Patellar | 1. Surgery 2. EX | 42 | 26 | Outcome rating | 5 years | Clinical results were good or excellent in all patients. | 1 | 2 |
| Angermann et al. 1999 | Achilles | 1. EX program | 22 | 26 | Pain & Function | 5 years | 65%had improved or resolved symptoms, 35% failed or had poor long-term outcomes. | 10 | 15 |
| Von Wehren et al. 2019 | Achilles | 1. ECCT 2. Orthokine injections | 50 | 12 | Pain & Function (VISA-A), tendon thickness | 26 | Both groups had improved clinical outcomes, injection superior for long-term outcomes. | 10 | 11 |
| Kaux et al. 2014 | Patellar | 1. ECCT & isometric EX | 30 | 12 | Pain (VAS), Function (VISA-P, IKDC) | 52 | Intervention was effective for improving clinical outcomes. | 11 | 16 |
| Wei et al. 2017 | Achilles | 1. ECCT 2. ESWT 3. ERFA | 78 | 12 | Pain (VAS), Function (VISA-A, AOFAS) | 5 years | All groups improved, ERFA led to superior clinical outcomes | 10 | 15 |
| Basas et al. 2018 | Patellar | 1. Isotonic EX + Electro stimulation | 6 | 12 | Pain (VAS) | 3 years | Intervention was effective for improving clinical outcomes. | 8 | 14 |
| Fahlstrom et al. 2003 | Achilles | 1. ECCT | 78 | 12 | Pain (VAS) | 12 | Intervention was effective for improving clinical outcomes. | 10 | 14 |
| Jonsson et al. 2008 | Achilles | 1. ECCT | 27 | 12 | Pain (VAS) | 16 | Intervention was effective for improving clinical outcomes in 67% of patients. | 10 | 14 |
| Abate et al. 2020 | Achilles | 1. ECCT + PRP | 84 | 12 | Pain & Function (VISA-A) | 26 | Age, sex, and adherence associated with improved clinical outcomes from the intervention. | 10 | 16 |
| Wheeler et al. 2020 | Achilles | 1. HVIGI + ECCT 2. ESWT + ECCT | 63 | 12 | Pain (VAS), Function (VISA-A, MOXFQ) | 12 | Both interventions effective for improving clinical outcomes, no significant difference between groups. | 1 | 3 |
| Lagas et al. 2021 | Achilles | 1. ECCT + HVIGI | 64 | 12 | Pain & Function (VISA-A) | 24 | Intervention was effective for improving clinical outcomes. | 1 | 0 |
| Robinson et al. 2021 | Achilles | 1. Combined ESWT + ECCT 2. R-ESWT + ECCT | 87 | 16 | Pain & Function (VISA-A) | 16 | Both interventions effective for improving clinical outcomes, no significant difference between groups. | 8 | 12 |
| Wheeler et al. 2021 | Plantar heel | 1. R-ESWT + EX 2. ABI + EX | 102 | 6 | Pain (NRS-P), Function (FFI, MOXFQ) | 26 | Both interventions effective for improving clinical outcomes, no significant difference between groups. | 2 | 5 |
| Mantovani et al. 2020 | Achilles | 1. Isometric EX | 22 | Single session | Pain & function (VISA-A), leg stiffness | Single session | Intervention was feasible and led to immediate improvements in pain & leg stiffness | 12 | 18 |
| CASE SERIES |  |  |  |  |  |  |  |  |  |
| Kulig et al. 2009 | Posterior tibial | 1. ECCT, orthoses | 10 | 12 | Pain (VAS), Function (FFI), GRS | 26 | Intervention was effective for improving clinical outcomes, without changes in tendon morphology. | 11 | 15 |
| Deans et al. 2012 | Achilles | 1. ACP, US, EX | 26 | 6 | Pain & Function (FAOS) | 6 | Intervention was effective for improving clinical outcomes. | 1 | 4 |
| Pavone et al. 2016 | Achilles | 1. ECCT + ESWT | 40 | 12 | Pain (VAS), Function (AOFAS) | 52 | Intervention was effective for improving clinical outcomes. | 1 | 4 |
| Romero-rodriguez et al. 2011 | Patellar | 1. Isoinertial flywheel ECCT | 10 | 6 | Pain (VAS), function (VISA-P) | 6 | Intervention was effective for improving clinical outcomes. | 12 | 15 |
| Wheeler et al. 2019 | Achilles | 1. ESWT + EX | 39 | 6 | Pain (VAS), Function (VISA-A, FAAM, RMS) | 52 | Intervention was more effective for improving clinical outcomes in insertional than non-insertional achilles tendinopathy. | 1 | 5 |
| Syverston et al. 2017 | Achilles | 1. ECCT, MT, taping | 11 | 12 | Pain (NRS), Function (VISA-A) | 12 | Intervention was effective for improving clinical outcomes. | 10 | 11 |
| Robinson et al. 2020 | Posterior tibial | 1. ESWT + EX | 10 | 16 | Pain & Function (FAAM) | 16 | Intervention was effective for improving clinical outcomes. | 8 | 11 |
| Benito et al. 2016 | Achilles | 1. ECCT, MT, electrotherapy | 5 | 6 | Pain & Function (VISA-A) | 6 | Intervention was effective for improving clinical outcomes BY 25%. | 10 | 13 |
| Silbernagel et al. 2011 | Achilles | 1. ECCT, plyometric EX | 34 | 12 | Pain & Function (VISA-A) | 5 years | Most patients fully recovered in terms of pain and function | 10 | 14 |
| Morton et al. 2014 | Patellar | 1. HVIGI + ECCT | 20 | 12 | Pain & Function (VISA-A) | 12 | Intervention was effective for improving clinical outcomes. | 1 | 5 |
| Van ark et al. 2013 | Patellar | 1. PRP + EX program | 5 | 12 | Pain & Function (VISA-A) | 26 | Intervention was effective for improving clinical outcomes. | 11 | 16 |
| Munoz Fernandez et al. 2021 | Patellar | 2. UGPE + EX | 3 | 8 | Pain (NRS-P), Function (VISA-P) | 8 | Intervention was effective for improving clinical outcomes. | 10 | 12 |
| Skovlund et al. 2020 | Patellar | 1. low load BFRT | 7 | 3 | Pain (NRS-P), Function (VISA-P) | 3 | Intervention was effective for improving clinical outcomes. | 13 | 17 |
| Jayaseelan et al. 2017 | Achilles | 1. MT + ECCT | 3 | 12 | Pain & Function (VISA-A) | 36 | Intervention was effective for improving clinical outcomes. | 10 | 12 |
| Bianco et al. 2019 | Patellar | 1. MT + EX | 3 | 12 | Pain (VAS), Function (VISA-P) | 12 | Patients improved clinical outcomes and returned to sports activity | 8 | 14 |
| CASE REPORT |  |  |  |  |  |  |  |  |  |
| Eckenrode et al. 2015 | Achilles | 1. E-STIM + ECCT | 1 | 12 | Pain & Function (VISA-A) | 12 | Patient improved clinical outcomes and returned to sports activity | 12 | 14 |
| Papa et al. 2012 | Achilles | 1. MT + ECCT | 1 | 4 | Pain (VAS), Function (LEFS) | 52 | Patient improved clinical outcomes and returned to normal activity | 10 | 13 |
| Dos Santos et al. 2016 | Plantar heel | 1. MT + Hip strength EX | 1 | 10 | Pain (NPR-S) | 10 | Patient improved clinical outcomes | 5 | 9 |
| Lee et al. 2019 | Plantar heel | 1. MT + Hip strength EX | 1 | 12 | Pain (VAS), Function (AOFAS, FFI, FAAM) | 12 | Patient improved clinical outcomes and returned to normal activity | 1 | 4 |
| Ross et al. 2017 | Achilles | 1. Osteopathy + EX | 1 | 12 | Pain (VAS), Function (VISA-A) | 12 | Patient improved clinical outcomes and returned to sports activity | 10 | 16 |
| Cuddeford et al. 2020 | Patellar | 1. BFRT | 2 | 12 | Pain (VAS), Function (VISA-P) | 12 | Patients improved clinical outcomes and returned to sports activity | 12 | 15 |
| Krueger et al. 2020 | Hamstring | 1. HSRT | 1 | 12 | Pain (VAS), self-reported function | 12 | Patient improved clinical outcomes and returned to sports activity | 11 | 15 |
| Borda et al. 2017 | Achilles | 1. MT + ECCT | 1 | 6 | Pain & function (LEFS) | 6 | Patient improved clinical outcomes and returned to sports activity | 10 | 11 |
| Rauseo et al. 2017 | Iliopsoas | 1. ECCT | 1 | 12 | Pain (VAS), Function (CHGOS) | 5 years | Patient improved clinical outcomes and returned to sports activity | 10 | 15 |
| McCormack et al. 2012 | Achilles | 1. MT + ECCT | 1 | 12 | Pain (NRS-P), Function (LEFS) | 12 | Patient improved clinical outcomes and returned to sports activity | 10 | 14 |
| Patla et al. 2015 | Posterior tibial | 1. MT + EX | 1 | 6 | Pain (NRS-P), Function (LEFS) | 6 | Patient improved clinical outcomes and returned to sports activity | 8 | 13 |
| Pinkelman et al. 2012 | Extensor hallucis longus | 1. Manually resisted EX, MT + US | 1 | 6 | Pain (VAS) | 6 | Patient improved clinical outcomes and returned to sports activity | 8 | 14 |
| Francis et al. 2020 | Achilles | 1. ESWT, ECCT, running program | 1 | 12 | Pain & Function (VISA-A, FADI) | 12 | Patient improved clinical outcomes and returned to sports activity | 10 | 15 |
| McCreesh et al. 2013 | Patellar | 1.ECCT | 1 | 8 | Pain & function (VISA-P), US | 8 | Patient improved clinical outcomes and tendon neovascularity. | 10 | 13 |
| Hensley et al. 2012 | Peroneal | 1. MT + EX | 1 | 8 | Pain (NRS-P), Function (LEFS) | 8 | Patient improved clinical outcomes and returned to normal activity | 10 | 13 |
| Cushman et al. 2015 | Hamstring | 1. ECCT | 1 | 4 | Pain & function (VISA-H) | 12 | Patient improved clinical outcomes and returned to sports activity | 10 | 12 |
| Thompson et al. 2017 | Achilles & Plantar heel | 1. EX program | 1 | 12 | Pain (VAS) | 12 | Patient improved clinical outcomes and returned to sports activity | 11 | 14 |
| Jayaseelan et al. 2014 | Hamstring | 1. EX + MT | 2 | 10 | Pain (NRS-P) Function (LEFS) | 10 | Patients improved clinical outcomes and returned to sports activity | 10 | 14 |
| Dumont et al. 2006 | Patellar | 1. ECCT | 4 | 6 | Pain (VAS) | 6 | Patients improved clinical outcomes but did not achieve full recovery. | 10 | 16 |
| Silva et al. 2015 | Patellar | 1. Hip strength EX | 1 | 8 | Pain (VAS), Function (VISA-P) | 26 | Patient improved clinical outcomes and returned to sports activity | 11 | 14 |
| McCormack et al. 2012 | Hamstring | 1. MT + ECCT | 1 | 8 | Pain (NRS-P) Function (LEFS) | 8 | Patient improved clinical outcomes by 95% and returned to sports activity | 10 | 13 |
| Van Rooy et al. 2009 | Gluteal | 1. MT + ECCT | 1 | 12 | Pain (VAS) | 12 | Patient improved clinical outcomes and was pain-free after intervention | 10 | 12 |
| Greene et al. 2002 | Achilles | 1. Ex program, orthosis | 1 | 11 | Pain (VAS) Function (LEFS) | 11 | Patient improved clinical outcomes | 8 | 14 |
| Rowan et al. 2013 | Patellar | 1. PRP + ECCT | 1 | 4 | Pain (VAS), Function (VISA-P) | 26 | Patient improved clinical outcomes and returned to sports activity | 8 | 10 |
| Goldman et al. 2010 | Patellar | 1.ECCT | 1 | 6 | Pain (VAS), Function (VISA-P, LEFS) | 6 | Patient improved clinical outcomes and returned to sports activity | 11 | 14 |
| Cuddeford et al. 2018 | Achilles | 1. ECCT | 1 | 10 | Pain & function (VISA-A) | 10 | Patient improved clinical outcomes | 8 | 15 |
| OTHER |  |  |  |  |  |  |  |  |  |
| Longitudinal with control  Masood et al. 2014 | Achilles | 1. ECCT | 20 | 12 | Pain (VAS), Function (VISA-A), EMG | 12 | Intervention was effective for improving clinical outcomes. | 10 | 16 |
| Before-after design  Gardin et al. 2010 | Achilles | 1. ECCT | 24 | 12 | Pain & Function (Rolf & Movin), MRI | 18 | Intervention was effective for improving clinical outcomes & decreased intratendinous signal. | 10 | 10 |
| Case control  Langberg et al. 2007 | Achilles | 1. ECCT | 12 | 12 | Pain (VAS) | 12 | Intervention was effective for improving clinical outcomes & Achilles collagen synthesis. | 10 | 15 |
| Quasi experimental (non-randomised)  Stasinopoulos et al. 2013 | Achilles | 1. Alfredson ECCT 2. Stanish protocol | 41 | 12 | Pain & Function (VISA-A) | 12 | Alfredson ECCT protocol was superior for improving pain & function | 11 | 17 |
| Quasi experimental (non-randomised)  Kanniappan et al. 2020 | Achilles | 1. ECCT 2. Isometric EX | 40 | 4 | Pain (VAS), Function (VISA-A) | 4 | Both groups improved clinical outcomes, with no significant differences between groups. | 11 | 11 |
| Quasi experimental (non-randomised)  Purdam et al. 2004 | Patellar | 1. ECCT – flat squat 2. ECCT – DSL squat | 17 | 12 | Pain (VAS) | 12 | DSL squats more effective than standard flat squats for improving clinical outcomes. | 10 | 14 |
| Quasi experimental (non-randomised  Van der Vlist et al. 2020 | Achilles | 1. Isometric EX (PF) 2. Isometric EX (DF) 3. Isotonic EX 4. rest | 91 | Single session | Pain (VAS) | Single session | Isometric EX did not result in immediate pain relief. | 12 | 17 |
| Before-after design  Morgan et al. 2018 | Patellar | 1. EX program | 16 | 12 | Pain (VAS), Function (VISA-P), EMG | 12 | Intervention was effective for improving clinical outcomes. | 4 | 6 |
| Observational prospective clinical trial  De Vos et al. 2012 | Achilles | 1. ECCT | 25 | 16 | Pain & Function (VISA-A), US | 24 | Intervention was effective for improving clinical outcomes but did not change tendon structure. | 11 | 14 |
| Retrospective case control  Park et al. 2021 | Achilles | 1. ECCT 2. Control | 28 | 12 | Pain (VAS) | 12 | ECCT was less effective for improving clinical outcomes in patients with metabolic syndrome. | 11 | 14 |
| Case control  Ram et al. 2013 | Achilles | 1. ECCT 2. ECCT (healthy) | 48 | 12 | Pain & Function (VISA-A), satisfaction | 12 | Intervention was effective for improving clinical outcomes, but satisfaction was low. | 10 | 13 |
| Retrospective chart review  Vander Doelen et al. 2020 | Patellar | 1. Multimodal rehabilitation INC EX, DN, ESWT. MT | 9 | 18-32 | Pain (NRS-P), Function (VISA-P) | 18-32 | Patients improved clinical outcomes and returned to sports activity | 11 | 14 |

**Abbreviations:** ECCT: eccentric training, ESWT: extracorporeal shockwave therapy, DN: dry needling; MT: manual therapy, EX: exercise; VAS: visual analogue scale, NRS-P: pain numeric rating scale, VISA-A: Victorian Institute of Sport Assessment – Achilles, VISA-P: Victorian Institute of Sport Assessment – Patellar, VISA-G: Victorian Institute of Sport Assessment – Gluteal, VISA-H: Victorian Institute of Sport Assessment – Hamstring, FFI: Foot Function Index, LEFS: Lower Extremity Function Scale, WKS: weeks, US: ultrasound, PRP: platelet-rich plasma, HSRT: heavy slow resistance training: CONCT: concentric training, E-STIM: electrical stimulation, CSI: corticosteroid injection: LLLT: low-level laser therapy, BFRT: blood flow restriction training, FADI: Foot and ankle disability index, AOFAS: American orthopaedic foot and ankle score, UGPE: ultrasound guided percutaneous electrolysis, HVIGI: high-volume image guided injection: MRI: magnetic resonance imaging: RMS: Roles and Maudsley score, MHT: menopause hormone therapy, PPI: pain pressure intensity; FAAM: foot and ankle ability measure.

**APPENDIX 3: Table 5: Application of resistance training principles**

| Author | Spec  ificity | Over  load | Progression + method | Individualised + method | Frequency (d/wk) | Intensity | Time (min) | Sets | Reps | Exercise mode/type | Adherence | RTP /8, Total /10 |
| --- | --- | --- | --- | --- | --- | --- | --- | --- | --- | --- | --- | --- |
| Beyer et al. 2015 | Y | Y | Y, increase resistance/ load | Y, pain response 4-5/10 | 3 | 15RM – 6RM | 107 x wk (HSRT) 308 x wk (ECCT) | 3-4 | 15-6 | Heel raises, with external weights | Y, diary (78-92%) | 7, 9 |
| Kongsgaard et al. 2009 | Y | Y | Y, increase resistance | Y, pain response 3/10 | 3 | 15RM – 6RM | NR | 3-4 | 15-6 | DSL squats, squat, leg press, hack squat, with external weights | Y, diary (89-91%) | 7, 9 |
| Riel et al. 2019 | Y | Y | Y, increase resistance or volume | Y, as many sets as possible | 3 | 8RM – 12RM | tut | 3-5, AMAP | 8-12 | Heel raises, loaded backpack | Y, diary, 29% not returned | 7, 9 |
| Stevens & Tan 2014 | Y | Y | Y, increase resistance or volume | Y, as many reps as possible | 7, 2xd | 15RM | NR | 2 x 6 (12) | 15 (180 total) | Heel raises (straight leg & bent knee), loaded backpack | Y, diary, above 75% | 7, 9 |
| Da Cunha et al. 2012 | Y | Y | Y, increase resistance (5kg inc) | Y, pain response | 3 | 15RM | NR | 3 | 15 | Eccentric decline squat | NR | 8, 8 |
| Kulig et al. 2009 | Y | Y | Y, increase resistance (0.9kg conforce spring) | Y, increase isokinetic resistance as able | 7, 2xd | 15RM | NR | 2 x 3 (6) | 15 (180) | Isokinetic resisted horizontal adduction with plantar flexion | Y, diary, 68% (39-98) | 8, 10 |
| Bahr et al. 2006 | Y | Y | Y, increase resistance (5kg inc) | Y, pain response less 3/10, increase 5kg | 7, 2xd | 15RM | NR | 2 X 3 (6) | 15 (180) | DSL squat, loaded backpack | NR | 8, 8 |
| Lee et al. 2020 | Y | Y | Y, increase resistance (5kg inc) | Y, pain response 4/10, increase 5kg | 7, 2Xd | 15RM | NR | 2 X 3 (6) | 15 (180) | DSL squat, loaded backpack | Y, diary | 8, 9 |
| Frohm et al. 2007 | Y | Y | Y, increase resistance (5kg inc) | Y, pain response 5/10, increase 5kg | 1.2 2. 7, 2xd | 15-16RM | 70 mins x session | 3-4 | 15-16 | 1. The Bromsman eccentric overload training device 2. DSL squat, loaded backpack | NR | 8,8 |
| Silbernagel et al. 2001 | Y | Y | Y, increase resistance, volume, speed & difficultly | Y, pain response 5/10 | 7 | 5-15RM | NR | 3 | 5-15 | Double and single leg Slow Heel raises, fast rebounding heel raises | Y, diary | 7, 8 |
| Balius et al. 2016 | Y | NR | NR | NR | 7, 2xd | 15RM | NR | 2 X 3 (6) | 15 (180) | Alfredson heel raises, straight & bent knee | PT recorded; 70% minimum allowed | 2, 4 |
| Mafi et al. 2001 | Y | Y | Y, increase resistance | Y, pain response | 7, 2xd | 15RM | NR | 2 X 3 (6) | 15 (180) | Alfredson heel raises, straight & bent knee, loaded with backpack or weight machines | NR | 7, 7 |
| Norregaard et al. 2007 | Y | Y | Y, increase resistance (5kg inc) | Y, pain response, increase 5kg | 7, 2xd | 15RM | NR | 2 X 3 (6) | 15 (180) | Alfredson heel raises, straight & bent knee, loaded with backpack | Y. diary, results NR | 8, 9 |
| Stasinopolous et al. 2004 | Y | Y | Y, increase resistance | Y, pain response | 7, 2xd | 15RM | NR | 2 X 3 (6) | 15 (180) | DSL squat, handheld external weights | NR | 7, 7 |
| De Vos et al. 2007 | Y | Y | Y, increase resistance | Y, pain response | 7, 2xd | 15RM | NR | 2 X 3 (6) | 15 (180) | Alfredson heel raises, straight & bent knee, loaded with backpack or weight machines | Y, diary, (70-74%) | 7, 9 |
| Johannsen et al. 2019 | Y | UC | UC | NR | 3 | NR | NR | NR | NR | (1) heel-raises, (2) flexion of the first toe against elastic band. (3) Inversion of the foot against elastic band | NR | 2, 2 |
| MacDonald et al. 2019 | Y | Y | Y, increase resistance (5kg inc) | Y, pain response 5/10, increase 5kg, correct technique | 7, 2xd | 15RM | NR | 2 X 3 (6) | 15 (180) | DSL squat eccentric protocol with addition of isotonic hip exercise, loaded backpack | Y, diary, 42.5% full | 8, 10 |
| Gatz et al. 2020 | Y | Y | Y, increase resistance | Y, pain response | 7, 2 X D | 15RM | NR | 2 X 3 (6) | 15 (180) | Alfredson eccentric heel raise protocol + isometric exercise | Y, verbal, NR | 7, 8 |
| Ganderton et al. 2018 | Y | Y | Y, increase difficulty | Y, individual ability determined progression | 7, 2 x d | 5-15RM | 30MIN X D | 2-4 | 5-15 | isometric loading of gluteals, and kinetic chain  strength exercises | Y, diary, 75% | 7, 9 |
| Silbernagel et al. 2007 | Y | Y | Y, Increase resistance, volume, and speed of exercises | Y, Increased resistance, volume, and speed guided by Pain response | 7 | 10-20RM | NR | 3 | 10-20 | 2-legged, 1-legged, eccentric, and fast rebounding toe raises, plyometric exercise. Loaded with backpack or weight machine | Y, diary | 7, 8 |
| Clifford et al. 2019 | Y | Y | Y, increase resistance band strength | Y, pain response 5/10 | 7 | 6-10RM | 6min TUT x d | 3-6 | 6-10 | Isotonic & isometric hip abduction, loaded with bands | Y, diary, (58-70%) | 7, 9 |
| Stergioulas et al. 2008 | Y | Y | Y, increase resistance (4kg inc) | Y, pain response 5/10 | 4 | 12RM | NR | 12 | 12 | Eccentric heel raise, knee straight & flexed, loaded backpack | Y, diary (85-100%) | 8, 10 |
| Rompe et al. 2008 | Y | Y | Y, increase resistance (5kg inc) | Y, pain response, increase 5kg | 7, 2 X D | 10-15RM | NR | 3 X 2 (6) | 10-15 (180) | Alfredson eccentric heel raise, knee straight & flexed, loaded backpack | Y, verbal, NR | 8, 9 |
| Van Ark et al. 2016 | Y | Y | Y, increase resistance 2.5% per week | Y, pain response, correct technique, 2.5% increase | 4 | isometric (80% 1RM) isotonic (80% 8RM) | NR | 4-5 | 5-8 | Leg extension machine, external weight. Audio used for speed tempo | NR | 8, 8 |
| Roos et al. 2004 | Y | Y | Y, increase resistance | Y, pain response | 7, 2 X D | 15RM | NR | 1-3 | 15 (180) | Modified Alfredson eccentric heel raise, knee straight & flexed, loaded backpack | Y, diary (50-75%) | 7, 9 |
| Chester et al. 2008 | Y | Y | Y, increase resistance | Y, pain response | 7 | 15RM | NR | 3 X 2 (6) | 15 (90) | Modified Alfredson eccentric heel raise, knee straight & flexed, loaded backpack | NR | 7, 7 |
| Rompe et al. 2007 | Y | Y | Y, increase resistance (5kg inc) | Y, pain response, increase 5kg | 7, 2 X D | 10-15RM | NR | 3 X 2 (6) | 10-15 (180) | Modified Alfredson eccentric heel raise, knee straight & flexed, loaded backpack | NR | 8, 8 |
| Thijs et al. 2017 | Y | Y | Y, increase resistance | Y, pain response, 4/10 | 7, 2 X D | 15RM | NR | 3 X 2 (6) | 15 (180) | DSL eccentric squat, loaded backpack | NR | 7, 7 |
| Horstmann et al. 2013 | Y | Y | Y, increase resistance + volume, based on fatigue | Y, increase resistance + volume, based on fatigue | 7 | 15RM | NR | 3-4 | 15 | Modified Alfredson eccentric heel raise, knee straight & flexed, loaded backpack | NR | 7, 7 |
| Alfredson et al. 1998 | Y | Y | Y, increase resistance | Y, pain response | 7, 2 x d | 15RM | NR | 3 X 2 (6) | 15 (180) | Modified Alfredson eccentric heel raise, knee straight & flexed, loaded backpack or weight machine | NR | 7, 7 |
| Alvarez et al. 2006 | Y | Y | Y, increase resistance (elastic bands) and volume | Y, increase resistance based on pain response + correct technique | 7, 2 X D | 30RM | NR | 3 | 30 | Isotonic exercise with elastic bands, increased resistance (elastic bands strength) 1. Bilateral heel raises 2. Ankle plantar flexion with adduction and  Inversion.  3. Unilateral heel raises (standing) | Y, diary (79%) | 7, 9 |
| Kearney et al. 2013 | Y | Y | Y, progress from DL to SL with increased resistance | Y, pain response, progress from DL to SL with increased load | 7, 2 x d | 15RM | NR | 3 X 2 (6) | 15 (180) | Modified Alfredson eccentric heel raise, knee straight & flexed, loaded backpack, DL progressing to SL | NR | 7, 7 |
| Tumilty et al. 2012 | Y | Y | Y, increase resistance | Y, pain response | 7, 2 x d | 15RM | NR | 3 X 2 (6) | 15 (180) | Modified Alfredson eccentric heel raise, knee straight & flexed, loaded backpack | Y, diary (70%) | 7, 9 |
| Yelland et al. 2011 | Y | Y | Y, increase resistance | Y, pain response 4/10 | 7, 2 x d | 15RM | NR | 3 X 2 (6) | 15 (180) | Modified Alfredson eccentric heel raise, knee straight & flexed, loaded backpack | Y, diary | 7, 8 |
| McCormack et al. 2016 | Y | Y | Y, increase resistance | NR | 7, 2 X D | 15RM | NR | 3 X 2 (6) | 15 (180) | Modified Alfredson eccentric heel raise, knee straight & flexed, loaded backpack | NR | 5, 5 |
| Tumilty et al. 2016 | Y | Y | Y, increase resistance | Y, pain response, 4/10 | 2 | 15RM | NR | 3 X 2 (6) | 15 (180) | Modified Alfredson eccentric heel raise, knee straight & flexed, loaded backpack. 2Xwk V D | Y, diary, 70-100% | 7, 9 |
| Cannell et al. 2001 | Y | Y | Y, increase resistance with fixed loading protocol & external weight | Y, pain response | 5 | 10-20RM | NR | 3 | 10-20 | Progressive drop squats and leg extension/curl exercises, fixed loading protocol, external weights | NR | 8, 8 |
| Jonsson et al. 2005 | Y | Y | Y, increase resistance | Y, self-acceptable pain response | 7, 2 X D | 15RM | NR | 3 X 2 (6) | 15 (180) | Eccentric v concentric DSL squat, loaded backpack | NR | 7, 7 |
| Mellor et al. 2018 | Y | Y | Y, increase diffciculty/ intensity  (BORG) | Y, pain response 5/10, BORG scale (13-17 target) | 7 | BORG (13-17) | 30 min x session | 1-2 | 3-15 | Comprehensive progressive exercise program targeting hip muscles, monitored by pain response and BORG scale. External load NR. Spring resistance for hip abduction | Y, diary, 80% | 8, 10 |
| Kedia et al. 2014 | Y | Y | Y, increase resistance | Y, exercise difficultly, increase resistance | 7, 2 x d | 15RM | NR | 3 X 2 (6) | 15 (180) | Modified Alfredson eccentric heel raise, knee straight & flexed, loaded backpack | Y, diary, NR | 7, 8 |
| Herrington et al. 2007 | Y | Y | Y, increase speed and resistance | Y, increase speed and resistance based on pain response | 7, 2 X D | 15RM | NR | 3 X 2 (6) | 15 (180) | Modified Alfredson eccentric heel raise, knee straight & flexed, loaded backpack | Y, diary, NR | 7, 8 |
| Houck et al. 2015 | Y | Y | Y, increase resistance – elastic bands strength | Y, increase resistance based on pain response & Ex technique | 7, 2 X D | 30RM | 30 min x session | 3 X 2 (6) | 30 X 3 X 3 (180) | Bilateral & unilateral heel raises, ankle plantarflexion with adduction & inversion. Resistance bands | Y, diary (79%) | 7, 9 |
| Dimitrios et al. 2012 | Y | Y | Y, increase resistance with handheld weights | Y, pain response | 5 | 15RM | NR | 3 | 15 | Eccentric DSL squat, handheld weights | Y, diary, NR | 7, 8 |
| Petersen et al. 2007 | Y | Y | Y, increase resistance | Y, pain response | 7, 3 x D | 15RM | NR | 3 X 3 (9) | 15 (270) | Modified Alfredson eccentric heel raise, knee straight & flexed, loaded backpack | Y, diary, NR | 7, 8 |
| Steunebrink et al. 2013 | Y | Y | Y, increase resistance (5kg inc) | Y, pain response, 3/10 = increase load | 7, 2 x d | 15RM | NR | 3 X 2 (6) | 15 (180) | Modified Alfredson - Eccentric DSL squat | Y, diary (70%) | 8, 10 |
| Rompe et al. 2009 | Y | Y | Y, increase resistance (5kg inc) | Y, pain response | 7, 2 X D | 15RM | NR | 3 X 2 (6) | 10-15 (180) | Modified Alfredson eccentric heel raise, knee straight & flexed, loaded backpack | NR | 8, 8 |
| Young et al. 2005 | Y | Y | Y, increase speed, then resistance (5kg inc) | Y, pain response | 7. 2 x d | 15RM | NR | 3 X 2 (6) | 15 (180) | Modified Alfredson DSL squat, loaded backpack | Y, diary (72%) | 8, 10 |
| De Jonge et al. 2010 | Y | Y | Y, increase resistance | Y, pain response | 7, 2 x d | 15RM | NR | 3 X 2 (6) | 15 (180) | Modified Alfredson eccentric heel raise, knee straight & flexed, loaded backpack or weight machine | Y, diary | 7, 8 |
| Praet et al. 2019 | Y | Y | Y, increase speed, then resistance (5kg inc until max 60kg) | Y, pain response | 7, 2 X D | 15RM | NR | 3 X 2 (6) | 15 (180) | Modified Alfredson eccentric heel raise, knee straight & flexed, loaded backpack | Y, diary (78-84%) | 8, 10 |
| Rathleff et al. 2015 | Y | Y | Y, increase resistance | NR | 3 | 12-8RM | NR | 3-5 | 12-8 | Heel raise on step with toes maximally dorsiflexed on towel | NR | 5, 5 |
| Knobloch et al. 2008 | Y | NR | NR | NR | 7, 2 X D | 15RM | NR | 3 X 2 (6) | 15 (180) | Modified Alfredson eccentric heel drop, knee straight & flexed | NR | 2, 2 |
| Wheeler et al. 2017 | Y | NR | NR | NR | NR | NR | NR | NR | NR | stretching, calf & foot muscle strengthening and balance exercises. | NR | 2, 2 |
| De Jonge et al. 2011 | Y | NR | NR | Y, pain response | 7 | NR | NR | NR | 180 | Alfredson eccentric heel drop, knee straight & flexed, | Y, Verbal | 4, 5 |
| De Vos et al. 2010 | Y | NR | NR | Y, pain response | 7 | NR | NR | NR | 180 | Alfredson eccentric heel drop, knee straight & flexed, | Y, Verbal | 4, 5 |
| Warden et al. 2008 | Y | Y | Y, increase resistance with hand weights | Y, pain response | 7 | 15RM | NR | 3 | 15 (45) | Modified Alfredson DSL squat, hand weights | Y, diary, 65% | 7, 9 |
| Visnes et al. 2005 | Y | Y | Y, increase resistance (5kg inc) | Y, pain response | 7, 2 X D | 15RM | NR | 3 X 2 (6) | 15 (90) | Modified Alfredson DSL squat, loaded backpack | Y, diary | 8, 9 |
| Van Ark et al. 2018 | Y | Y | Y, increase resistance (2.5% per week) | Y, pain response | 4 | 8RM | NR | 4X2 | 8X2 | Leg extension machine | NR | 8, 8 |
| Thompson et al. 2019 | Y | NR | NR | Y, pain response | 7, 2 X D | 10-15RM | NR | 1 X 2 | 10-15 | leg lunges, single stance knee bends, and side lying eccentric flexion, side bending and extension | Y, NR | 4, 5 |
| Cacchio et al. 2011 | Y | Y | NR | NR | 3 | 6-10RM | NR | 3-4 | 6-10 | Loaded with weights: leg curls, hip flexion & extension, deadlift, lunge, half squat, countermovement jump | NR | 4, 4 |
| Munteanu et al. 2014 | Y | Y | Y, increase resistance (5kg inc) | Y, pain response | 7, 2 X D | 15RM | NR | 3 X 2 | 15 | Alfredson eccentric heel-drop protocol | Y, diary (57%) | 8, 10 |
| Van der Worp et al. 2014 | Y | Y | Y, increase resistance | Y, pain response | 5 | 15RM | NR | 3 X 2 | 15 | DSL squat, loaded backpack (Visnes protocol) | Y, diary | 7, 8 |
| Romero-morales et al. 2018 | Y | Y | Y, increase resistance | Y, pain response | 7, 2 X D | 15RM | NR | 3 X 2 (6) | 15 (90) | Modified Alfredson heel-drop protocol | Y, diary | 7, 8 |
| Romero-morales et al. 2020 | Y | Y | Y, increase resistance | Y, pain response | 7, 2 X D | 15RM | NR | 3 X 2 (6) | 15 (90) | Modified Alfredson heel-drop protocol | Y, diary | 7, 8 |
| Ryan et al. 2014 | Y | NR | NR | NR | 7 | 15RM | NR | 3-5 | 15 | Forefoot extension, ankle inversion & eversion, SL standing, stretching. | Y, diary | 2, 3 |
| Riel et al. 2018 | Y | Y | Y, increase resistance | Y, increase resistance individually | 3 | 8RM | 64/S set, 256/S total | 4 | 8 | Heel-raise with loaded backpack | NR | 7, 7 |
| Koszalinski et al. 2020 | Y | NR | NR | NR | NR | 15RM | NR | 3 | 15 | Alfredson eccentric heel-drop, Ankle adduction, Towel crunches | NR | 2, 2 |
| Pearson et al. 2012 | Y | Y | Y, increase resistance | Y, pain response | NR | NR | NR | NR | NR | Alfredson eccentric heel-drop, no details given | NR | 7, 7 |
| Wang et al. 2007 | Y | NR | NR | NR | NR | NR | NR | NR | NR | Eccentric strengthening of quadriceps and hamstrings | NR | 2, 2 |
| Notarnicola et al. 2013 | Y | NR | NR | NR | NR | NR | NR | 3 | 10 | Eccentric exercise unspecified | NR | 2, 2 |
| Dragoo et al. 2014 | Y | NR | NR | NR | NR | NR | NR | NR | NR | Eccentric exercise unspecified | NR | 2, 2 |
| Kaux et al. 2019 | Y | Y | Y, increase volume | NR | 3 | 15-20RM | NR | 5-7 | 15 | Bodyweight eccentric wall squat | NR | 5, 5 |
| Abat et al. 2016 | Y | NR | NR | NR | NR | 15RM | 15min | 3 | 15 | Eccentric DSL squat | NR | 2, 2 |
| Biernat et al. 2014 | Y | Y | Y, increase difficulty | Y, pain response | 7 | 15RM | NR | 3X2 (6) | 15 (90) | Eccentric DSL squat | NR | 7, 7 |
| Rio et al. 2015 | Y | Y | NR | NR | Single session | 8RM | NR | 4 | 8 | Biodex (isometric) Leg extension machine (isotonic) | Y, supervised | 4, 5 |
| Rio et al. 2017 | Y | Y | Y, increase resistance (2.5% weekly) | Y, fatigue | 4 | 8RM | NR | 4 | 8 | Leg extension machine | Y, supervised | 8, 9 |
| Holden et al. 2020 | Y | Y | NR | NR | Single session | 8RM | NR | 3 | 8 | Biodex (isometric) Leg extension machine (isotonic) | Y, supervised | 4, 5 |
| Choudhary et al. 2021 | Y | Y | Y, increase repetitions | Y, pain response | 7, 3 X D | 15RM | NR | 3 | 15 (45) | ECCT – no details | NR | 7, 7 |
| Cowan et al. 2021 | Y | Y | Y, individual ability determined progression | Y, increase difficultly | 7, 2 x D | 5-15RM | 15min x 2 (30) | 2-4 | 5-15 | isometric loading of gluteals, and kinetic chain  strength exercises | Y, diary (70-94%) | 7, 9 |
| Habets et al. 2021 | Y | Y | Y, increase resistance (5kg inc in backpack - AG), + increase speed (SG) | Y, pain response | 7, 2 x D (AG) | 15RM (AG) | NR | 6 (AG)  3 (SG) | 180 (AG)  15 (SG) | Alfredson ECCT heel drop VS Silbernagel CONCT-ECCT heel raise | Y, diary, 74% (AG) 77% (SG) | 8, 10 |
| Ruffino et al. 2021 | Y | Y | Y, increase resistance | Y, pain response | 3 | 6-15RM | 50MIN | 4 | 6-15 | HSRT (modified Kongsgaard protocol): squat, hack squat, leg press. Flywheel: squat, leg press, knee extension. | Y, diary, 88% (HSRT), 90% (Flywheel) | 7, 9 |
| Olesen et al. 2021 | Y | Y | Y, increase resistance | Y, pain response | 3 | 6-15RM | NR | 4 | 6-15 | HSRT (modified Kongsgaard protocol): squat, knee extension, leg press. | NR | 7, 7 |
| Hasani et al. 2021 | Y | Y | Y, increase resistance | Y, pain response & difficultly | 3 | 6-18RM | 39-53MINS | 4 | 6-18 | Seated & standing calf raises on smith machine: high (6 RM) or low intensity (18 RM) exercise, performed with either high (6 s) or low (2) time-under-tension. | Y, diary, 49-68% | 7, 9 |
| Mansur et al. 2021 | Y | Y | NR | NR | 7, 2 X D | 15RM | NR | 3 x 2 x 2 (12) | 15 x 3 x 2 x 2 (180) | Modified Alfredson heel drop protocol | NR | 4, 4 |
| Sprague et al. 2021 | Y | Y | Y, increase resistance | Y, pain response | 3 | 6-15RM | NR | 4 | 6-15 | HSRT (modified Kongsgaard protocol): squat, knee extension, leg press. | Y, diary (67-86%) | 7, 9 |
| Agergaard et al. 2021 | Y | Y | Y, increase resistance (% of 1RM) | Y, pain response | 3 | 55-90% 1RM | NR | 3-5 | 4-15 | HSRT: leg press, knee extension | Y, diary, 78-86% | 8, 10 |
| Lopez-Royo et al. 2021 | Y | Y | Y, increase speed | Y, pain response | 7, 2 X D | 15RM | NR | 3 | 15 | Young ECCT Protocol: DSL squat | NR | 7, 7 |
| Abdelkader et al. 2021 | Y | NR | NR | NR | 7, 2 X D | 15RM | NR | 3 | 15 | Modified Alfredson heel drop protocol, 4 weeks only | NR | 2, 2 |
| Van der Vlist et al. 2020 | Y | Y | Y, increase resistance (backpack or weights) | Y, pain response | 7 | 15RM | NR | 3 | 15 | Silbernagel protocol: isometric, CONCT, ECCT, plyometric, calf raises, | Y, diary, 76% | 7, 9 |
| Breda et al. 2020 | Y | Y | Y, increase resistance & difficultly | Y, pain response | 3-7 | 6-15RM, 70% MVIC (isometric) | NR | 4 | 6-15 | ECCT: DSL squat, PTLE: isometric, isotonic, plyometric EX, leg press, leg extension, sport specific, hip abduction & extension EX | Y, diary, 40-49% | 7, 9 |
| Rabusin et al. 2021 | Y | Y | Y, increase resistance (5kg inc in backpack) | Y, pain response | 7, 2 X D | 15RM | NR | 3 (12) | 15 (180) | Alfredson ECCT heel drop protocol | Y, diary, 60-94% | 8, 10 |
| Solomons et al. 2020 | Y | NR | NR | Y, pain response | NR | NR | NR | NR | NR | Isometric, CONCT, ECCT, no details | Y, diary, 83-100% | 4, 6 |
| Ramon et al. 2020 | Y | NR | NR | NR | 7 | 10RM | NR | 1 | 10 | Gluteal EX: Bridging, hip abduction & extension | NR | 2, 2 |
| Scott et al. 2019 | Y | NR | NR | NR | 3 | NR | NR | NR | NR | HSRT (modified Kongsgaard protocol):no details | NR | 2, 2 |
| Stefansson et al. 2019 | Y | Y | Y, increase resistance (5kg inc in backpack) | Y, pain response | 7, 2 x D | 10-15RM | NR | 1-3 | 10-15 | Alfredson ECCT heel drop protocol | NR | 8, 8 |
| Boesen et al. 2017 | Y | Y | NR | Y, pain response | 7, 2 x D | 15RM | NR | 6 | 180 | Alfredson ECCT heel drop protocol | Y, diary, 70% | 6, 8 |
| Chesterton et al. 2021 | Y | NR | Y, increase difficulty | Y, pain response | NR | NR | NR | NR | NR | Progressive foot, calf and hip strength EX, no details | Y, diary | 5, 6 |
| Rasenberg et al. 2020 | Y | NR | NR | NR | NR | NR | NR | NR | NR | Rathleff heel-raise protocol, no details | Y, diary | 2, 3 |
| Johannsen et al. 2020 | Y | NR | NR | NR | 3 | NR | NR | NR | NR | Ankle inversion, first toe flexion, heel raises (performed slowly) | Y, diary, 100% | 2, 4 |
| Thong-On et al. 2019 | Y | Y | Y, increase resistance | Y, increase difficulty | 7 | 10-15RM | NR | 3 | 10-15 | Heel raises, toe curles, ankle inversion & eversion with resistance bands | Y, diary | 7, 9 |
| Cil et al. 2019 | Y | Y | Y, increase repetitions | NR | 7 | 10-15RM | NR | 3 | 10-15 | Strength EX; foot intrinsic, ankle & hip, TheraBand | NR | 5, 5 |
| Kamonseki et al. 2016 | Y | Y | Y, increase resistance | NR | 7 | 10-15RM | NR | 3 | 10-15 | Strength EX: Toe curl, short foot, inversion, eversion, PF, DF, hip External rotation & abduction | NR | 5, 5 |
| Brown et al. 2006 | NR | NR | NR | NR | NR | NR | NR | NR | NR | Alfredson protocol, no details | Y, verbal | 0, 1 |
| Niesen-Vertommen et al. 1992 | Y | Y | Y, increase resistance (10% of bodyweight) | Y, pain response | 6 | 10RM | NR | 5 | 10 | ECCT (stanish protocol) vs CONCT heel raises on a step | Y, diary | 8, 9 |
| Jensen et al. 1989 | Y | Y | Y, increase speed/velocity | Y, difficultly | 3 | Speed (30-70 degrees /s), 5RM | NR | 6-4 | 5 | ECCT: isokinetic dynamometer | Y, diary | 7, 8 |
| Yu et al. 2013 | Y | Y | Y, increase resistance (5-10lbs) | Y, pain response | 3 | NR | 50MIN | 3 | 15 | ECCT heel drop: modified Alfredson & Stanish protocols  CONCT heel riase: Mafi protocol | NR | 8, 8 |
| Wheeler et al. 2021 | Y | Y | Y, increase repetitions as able | Y, pain response | 7 | NR | NR | NR | NR | Isotonic hip strength EX: abduction, bridging clams | NR | 7, 7 |
| Zhang et al. 2013 | Y | Y | Y, increase resistance (5kg inc in backpack) | Y, pain response | 7 | 15RM | NR | 3 | 15 | Modified Alfredson heel drop protocol | NR | 8, 8 |
| Bell et al. 2013 | Y | NR | NR | Y, pain response | 7 | NR | NR | NR | 180 | Alfredson heel drop protocol, no details | Y, diary, 62-65% | 4, 6 |
| Pietrosimone et al. 2020 | Y | Y | NR | NR | Single session | 70% MVIC | NR | 5 | 45/s | Isometric knee extension | NR | 4, 4 |
| COHORT STUDIES |  |  |  |  |  |  |  |  |  |  |  |  |
| Sancho et al. 2019 | Y | Y | Y, increase resistance | Y, pain response | 7 (isometric) 3 (isotonic) | 8-25RM  80% OF 6RM for 8RM | NR | 2-3 | 8-25 | Seated & standing heel raises (isometric & isotonic), Hip abduction & extension, DL jumps, SL hops, running | Y, diary, 70% | 7, 9 |
| Croisier et al. 2001 | Y | Y | Y, increase resistance & speed | Y, pain response | 3 | 30-80% max intensity, 30-180 degrees/S velocity | NR | 1-5 | 20-30 | Isokinetic dynamometer: Heel raise (Achilles), knee extension (patellar) | Y, supervised | 7, 8 |
| Ohberg et al. 2004 | Y | Y | Y, increase resistance (backpack or weights) | Y, pain response | 7, 2 x D | 15RM | NR | 3 X 2 (6) | 15 X 3 X 2 (90) | Alfredson eccentric heel-drop protocol | NR | 7, 7 |
| Sayana et al. 2007 | Y | Y | Y, increase resistance (5kg inc in backpack) | Y, pain response | 7, 2 x D | 15RM | NR | 3 X 2 (6) | 15 X 3 X 2 (90) | Alfredson eccentric heel-drop protocol | Y, diary | 8, 9 |
| Abat et al. 2014 | Y | Y | NR | NR | 2 | 10RM | NR | 3 | 10 | Isoinertial eccentric training machine | Y, supervised | 4, 5 |
| Riel et al. 2019 | Y | Y | Y, increase resistance (backpack) & volume (sets) | Y, as many sets as possible, pain response | 4 | 8RM | 64 S/set | 8 | AMAP | HSRT heel-raise on a step, loaded backpack | Y, diary, 75% | 7, 9 |
| Kongsgaard et al 2010 | Y | Y | Y, increase resistance | Y, pain response | 3 | 6-15RM | NR | 4 | 6-15 | Knee extension, squat, leg press, hack squat. | NR | 7, 7 |
| Wetke et al. 2015 | Y | Y | Y, increase difficulty (no external load) | Y, pain response | 7 | 20RM | NR | 3 | 20 | Bodyweight DL heel raises progressing to SL & higher height | Y, verbal, 65% | 7, 9 |
| Maffulli et al. 2008 | Y | Y | Y, increase resistance & speed (5kg INC in backpack) | Y, pain response | 7, 2 x D | 10-15RM | NR | 1-3 | 10-15 | Modified Alfredson heel-drop protocol | Y, diary | 8, 9 |
| Shalabi et al. 2004 | Y | Y | Y, increase resistance (backpack) | Y, pain response | 7, 2 x D | 15RM | NR | 3 X 2 (6) X 2 (12) | 15 X 3 X 2 (90) X 2 (180) | Modified Alfredson heel-drop protocol | Y, diary | 7, 8 |
| Mansur et al. 2019 | Y | Y | Y, increase resistance (5kg INC in backpack) | Y, pain response | 7, 2 X D | 15RM | NR | 3 X 2 (6) X 2 (12) | 15 X 3 X 2 (90) X 2 (180) | Modified Alfredson heel-drop protocol | NR | 8, 8 |
| Abat et al. 2015 | Y | Y | NR | NR | 2 | 10RM | NR | 3 | 10 | Isoinertial ECCT, leg press machine | NR | 4, 4 |
| Alfredson et al. 1999 | Y | Y | Y, increase resistance (backpack then weight machine) | Y, pain response | 7, 2 X D | 15RM | NR | 3 X 2 (6) X 2 (12) | 15 X 3 X 2 (90) X 2 (180) | Modified Alfredson heel-drop protocol | NR | 7, 7 |
| O’Neill et al. 2019 | Y | Y | NR | NR | Single session | 70% MVIC | 45/S | 1 | 1 | Single session 45/s isometric contraction with isokinetic dynamometry | NR | 4, 4 |
| Ooi et al. 2019 | Y | NR | NR | NR | NR | NR | NR | NR | NR | Alfredson heel-drop protocol | NR | 2, 2 |
| Kaux et al. 2015 | Y | NR | NR | NR | 3 | NR | NR | NR | NR | Sub-maximal ECCT, no details | NR | 2, 2 |
| Alfredson et al. 2003 | Y | Y | Y, increase resistance (5kg INC in backpack) | Y, pain response | 7, 2 X D | 15RM | NR | 3 X 2 (6) X 2 (12) | 15 X 3 X 2 (90) X 2 (180) | Alfredson heel-drop protocol | NR | 8, 8 |
| De Jonge et al. 2015 | Y | NR | NR | NR | 7 | NR | NR | NR | 180 | Alfredson heel-drop protocol, no details | NR | 2, 2 |
| Panni et al. 2000 | Y | NR | NR | NR | NR | NR | NR | NR | NR | Eccentric and isometric exercises, no details. | NR | 2, 2 |
| Angermann et al. 1999 | Y | Y | Y, increase resistance & speed (5kg INC) | Y, pain response | 7, 2 X D | 10RM | NR | 3-8 | 10 | Isotonic heel raises, no details | Y, verbal | 8, 9 |
| Von Wehren et al. 2019 | Y | Y | NR | NR | 7, 2 X D | 15RM | NR | 3 X 2 (6) X 2 (12) | 15 X 3 X 2 (90) X 2 (180) | Alfredson heel-drop protocol | NR | 4, 4 |
| Kaux et al. 2014 | Y | Y | Y, increase difficultly/incline angle (bodyweight) | Y, pain response | 3 | 15-20RM | NR | 3-5 | 15-20 | Eccentric DSL squat, isometric EX | Y, diary, 75% | 7, 9 |
| Wei et al. 2017 | Y | Y | Y, increase resistance & speed (5kg INC) | Y, pain response | 7, 2 X D | 15RM | NR | 3 X 2 (6) X 2 (12) | 15 X 3 X 2 (90) X 2 (180) | Alfredson heel-drop protocol | Y, verbal | 8, 9 |
| Basas et al. 2018 | Y | Y | Y, increase resistance | Y, pain response | 3 | NR | NR | NR | NR | Eccentric + isometric exercises for quadriceps with ES. | NR | 7, 7 |
| Fahlstrom et al. 2003 | Y | Y | Y, increase resistance | Y, pain response | 7, 2 X D | 15RM | NR | 3 X 2 (6) X 2 (12) | 15 X 3 X 2 (90) X 2 (180) | Alfredson heel-drop protocol | NR | 7, 7 |
| Jonsson et al. 2008 | Y | Y | Y, increase resistance (backpack) | Y, pain response | 7, 2 X D | 15RM | NR | 3 X 2 (6) X 2 (12) | 15 X 3 X 2 (90) X 2 (180) | Modified Alfredson heel-drop protocol | Y, verbal | 7, 8 |
| Abate et al. 2020 | Y | Y | Y, increase resistance | Y, pain response | 7, 2 X D | 15RM | NR | 3 X 2 (6) X 2 (12) | 15 X 3 X 2 (90) X 2 (180) | Alfredson heel-drop protocol | Y, diary, 70% | 7, 9 |
| Wheeler et al. 2020 | Y | NR | NR | NR | NR | NR | NR | NR | NR | ECCT, no details | NR | 2, 2 |
| Lagas et al. 2021 | Y | NR | NR | NR | NR | NR | NR | NR | NR | Calf strengthening EX, no details. | NR | 2, 2 |
| Robinson et al. 2021 | Y | NR | NR | NR | 7 | NR | NR | 3 | 30 | Ecct: calf raises, no details | NR | 2, 2 |
| Wheeler et al. 2021 | Y | NR | NR | NR | 7 | NR | NR | NR | NR | IFM EX, Isotonic calf raises, no details | NR | 2, 2 |
| Mantovani et al. 2020 | Y | Y | Y, increase resistance | Y, RPE & pain response | Single session | RPE 6, 50% body weight load | NR | 5 | 45/s | Isometric heel raise | Y, sup | 7, 8 |
| CASE SERIES |  |  |  |  |  |  |  |  |  |  |  |  |
| Kulig et al. 2009 | Y | Y | Y, increase resistance (modified by therapist) | Y, individual resistance | 7, 2 x D | 15RM | NR | 3 | 15 | Eccentric tibialis posterior EX with TibPost loader device | Y, diary, 77-100% | 8, 10 |
| Deans et al. 2012 | Y | NR | NR | NR | NR | NR | NR | NR | NR | ECCT, no details | NR | 2, 2 |
| Pavone et al. 2016 | Y | NR | NR | NR | 4 | NR | NR | NR | NR | ECCT, no details | NR | 2, 2 |
| Romero-rodriguez et al. 2011 | Y | Y | Y, always maximal intensity | Y, always maximal intensity | 2 | 10RM | NR | 4 | 10 | Isoinertial flywheel ECCT, maximal effort | NR | 8, 10 |
| Wheeler et al. 2019 | Y | NR | NR | NR | NR | NR | NR | NR | NR | ECCT & isometric EX, no details | NR | 2, 2 |
| Syverston et al. 2017 | Y | Y | NR | NR | 7, 2 X D | 15RM | NR | 3 X 2 (6) X 2 (12) | 15 X 3 X 2 (90) X 2 (180) | Alfredson heel-drop protocol | NR | 4, 4 |
| Robinson et al. 2020 | Y | NR | NR | NR | NR | 30RM | NR | 3 | 30 | Short foot EX, resisted tibialis posterior EX, Calf raises. | NR | 2, 2 |
| Benito et al. 2016 | Y | Y | Y, increase resistance (2.5kg INC) | NR | 7 | 8-15RM | NR | 3 | 8-15 | Modified Alfredson heel-drop protocol | NR | 6, 6 |
| Silbernagel et al. 2011 | Y | Y | Y, increase resistance (external weights) & speed | Y, pain response | 3 | 15M | NR | 3 | 15 | ECCT heel raises, Plyometric EX | NR | 7, 7 |
| Morton et al. 2014 | Y | NR | NR | NR | NR | NR | NR | NR | NR | ECCT DSL squat, no details | NR | 2, 2 |
| Van ark et al. 2013 | Y | Y | Y, increase resistance & difficultly | Y, pain response | 7 | 8-10RM | NR | 3 | 8-20 | Isometric quadriceps, SLR, side-lying abduction, squats, calf raises, SL squat, DSL squat, lunges, step-downs, bridging, jumping EX | Y, diary | 7, 8 |
| Munoz Fernandez et al. 2021 | Y | Y | NR | Y, pain response | NR | NR | NR | 3 | 15 | Hip clams, single limb bridge, DSL squat, pelvic drop, hip abduction, squat, deadlift, short foot. | NR | 4, 4 |
| Skovlund et al. 2020 | Y | Y | Y, increase volume | Y, pain response | 3 | 10RM (30% OF 1RM) | NR | 6 | 5-30 | BFRT: SL leg press & knee extension | Y, diary, 98% | 7, 9 |
| Jayaseelan et al. 2017 | Y | NR | NR | NR | 7, 2 X D | 15RM | NR | 3 | 15 | Modified Alfredson heel-drop protocol | Y, verbal | 2, 3 |
| Bianco et al. 2019 | Y | NR | Y, increase speed | Y, pain response | NR | 10-15RM | NR | 2-3 | 10-15 | Wall decline squat, SL mini squat, squat, DSL squat, drop squat, SL squat, jump downs | NR | 5, 5 |
| CASE REPORT |  |  |  |  |  |  |  |  |  |  |  |  |
| Eckenrode et al. 2015 | Y | Y | Y, increase resistance & difficultly | Y, pain response | 7, 2 X D | 15RM | NR | 3 | 15 | Alfredson heel-drop protocol. Other EX; bridging, lying hip abduction, SLR, wall squat | NR | 7, 7 |
| Papa et al. 2012 | Y | Y | Y, increase resistance | NR | 5 | 10-15RM | NR | 3 | 10-15 | Modified Alfredson heel-drop protocol | NR | 5, 5 |
| Dos Santos et al. 2016 | Y | NR | NR | NR | NR | 10RM, with 3kg weight | NR | 3 | 10 | Hip EX: flexion, abduction, adduction, extension | NR | 2, 2 |
| Lee et al. 2019 | Y | NR | NR | NR | 3 | NR | 30min | NR | NR | Calf and hip strength EX, no details | NR | 2, 2 |
| Ross et al. 2017 | Y | Y | Y, increase resistance (backpack) | Y, pain response | NR | 8-15RM | NR | 3 | 8-15 | Isotonic heel raises, TheraBand PF & DF, SL balance EX, plyometric EX | Y, diary | 7, 8 |
| Cuddeford et al. 2020 | Y | Y | Y, increase resistance (10lbs INC) | Y, increase difficulty | 2 | 1RM (load), 15-30RM | 15MIN | 4 | 15-30 | BFRT: SL leg press, DSL squat | NR | 8, 8 |
| Krueger et al. 2020 | Y | Y | Y, increase resistance | Y, RPE for load & pain response | 3 | RPE 7-9 (RIR 3-1), 1RM (load), 6-15RM | NR | 3 | 6-15 | HSRT: back squat, deadlift, hip thrust, hamstring curl, SL: deadlift, reverse lunge, hamstring curl | Y, verbal | 7, 8 |
| Borda et al. 2017 | Y | NR | NR | NR | 7 | NR | NR | 2-3 | 15-20 | ECCT: heel drop: knee flexed and straight | NR | 2, 2 |
| Rauseo et al. 2017 | Y | Y | Y, increase resistance | Y, pain response | 7, 2 X D | 15RM | NR | 3 | 15 | ECCT: resisted hip flexion Other EX: bridging, squats, SL deadlift | Y, verbal | 7, 8 |
| McCormack et al. 2012 | Y | Y | Y, increase resistance | Y, pain response | 7, 2 x D | 10-20RM | NR | 2 | 10-20 | Modified Alfredson heel drop protocol | NR | 7, 7 |
| Patla et al. 2015 | Y | NR | Y, increase volume | NR | NR | 10-15RM | NR | 2-3 | 10-15 | Heel riase with ball squeeze, DL heel raise with SL lowering, pronation & supination | NR | 3, 3 |
| Pinkelman et al. 2012 | Y | Y | Y, increase velocity | Y, pain response | 14 sessions over 6 weeks | 15-30RM | NR | 3-5 | 15-30 | ECCT: manually resisted great toe extension | NR | 7, 7 |
| Francis et al. 2020 | Y | Y | Y, increase resistance (1.5kg INC in backpack) | NR | 7, 2 X D | 12RM | NR | 3 | 12 | Modified Alfredson heel-drop protocol, running program | Y, diary, 80% | 6, 8 |
| McCreesh et al. 2013 | Y | Y | Y, increase resistance (backpack) | NR | 7, 2 x D | 10RM | NR | 3 | 10 | Purdam ECCT protocol: DSL squat | NR | 5, 5 |
| Hensley et al. 2012 | Y | Y | Y, increase resistance (theraband) | NR | 3 | 15RM | NR | 3 | 15 | Peroneal strength EX: theraband resisted inversion & eversion, heel raises | NR | 5, 5 |
| Cushman et al. 2015 | Y | NR | NR | Y, pain response | 7 | 12-15RM | NR | 3 | 12-15 | ECCT: hip extension performed slowly on moving treadmill | NR | 4, 4 |
| Thompson et al. 2017 | Y | Y | Y, increase resistance (external load) | Y, load, RPE | 3 | % Of 6-8RM, 7-9/10 RPE | NR | 2-6 | 6-12 | DL squats, SL pelvic thrust, SL RDL, SL heel raises, band walks, SL lunge | NR | 7, 7 |
| Jayaseelan et al. 2014 | Y | Y | Y, increase resistance or volume | Y, technique & pain response | 7 | 10-15RM | NR | 3 | 10-15 | Leg curl machine, SL deadlift, bridge waslk-outs, side lying hip abduction | NR | 7, 7 |
| Dumont et al. 2006 | Y | Y | Y, increase speed & resistance (5% bodyweight INC) | Y, pain response | 7 | 10RM | NR | 3 | 10 | ECCT: drop squats | Y, diary | 8, 9 |
| Silva et al. 2015 | Y | Y | Y, increase resistance (2kg INC) | Y, pain response & technique | 3 | 50%1RM, 15RM | 30MIN | 3 | 15 | Prone hip extension, birddog, SL deadlift, drop jumps | NR | 8, 8 |
| McCormack et al. 2012 | Y | NR | NR | Y, increase difficulty | 2 | 10RM | NR | 2-3 | 10 | Prone hamstring curl, resisted hip extension, seated hamstring curl, good mornings, unilateral bridging, lunges, Nordics | NR | 4, 4 |
| Van Rooy et al. 2009 | Y | NR | NR | Y, pain response | 7 | 10RM | NR | 2 | 10 | ECCT hip abduction, lunges, bridging, | NR | 4, 4 |
| Greene et al. 2002 | Y | Y | Y, increase resistance (external weight) | NR | NR | 10-20RM | NR | 2-4 | 10-20 | Squats, leg pulls, heel raises | Y, diary | 6, 7 |
| Rowan et al. 2013 | Y | NR | NR | NR | 7 | 10RM | NR | 3 | 10 | ECCT: DSL squats | NR | 2, 2 |
| Goldman et al. 2010 | Y | Y | Y, increase resistance (external weight) | Y, pain response | 3 | 10-15RM, 70-100% of 1RM | NR | 3-5 | 10-15 | ECCT: DSL squats, leg press, knee extension, hamstring curl, step downs, heel taps | NR | 7, 7 |
| Cuddeford et al. 2018 | Y | Y | Y, increase resistance (external weight) | Y, difficultly | NR | 6-8RM, 150% BW + 40% INC | NR | 3 | 6-8 | ECCT heel drop with leg press machine | Y, diary | 7, 8 |
| OTHER |  |  |  |  |  |  |  |  |  |  |  |  |
| Longitudinal with control  Masood et al. 2014 | Y | Y | Y, increase resistance (2.5kg INC) | Y, pain response | 7, 2 x D | 15RM | NR | 3 X 2(6) | 15 X 3 X 2 (90) | Modified Alfredson heel-drop protocol | Y, diary, 81% | 8, 10 |
| Before-after design  Gardin et al. 2010 | Y | Y | NR | NR | 7, 2 X D | 15RM | NR | 3 X 2(6) X 2 (12) | 15 X 3 X 2 (90) X 2 (180) | Modified Alfredson heel-drop protocol | NR | 4, 4 |
| Case control  Langberg et al. 2007 | Y | Y | Y, increase resistance (BY 20% in backpack) | Y, pain response | 7, 2 X D | 15RM | NR | 3 X 2(6) X 2 (12) | 15 X 3 X 2 (90) X 2 (180) | Modified Alfredson heel-drop protocol | NR | 8, 10 |
| Quasi experimental (non-randomised)  Stasinopoulos et al. 2013 | Y | Y | Y, increase resistance & speed (backpack) | Y, pain response | 7, 2 X D | 15RM | NR | 3 X 2(6) X 2 (12) | 15 X 3 X 2 (90) X 2 (180) | Alfredson heel-drop protocol V Stanish protocol | Y, diary | 7, 8 |
| Quasi experimental (non-randomised)  Kanniappan et al. 2020 | Y | Y | NR | NR | 7, 2 X D | 15RM (ECCT) 45/s (iso) | NR | 3 | 15 (ecct) 5 (iso) | Modified Alfredson heel-drop protocol V Isometric EX (static plantflexion max contraction) | NR | 4, 4 |
| Quasi experimental (non-randomised)  Purdam et al. 2004 | Y | Y | Y, increase resistance (backpack) | Y, pain response | 7 | 15RM | NR | 3 | 15 | ECCT: DSL squat v standard flat squat | NR | 7, 7 |
| Quasi experimental (non-randomised  Van der Vlist et al. 2020 | Y | Y | Y, increase resistance (weight vest) | Y, RPE & pain response | Single session | Max intensity (30kg), RPE | 13MIN | 5 | 45/s | Isometric heel raises, seated & standing | Y, sup | 7, 8 |
| Before-after design  Morgan et al. 2018 | Y | Y | NR | Y, pain response & load tolerance | 3 | NR | 30MIN | NR | NR | ECCT, isometric EX, Hip & core strength, no details | NR | 6, 6 |
| Observational prospective clinical trial  De Vos et al. 2012 | Y | Y | Y, increase resistance (2KG INC in backpack) | Y, pain response | 7, 2 X D | 15RM | NR | 3 X 2(6) X 2 (12) | 15 X 3 X 2 (90) X 2 (180) | Modified Alfredson heel-drop protocol | NR | 8, 8 |
| Retrospective case control  Park et al. 2021 | Y | Y | Y, increase resistance (2KG INC in backpack) | Y, pain response | 7, 2 X D | 15RM | NR | 3 X 2 | 15 X 3 (45) | Modified Alfredson heel-drop protocol | NR | 8, 8 |
| Case control  Ram et al. 2013 | Y | Y | NR | Y, pain response | 7, 2 X D | 15RM | NR | 3 X 2(6) X 2 (12) | 15 X 3 X 2 (90) X 2 (180) | Alfredson heel-drop protocol | Y, diary, 17/20 | 6, 8 |
| Retrospective chart review  Vander Doelen et al. 2020 | Y | Y | Y, increase resistance | NR | 2-3 | 6-15RM (HSRT) 5 X 45/S @70% MVIC (ISO) | NR | 4 | 6-15 | Isometric (RIO protocol) HSRT: (kongsgaard protocol) leg press, squat, hack squat | NR | 5, 5 |

**Abbreviations:** ECCT: eccentric training, Y: yes, NR: not reported, D: day, RM: repetition maximum, KG: kilogram, INC: increment, MVIC: maximum voluntary isometric contraction, HSRT: heavy slow resiatnce training, RPE: rating of perceived exertion, MIN: minutes, EX: exercise. RIR: repetitions in reserve, RTP: resistance training principles, WK: week, PF: plantarflexion, DF: dorsiflexion, CONCT: concentric training; DSL: decline single leg

**APPENDIX 4: Table 6: Toigo and Boutellier framework exercise descriptors reporting for each study**

| Author | T1: load magnitude | T2: repetitions | T3: sets | T4: rest between sets | T5: sessions per d/wk. | T6: duration period | T7: contraction mode | T8: rest between reps | T9: tut | T10: muscular failure | T11:rom | T12: recovery between sessions | T13: anatomical exercise definition | TBF TOTAL/13 |
| --- | --- | --- | --- | --- | --- | --- | --- | --- | --- | --- | --- | --- | --- | --- |
|  |  |  |  |  |  |  |  |  |  |  |  |  |  |  |
| BEYER 2015 | Y | Y | Y | Y | Y | Y | Y | Y, NIL | Y | N | Y | Y | Y | 12 |
| KONGSGAARD 2009 | Y | Y | Y | Y | Y | Y | Y | Y, NIL | Y | N | Y | Y | Y | 12 |
| RIEL 2019 | Y | Y | Y | Y | Y | Y | Y | Y, NIL | Y | Y | Y | Y | Y | 13 |
| STEVENS 2014 | Y | Y | Y | Y | Y | Y | Y | Y, NIL | Y | Y | Y | Y | Y | 13 |
| CUNHA 2012 | Y | Y | Y | N | Y | Y | Y | Y, NIL | N | N | Y | Y | Y | 10 |
| KULIG 2009 | Y | Y | Y | Y | Y | Y | Y | Y, NIL | Y | N | Y | Y | Y | 12 |
| BAHR 2006 | Y | Y | Y | N | Y | Y | Y | Y, NIL | Y | N | Y | Y | Y | 11 |
| LEE 2020 | Y | Y | Y | N | Y | Y | Y | Y, NIL | Y | N | Y | Y | Y | 11 |
| FROHM 2007 | Y | Y | Y | Y | Y | Y | Y | Y, NIL | N | N | Y | Y | Y | 11 |
| SILBERNAGEL 2001 | Y | Y | Y | N | Y | Y | Y | Y, NIL | N | N | Y | Y | Y | 10 |
| BALIUS 2016 | N | Y | Y | N | Y | Y | Y | Y, NIL | N | N | N | Y | Y | 8 |
| MAFI 2001 | Y | Y | Y | N | Y | Y | Y | Y, NIL | N | N | Y | Y | Y | 10 |
| NORREGAARD 2007 | Y | Y | Y | N | Y | Y | Y | Y, NIL | N | N | Y | Y | Y | 10 |
| STASINOPOLOUS 2004 | Y | Y | Y | N | Y | Y | Y | Y, NIL | N | N | Y | Y | Y | 10 |
| DE VOS 2007 | Y | Y | Y | N | Y | Y | Y | Y, NIL | N | N | Y | Y | Y | 10 |
| JOHANNSEN 2018 | N | N | N | N | Y | Y | N | N | N | N | N | Y | N | 3 |
| MACDONALD 2019 | Y | Y | Y | N | Y | Y | Y | Y, NIL | N | N | Y | Y | Y | 10 |
| GATZ 2020 | Y | Y | Y | N | Y | Y | Y | Y, NIL | N | N | Y | Y | Y | 10 |
| GANDERTON 2018 | Y | Y | Y | N | Y | Y | Y | Y, NIL | N | N | Y | Y | Y | 10 |
| SILBERNAGEL 2007 | Y | Y | Y | N | Y | Y | Y | Y, NIL | N | N | Y | Y | Y | 10 |
| CLIFFORD 2019 | Y | Y | Y | Y | Y | Y | Y | Y, NIL | Y | N | Y | Y | Y | 12 |
| STERGIOULAS 2008 | Y | Y | Y | Y | Y | Y | Y | Y, NIL | N | N | Y | Y | Y | 11 |
| ROMPE 2008 | Y | Y | Y | Y | Y | Y | Y | Y, NIL | N | N | Y | Y | Y | 11 |
| VAN ARK 2016 | Y | Y | Y | Y | Y | Y | Y | Y, NIL | Y | N | Y | Y | Y | 12 |
| ROOS 2004 | Y | Y | Y | N | Y | Y | Y | Y, NIL | N | N | Y | Y | Y | 10 |
| CHESTER 2008 | Y | Y | Y | N | Y | Y | Y | Y, NIL | N | N | Y | Y | Y | 10 |
| ROMPE 2007 | Y | Y | Y | N | Y | Y | Y | Y, NIL | N | N | Y | Y | Y | 10 |
| THIJS 2017 | Y | Y | Y | N | Y | Y | Y | Y, NIL | N | N | Y | Y | Y | 10 |
| HORSTMANN 2013 | Y | Y | Y | N | Y | Y | Y | Y, NIL | Y | N | Y | Y | Y | 11 |
| ALFREDSON 1998 | Y | Y | Y | N | Y | Y | Y | Y, NIL | N | N | Y | Y | Y | 10 |
| ALVAREZ 2006 | Y | Y | Y | N | Y | Y | Y | Y, NIL | N | N | Y | Y | Y | 10 |
| KEARNEY 2013 | Y | Y | Y | N | Y | Y | Y | Y, NIL | N | N | Y | Y | Y | 10 |
| TUMILTY 2012 | Y | Y | Y | N | Y | Y | Y | Y, NIL | N | N | Y | Y | Y | 10 |
| YELLAND 2011 | Y | Y | Y | N | Y | Y | Y | Y, NIL | N | N | Y | Y | Y | 10 |
| MCCORMACK 2016 | Y | Y | Y | N | Y | Y | Y | Y, NIL | N | N | Y | Y | Y | 10 |
| TUMILTY 2016 | Y | Y | Y | N | Y | Y | Y | Y, NIL | N | N | Y | Y | Y | 10 |
| CANNELL 2001 | Y | Y | Y | N | Y | Y | Y | Y, NIL | Y | N | Y | Y | Y | 11 |
| JONSSON 2005 | Y | Y | Y | N | Y | Y | Y | Y, NIL | N | N | Y | Y | Y | 10 |
| MELLOR 2018 | Y | Y | Y | N | Y | Y | Y | Y, NIL | Y | N | Y | Y | Y | 11 |
| KEDIA 2014 | Y | Y | Y | N | Y | Y | Y | Y, NIL | N | N | Y | Y | Y | 10 |
| HERRINGTON 2007 | Y | Y | Y | N | Y | Y | Y | Y, NIL | N | N | Y | Y | Y | 10 |
| HOUCK 2015 | Y | Y | Y | N | Y | Y | Y | Y, NIL | Y | N | Y | Y | Y | 11 |
| DIMITRIOS 2012 | Y | Y | Y | Y | Y | Y | Y | Y, NIL | N | N | Y | Y | Y | 11 |
| PETERSEN 2007 | Y | Y | Y | N | Y | Y | Y | Y, NIL | N | N | Y | Y | Y | 10 |
| STEUNEBRINK 2013 | Y | Y | Y | N | Y | Y | Y | Y, NIL | N | N | Y | Y | Y | 10 |
| ROMPE 2009 | Y | Y | Y | Y | Y | Y | Y | Y, NIL | N | N | Y | Y | Y | 11 |
| YOUNG 2005 | Y | Y | Y | N | Y | Y | Y | Y, NIL | N | N | Y | Y | Y | 10 |
| DE JONGE 2010 | Y | Y | Y | N | Y | Y | Y | Y, NIL | N | N | Y | Y | Y | 10 |
| PRAET 2019 | Y | Y | Y | N | Y | Y | Y | Y, NIL | N | N | Y | Y | Y | 10 |
| RATHLEFF 2015 | Y | Y | Y | N | Y | Y | Y | Y, NIL | Y | N | Y | Y | Y | 11 |
| KNOBLOCH 2008 | Y | Y | Y | N | Y | Y | Y | Y, NIL | N | N | Y | Y | Y | 10 |
| WHEELER 2017 | N | N | N | N | N | N | N | N | N | N | N | N | N | 0 |
| CHOUDHARY 2021 | Y | Y | Y | N | Y | Y | Y | Y,NIL | N | N | N | Y | N | 8 |
| COWAN 2021 | Y | Y | Y | N | Y | Y | Y | Y,NIL | N | N | Y | Y | Y | 10 |
| HABETS 2021 | Y | Y | Y | N | Y | Y | Y | Y,NIL | N | N | Y | Y | Y | 10 |
| RUFFINO 2021 | Y | Y | Y | Y | Y | Y | Y | Y,NIL | Y | Y | Y | Y | Y | 13 |
| OLESEN 2021 | Y | Y | Y | N | Y | Y | Y | Y,NIL | N | N | Y | Y | Y | 10 |
| HASANI 2021 | Y | Y | Y | Y | Y | Y | Y | Y,NIL | Y | Y | Y | Y | Y | 13 |
| MANSUR 2021 | Y | Y | Y | N | Y | Y | Y | Y,NIL | N | N | Y | Y | Y | 10 |
| SPRAGUE 2021 | Y | Y | Y | Y | Y | Y | Y | Y,NIL | Y | Y | Y | Y | Y | 13 |
| AGERGAARD 2021 | Y | Y | Y | Y | Y | Y | Y | Y,NIL | Y | Y | Y | Y | Y | 13 |
| LOPEZ-ROYO 2021 | Y | Y | Y | N | Y | Y | Y | Y,NIL | N | N | Y | Y | Y | 10 |
| ABDELKADER 2021 | Y | Y | Y | Y | Y | Y | Y | Y,NIL | N | N | Y | Y | Y | 11 |
| VAN DER VLIST 2020 | Y | Y | Y | Y | Y | Y | Y | Y,NIL | Y | N | Y | Y | Y | 12 |
| BREDA 2020 | Y | Y | Y | N | Y | Y | Y | Y,NIL | N | N | Y | Y | Y | 10 |
| RABUSIN 2021 | Y | Y | Y | N | Y | Y | Y | Y,NIL | N | N | Y | Y | Y | 10 |
| SOLOMONS 2020 | N | N | N | N | N | N | Y | N | N | N | N | N | N | 1 |
| RAMON 2020 | Y | Y | Y | N | Y | Y | Y | Y,NIL | N | N | Y | Y | Y | 10 |
| SCOTT 2019 | N | N | N | N | N | N | Y | N | N | N | N | N | N | 1 |
| STEFANSSON 2019 | Y | Y | Y | N | Y | Y | Y | Y,NIL | N | N | Y | Y | Y | 10 |
| BOESEN 2017 | Y | Y | Y | N | Y | Y | Y | Y,NIL | N | N | Y | Y | Y | 10 |
| CHESTERTON 2021 | N | N | N | N | N | N | N | Y,NIL | N | N | N | N | Y | 2 |
| RASENBERG 2020 | N | N | N | N | N | N | Y | N | N | N | N | N | N | 1 |
| JOHANNSEN 2020 | N | N | N | N | Y | Y | Y | N | N | N | N | Y | N | 4 |
| THONG-ON 2019 | Y | Y | Y | Y | Y | Y | Y | Y,NIL | N | N | Y | Y | Y | 10 |
| CIL 2019 | Y | Y | Y | N | Y | Y | Y | Y,NIL | N | N | Y | Y | N | 9 |
| KAMONSEKI 2016 | Y | Y | Y | N | Y | Y | Y | Y,NIL | N | N | Y | Y | Y | 10 |
| BROWN 2006 | N | N | N | N | N | N | Y | N | N | N | N | N | N | 1 |
| NIESEN-VERTOMMEN | Y | Y | Y | N | Y | Y | Y | Y,NIL | N | N | Y | Y | Y | 10 |
| JENSEN 1989 | Y | Y | Y | N | Y | Y | Y | Y,NIL | N | Y | Y | Y | Y | 11 |
| YU 2013 | Y | Y | Y | N | Y | Y | Y | Y,NIL | N | N | Y | Y | Y | 10 |
| WHEELER 2021 | N | N | N | N | Y | Y | Y | Y,NIL | N | N | Y | Y | Y | 7 |
| ZHANG 2013 | Y | Y | Y | N | Y | Y | Y | Y,NIL | N | N | Y | Y | Y | 10 |
| BELL 2013 | Y | Y | N | N | Y | Y | Y | Y,NIL | N | N | N | Y | N | 7 |
| PIETROSIMONE | Y | Y | Y | Y | Y | Y | Y | Y | Y | N | Y | Y | Y | 12 |
| DE JONGE 2011 | Y | Y | N | N | N | Y | Y | Y,NIL | N | N | N | Y | N | 6 |
| DE VOS 2010 | Y | Y | N | N | N | Y | Y | Y,NIL | N | N | N | Y | N | 6 |
| WARDEN 2008 | Y | Y | Y | N | Y | Y | Y | Y,NIL | N | N | Y | Y | Y | 10 |
| VISNES 2005 | Y | Y | Y | N | Y | Y | Y | Y,NIL | N | N | Y | Y | Y | 10 |
| VAN ARK 2018 | Y | Y | Y | Y | Y | Y | Y | Y,NIL | Y | N | Y | Y | Y | 12 |
| THOMPSON 2019 | Y | Y | Y | N | Y | N | Y | Y,NIL | N | N | N | Y | N | 6 |
| CACCHIO 2011 | Y | Y | Y | N | Y | Y | Y | Y,NIL | N | N | N | Y | N | 8 |
| MUNTEANU 2014 | Y | Y | Y | N | Y | Y | Y | Y,NIL | N | N | Y | Y | Y | 10 |
| VAN DER WORP 2014 | Y | Y | Y | N | Y | Y | Y | Y,NIL | N | N | Y | Y | N | 9 |
| ROMER-MORALES 2018 | Y | Y | Y | N | Y | Y | Y | Y,NIL | N | N | Y | Y | Y | 10 |
| ROMERO-MORALES 2020 | Y | Y | Y | N | Y | Y | Y | Y,NIL | N | N | Y | Y | Y | 10 |
| RYAN 2014 | Y | Y | Y | N | Y | Y | N | Y,NIL | N | N | N | Y | N | 6 |
| RIEL 2018 | Y | Y | Y | Y | Y | Y | Y | Y,NIL | Y | Y | Y | Y | Y | 13 |
| KOSZALINSKI 2020 | Y | Y | Y | N | N | Y | Y | Y,NIL | N | N | N | N | Y | 7 |
| PEARSON 2012 | N | N | N | N | N | Y | N | N | N | N | N | N | N | 1 |
| WANG 2007 | N | N | N | N | N | N | Y | N | N | N | N | N | N | 1 |
| NOTARNICOLA 2013 | N | Y | Y | N | N | N | Y | N | N | N | N | N | N | 3 |
| DRAGOO 2014 | N | N | N | N | N | N | Y | N | N | N | N | N | N | 1 |
| KAUX 2019 | Y | Y | Y | Y | Y | Y | Y | Y,NIL | N | N | Y | Y | Y | 11 |
| ABAT 2016 | Y | Y | Y | Y | N | Y | Y | Y,NIL | N | N | Y | N | Y | 9 |
| BIERNAT 2014 | Y | Y | Y | Y | N | Y | Y | Y,NIL | N | N | Y | Y | Y | 10 |
| RIO 2015 | Y | Y | Y | Y | Y | Y | Y | Y,NIL | Y | N | Y | Y | Y | 12 |
| HOLDEN 2020 | Y | Y | Y | Y | Y | Y | Y | Y,NIL | Y | N | Y | Y | Y | 12 |
| RIO 2017 | Y | Y | Y | Y | Y | Y | Y | Y,NIL | Y | N | Y | Y | Y | 12 |
| SANCHO 2019 | Y | Y | Y | N | Y | Y | Y | Y,NIL | N | N | Y | Y | Y | 10 |
| CROISIER 2001 | Y | Y | Y | N | Y | Y | Y | Y,NIL | N | N | Y | Y | Y | 10 |
| OHBERG 2004 | Y | Y | Y | N | Y | Y | Y | Y,NIL | N | N | Y | Y | Y | 10 |
| SAYANA 2007 | Y | Y | Y | Y | Y | Y | Y | Y,NIL | N | N | Y | Y | Y | 11 |
| ABAT 2014 | Y | Y | Y | N | Y | Y | Y | Y,NIL | N | N | Y | Y | Y | 10 |
| RIEL 2019 | Y | Y | Y | Y | Y | Y | Y | Y,NIL | Y | Y | Y | Y | Y | 13 |
| KONGSGAARD 2010 | Y | Y | Y | Y | Y | Y | Y | Y,NIL | Y | N | Y | Y | Y | 12 |
| WETKE 2015 | Y | Y | Y | N | Y | Y | Y | Y,NIL | N | N | Y | Y | Y | 10 |
| MAFFULLI 2008 | Y | Y | Y | Y | Y | Y | Y | Y,NIL | N | N | Y | Y | Y | 11 |
| SHALABI 2004 | Y | Y | Y | N | Y | Y | Y | Y,NIL | N | N | Y | Y | Y | 10 |
| MANSUR 2019 | Y | Y | Y | N | Y | Y | Y | Y,NIL | N | N | Y | Y | Y | 10 |
| ABAT 2015 | Y | Y | Y | N | Y | Y | Y | Y,NIL | N | N | Y | Y | Y | 10 |
| ALFREDSON 1999 | Y | Y | Y | N | Y | Y | Y | Y,NIL | N | N | Y | Y | Y | 10 |
| ALFREDSON 2003 | Y | Y | Y | N | Y | Y | Y | Y,NIL | Y | Y | Y | Y | Y | 10 |
| O'NEILL 2019 | Y | Y | Y | Y | Y | Y | Y | Y,NIL | N | N | Y | Y | Y | 10 |
| OOI 2019 | N | N | N | N | N | N | Y | N | N | N | N | N | N | 1 |
| KAUX 2015 | N | N | N | N | N | Y | Y | N | N | N | N | Y | N | 3 |
| DE JONGE 2015 | N | N | N | N | N | Y | Y | N | N | N | N | Y | N | 3 |
| PANNI 2000 | N | N | N | N | N | N | Y | N | N | N | N | N | N | 1 |
| ANGERMANN 1999 | Y | Y | Y | N | Y | Y | Y | Y,NIL | N | N | Y | Y | Y | 10 |
| VON WEHREN 2019 | Y | Y | Y | N | Y | Y | Y | Y,NIL | N | N | Y | Y | Y | 10 |
| KAUX 2014 | Y | Y | Y | Y | Y | Y | Y | Y,NIL | N | N | Y | Y | Y | 11 |
| WEI 2017 | Y | Y | Y | N | Y | Y | Y | Y,NIL | N | N | Y | Y | Y | 10 |
| BASAS 2018 | Y | N | N | N | Y | Y | Y | Y,NIL | N | N | Y | Y | Y | 8 |
| FAHLSTROM 2007 | Y | Y | Y | N | Y | Y | Y | Y,NIL | N | N | Y | Y | Y | 10 |
| JONSSON 2008 | Y | Y | Y | N | Y | Y | Y | Y,NIL | N | N | Y | Y | Y | 10 |
| ABATE 2020 | Y | Y | Y | N | Y | Y | Y | Y,NIL | N | N | Y | Y | Y | 10 |
| WHEELER 2020 | N | N | N | N | N | N | Y | N | N | N | N | N | N | 1 |
| LAGAS 2021 | N | N | N | N | N | N | Y | N | N | N | N | N | N | 1 |
| ROBINSON 2021 | Y | Y | Y | N | Y | Y | Y | Y,NIL | N | N | N | Y | N | 8 |
| WHEELER 2021 | N | N | N | N | N | N | Y | N | N | N | N | Y | N | 2 |
| MANTOVANI 2020 | Y | Y | Y | Y | Y | Y | Y | Y,NIL | Y | N | Y | Y | Y | 12 |
| KULIG 2009 | Y | Y | Y | N | Y | Y | Y | Y,NIL | N | Y | Y | Y | Y | 11 |
| DEANS 2012 | N | N | N | N | N | N | Y | N | N | N | N | N | N | 1 |
| PAVONE 2016 | N | N | N | N | N | N | Y | N | N | N | N | N | N | 1 |
| ROMERO-RODRIGUEZ 2011 | Y | Y | Y | Y | Y | Y | Y | Y,NIL | N | Y | Y | Y | Y | 12 |
| WHEELER 2019 | N | N | N | N | N | N | Y | N | N | N | N | N | N | 1 |
| SYVERSTON 2017 | Y | Y | Y | N | Y | Y | Y | Y,NIL | N | N | Y | Y | Y | 10 |
| ROBINSON 2020 | Y | Y | Y | N | Y | N | Y | Y,NIL | N | N | Y | N | Y | 8 |
| BENITO 2016 | Y | Y | Y | N | Y | Y | Y | Y,NIL | N | N | Y | Y | Y | 10 |
| SILBERNAGEL 2011 | Y | Y | Y | N | Y | Y | Y | Y,NIL | N | N | Y | Y | Y | 10 |
| MORTON 2014 | N | N | N | N | N | N | Y | N | N | N | N | N | N | 1 |
| MASOOD 2014 | Y | Y | Y | N | Y | Y | Y | Y,NIL | N | N | Y | Y | Y | 10 |
| VAN ARK 2013 | Y | Y | Y | Y | Y | Y | Y | Y,NIL | N | N | Y | Y | Y | 11 |
| MUNOZ FERNANDEZ 2021 | Y | Y | Y | Y | Y | Y | Y | Y,NIL | N | N | Y | N | Y | 10 |
| SKOVLUND 2020 | Y | Y | Y | Y | Y | Y | Y | Y,NIL | Y | Y | Y | Y | Y | 13 |
| JAYASEELAN 2017 | Y | Y | Y | N | Y | Y | Y | Y,NIL | N | N | Y | Y | Y | 10 |
| BIANCO 2019 | Y | Y | Y | N | N | Y | Y | Y,NIL | N | N | Y | N | Y | 8 |
| ECKENRODE 2015 | Y | Y | Y | Y | Y | Y | Y | Y,NIL | Y | N | Y | Y | Y | 12 |
| PAPA 2012 | Y | Y | Y | N | Y | Y | Y | Y,NIL | N | N | Y | Y | Y | 10 |
| DOSS ANTOS 2016 | Y | Y | Y | N | N | Y | N | N | N | N | N | N | Y | 5 |
| LEE 2019 | N | N | N | N | N | N | Y | N | N | N | N | N | N | 1 |
| ROSS 2017 | Y | Y | Y | N | Y | Y | Y | Y,NIL | N | N | Y | Y | Y | 10 |
| CUDDEFORD 2020 | Y | Y | Y | Y | Y | Y | Y | Y,NIL | N | Y | Y | Y | Y | 12 |
| KRUEGER 2020 | Y | Y | Y | N | Y | Y | Y | Y,NIL | Y | N | Y | Y | Y | 11 |
| BORDA 2017 | Y | Y | Y | N | Y | Y | Y | Y,NIL | N | N | Y | Y | Y | 10 |
| RAUSEO 2017 | Y | Y | Y | N | Y | Y | Y | Y,NIL | N | N | Y | Y | Y | 10 |
| MCCORMACK 2012 | Y | Y | Y | N | Y | Y | Y | Y,NIL | N | N | Y | Y | Y | 10 |
| PATLA 2015 | Y | Y | Y | N | N | Y | Y | Y,NIL | N | N | Y | N | Y | 8 |
| PINKELMAN 2012 | Y | Y | Y | N | N | Y | Y | Y,NIL | N | N | Y | N | Y | 8 |
| FRANCIS 2020 | Y | Y | Y | N | Y | Y | Y | Y,NIL | N | N | Y | Y | Y | 10 |
| MCCREESH 2013 | Y | Y | Y | N | Y | Y | Y | Y,NIL | N | N | Y | Y | Y | 10 |
| HENSLEY 2012 | Y | Y | Y | N | Y | Y | Y | Y,NIL | N | N | Y | Y | Y | 10 |
| CUSHMAN 2015 | Y | Y | Y | N | Y | Y | Y | Y,NIL | N | N | Y | Y | Y | 10 |
| THOMPSON 2017 | Y | Y | Y | Y | Y | Y | Y | Y,NIL | N | N | Y | Y | Y | 11 |
| JAYASEELAN 2014 | Y | Y | Y | N | Y | Y | Y | Y,NIL | N | N | Y | Y | Y | 10 |
| DUMONT 2006 | Y | Y | Y | N | Y | Y | Y | Y,NIL | N | N | Y | Y | Y | 10 |
| SILVA 2015 | Y | Y | Y | Y | Y | Y | Y | Y,NIL | N | N | Y | Y | Y | 11 |
| MCCORMACK 2012 | Y | Y | Y | N | Y | Y | Y | Y,NIL | N | N | Y | Y | Y | 10 |
| VAN ROOY 2009 | Y | Y | Y | N | Y | Y | Y | Y,NIL | N | N | Y | Y | Y | 10 |
| GREENE 2002 | Y | Y | Y | N | N | Y | Y | Y,NIL | N | N | Y | N | Y | 8 |
| ROWAN 2013 | Y | Y | Y | N | Y | Y | Y | Y,NIL | N | N | N | Y | N | 8 |
| GOLDMAN 2010 | Y | Y | Y | N | Y | Y | Y | Y,NIL | N | Y | Y | Y | Y | 11 |
| CUDDEFORD 2018 | Y | Y | Y | N | N | Y | Y | Y,NIL | N | N | Y | N | Y | 8 |
| GARDIN 2010 | Y | Y | Y | N | Y | Y | Y | Y,NIL | N | N | Y | Y | Y | 10 |
| LANGBERG 2007 | Y | Y | Y | N | Y | Y | Y | Y,NIL | N | N | Y | Y | Y | 10 |
| STASINOPOULOS 2013 | Y | Y | Y | Y | Y | Y | Y | Y,NIL | N | N | Y | Y | Y | 11 |
| KANNIAPPAN 2020 | Y | Y | Y | Y | Y | Y | Y | Y,NIL | N | N | Y | Y | Y | 11 |
| PURDAM 2004 | Y | Y | Y | N | Y | Y | Y | Y,NIL | N | N | Y | Y | Y | 10 |
| VAN DER VLIST 2020 | Y | Y | Y | Y | Y | Y | Y | Y | Y | N | Y | Y | Y | 12 |
| MORGAN 2018 | N | N | N | N | Y | Y | Y | N | N | N | N | Y | N | 4 |
| DE VOS 2012 | Y | Y | Y | N | Y | Y | Y | Y,NIL | N | N | Y | Y | Y | 11 |
| PARK 2021 | Y | Y | Y | Y | Y | Y | Y | Y | N | N | Y | Y | Y | 11 |
| RAM 2013 | Y | Y | Y | N | Y | Y | Y | Y,NIL | N | N | Y | Y | Y | 10 |
| VANDER DOELEN 2020 | Y | Y | Y | N | Y | Y | Y | Y | Y | N | Y | Y | Y | 11 |

**Abbreviations:** Y: yes, N: no, TBF: Toigo and Boutellier framework.

**APPENDIX 5: Table 7: Consensus on Exercise Reporting Template (CERT) items reporting for each study**

| C1: equipment | C2: instructor | C3: individual/group | C4: un/supervised | C5: adherence measure & reported | C6: motivation | C7a: progression rules | C7b: progressed how | C8: exercise details replication | C9: describe home program | C10: nonexercise components | C11: adverse events | C12: exercise setting | C13: exercise intervention details | C14a: generic or tailored | C14b: tailored how | C15: describe starting level | C16a: fidelity measured | C16b: exercise delivered as planned | CERT TOTAL/19 | Author |
| --- | --- | --- | --- | --- | --- | --- | --- | --- | --- | --- | --- | --- | --- | --- | --- | --- | --- | --- | --- | --- |
|  |  |  |  |  |  |  |  |  |  |  |  |  |  |  |  |  |  |  |  |  |
| Y | Y, PT | Y, I | Y, UN | Y | N | Y | Y | Y | Y | Y. EX ONLY | N | Y | Y | Y, I | PAIN | Y | Y | Y | 17 | BEYER 2015 |
| Y | Y | Y, I | Y, both | Y | N | Y | Y | Y | Y, NA | Y, EX ONLY | Y | Y | Y | Y, G | N | Y | Y | Y | 17 | KONGSGAARD 2009 |
| Y | Y | Y, I | Y, UN | Y | N | Y | Y | Y | Y | Y, EX ONLY | Y | Y | Y | Y, I | AMAP | Y | N | Y | 17 | RIEL 2019 |
| Y | Y | Y, I | Y, UN | Y | N | Y | Y | Y | Y | Y, EX ONLY | Y | Y | Y | Y,I | AMAP | Y | Y | Y | 18 | STEVENS 2014 |
| Y | Y, PT | Y, I | Y,SUP | N | N | Y | Y | Y | Y,NA | Y, EX ONLY | N | Y | Y | Y,I | PAIN | Y | N | N | 14 | CUNHA 2012 |
| Y | Y,PT | Y, I | Y,both | Y | N | Y | Y | Y | Y | Y, ORTHOSES | N | Y | Y | Y | IRAA | Y | Y | Y | 17 | KULIG 2009 |
| Y | Y,PT | Y, I | Y,UN | N | N | Y | Y | Y | Y | Y,EX ONLY | N | Y | Y | Y,I | PAIN | Y | N | N | 14 | BAHR 2006 |
| Y | N | Y, I | Y,UN | Y | N | Y | Y | Y | Y | Y,ESWT | N | Y | Y | Y,I | PAIN | Y | N | N | 14 | LEE 2020 |
| Y | Y | Y, I | Y,SUP | N | N | Y | Y | Y | Y,NA | Y,EX ONLY | N | Y | Y | Y,I | PAIN | Y | N | N | 14 | FROHM 2007 |
| Y | Y | Y, I | Y,SUP | Y | N | Y | Y | Y | Y,NA | Y | N | Y | Y | Y,I | PAIN | Y | N | N | 15 | SILBERNAGEL 2001 |
| N | Y | Y, I | Y,SUP | Y | N | N | N | Y | Y,NA | Y,SUPP | N | N | Y | Y,G | N | N | N | Y | 10 | BALIUS 2016 |
| Y | Y | Y, I | Y,UN | N | N | Y | Y | Y | Y | Y,NA | N | Y | Y | Y | PAIN | Y | N | Y | 15 | MAFI 2001 |
| Y | Y | Y, I | Y,UN | Y | N | Y | Y | Y | Y | Y,NA | N | Y | Y | Y | PAIN | Y | N | N | 15 | NORREGAARD 2007 |
| Y | Y | Y, I | Y,UN | N | N | Y | Y | Y | Y | Y,STRETCH | N | Y | Y | Y,I | PAIN | Y | N | N | 14 | STASINOPOLOUS 2004 |
| Y | Y | Y, I | Y,UN | Y | N | Y | Y | Y | Y | Y,SPLINT | N | Y | Y | Y,I | PAIN | Y | N | Y | 16 | DE VOS 2007 |
| N | Y | Y, I | Y,UN | Y | N | N | N | N | N | Y,CSI | N | N | N | N | N | N | N | N | 5 | JOHANNSEN 2018 |
| Y | Y,PT | Y, I | Y,SUP | Y | N | Y | Y | Y | Y,NA | Y,HIP EX | N | Y | Y | Y,I | PAIN | Y | N | Y | 16 | MACDONALD 2019 |
| Y | N | Y, I | Y,UN | Y | N | Y | Y | Y | Y,NA | Y,EX ONLY | Y | Y | Y | Y,I | PAIN | Y | N | N | 15 | GATZ 2020 |
| Y | Y,PT | Y, I | Y,UN | Y | N | Y | Y | Y | Y | Y,EDUCATION | Y | Y | Y | Y,I | ABILITY | Y | N | Y | 17 | GANDERTON 2018 |
| Y | Y,PT | Y, I | Y,UN | Y | N | Y | Y | Y | Y | Y,EX ONLY | Y | Y | Y | Y,I | PAIN | Y | N | N | 16 | SILBERNAGEL 2007 |
| Y | Y,PT | Y, I | Y,both | Y | N | Y | Y | Y | Y | Y,EDUCATION | Y | Y | Y | Y,I | PAIN | Y | Y | Y | 18 | CLIFFORD 2019 |
| Y | Y,PT | Y, I | Y,SUP | Y | N | Y | Y | Y | Y | Y,LLLT | N | Y | Y | Y,I | PAIN | Y | N | Y | 16 | STERGIOULAS 2008 |
| Y | Y | Y, I | Y,UN | Y | N | Y | Y | Y | Y | Y,EX ONLY | Y | Y | Y | Y,I | PAIN | Y | N | Y | 17 | ROMPE 2008 |
| Y | Y | Y, I | Y,UN | Y | N | Y | Y | Y | Y | Y,EX ONLY | N | Y | Y | Y,I | PAIN,TECHNIQUE | Y | N | Y | 16 | VAN ARK 2016 |
| Y | Y | Y, I | Y,UN | Y | N | Y | Y | Y | Y | Y,SPLINT | N | Y | Y | Y,I | PAIN | Y | N | Y | 16 | ROOS 2004 |
| Y | Y | Y, I | Y,UN | N | N | Y | Y | Y | Y | Y,ULTRASOUND | Y | Y | Y | Y,I | PAIN | Y | N | N | 15 | CHESTER 2008 |
| Y | Y | Y, I | Y,UN | Y | N | Y | Y | Y | Y | Y,EX ONLY | Y | Y | Y | Y,I | PAIN | Y | N | N | 16 | ROMPE 2007 |
| Y | Y | Y, I | Y,UN | Y | N | Y | Y | Y | Y | Y,ESWT | Y | Y | Y | Y,I | PAIN | Y | N | N | 16 | THIJS 2017 |
| Y | Y | Y, I | Y,SUP | Y | N | Y | Y | Y | Y | Y,EX ONLY | N | Y | Y | Y,I | FATIGUE | Y | N | N | 15 | HORSTMANN 2013 |
| Y | Y | Y, I | Y,UN | N | N | Y | Y | Y | Y | Y,EX ONLY | N | Y | Y | Y,I | PAIN | Y | N | N | 14 | ALFREDSON 1998 |
| Y | Y | Y, I | Y,UN | Y | N | Y | Y | Y | Y | Y,ORTHOSES | Y | Y | Y | Y,I | PAIN,TECHNIQUE | Y | N | Y | 17 | ALVAREZ 2006 |
| Y | Y | Y, I | Y,UN | N | N | Y | Y | Y | Y | Y,EX ONLY | Y | Y | Y | Y,I | PAIN | Y | N | N | 15 | KEARNEY 2013 |
| Y | Y | Y, I | Y,UN | Y | N | Y | Y | Y | Y | Y,LLLT | Y | Y | Y | Y,I | PAIN | Y | N | Y | 17 | TUMILTY 2012 |
| Y | Y | Y, I | Y,UN | Y | N | Y | Y | Y | Y | Y,PROLOTHERAPY | Y | Y | Y | Y,I | PAIN | Y | N | Y | 17 | YELLAND 2011 |
| Y | Y | Y, I | Y,both | N | N | Y | Y | Y | Y | Y,ASTYM | Y | Y | Y | Y,G | N | Y | N | Y | 15 | MCCORMACK 2016 |
| Y | Y | Y, I | Y,UN | Y | N | Y | Y | Y | Y | Y,LLLT | Y | Y | Y | Y,I | PAIN | Y | N | Y | 17 | TUMILTY 2016 |
| Y | Y | Y, I | Y,BOTH | N | N | Y | Y | Y | Y | Y,EX ONLY | N | Y | Y | Y,I | PAIN | Y | N | N | 14 | CANNELL 2001 |
| Y | Y | Y, I | Y,both | N | N | Y | Y | Y | Y | Y,EX ONLY | Y | Y | Y | Y,I | PAIN | Y | N | N | 15 | JONSSON 2005 |
| Y | Y | Y, I | Y,both | Y | N | Y | Y | Y | Y | Y,EDUCATION | Y | Y | Y | Y,I | PAIN,BORG | Y | Y | Y | 18 | MELLOR 2018 |
| Y | Y | Y, I | Y,UN | Y | N | Y | Y | Y | Y | Y,CON RX | N | Y | Y | Y,I | DIFFICULTY | Y | N | N | 15 | KEDIA 2014 |
| Y | Y | Y, I | Y,UN | Y | N | Y | Y | Y | Y | Y,EX ONLY | N | Y | Y | Y,I | PAIN | Y | N | Y | 16 | HERRINGTON 2007 |
| Y | Y | Y, I | Y,UN | Y | N | Y | Y | Y | Y | Y,ORTHOSES | Y | Y | Y | Y,I | PAIN,TECHNIQUE | Y | N | Y | 17 | HOUCK 2015 |
| Y | Y | Y, I | Y,SUP | Y | N | Y | Y | Y | Y | Y,STRETCH | Y | Y | Y | Y,I | PAIN | Y | N | Y | 17 | DIMITRIOS 2012 |
| Y | Y | Y, I | Y,UN | Y | N | Y | Y | Y | Y | Y,BRACE | Y | Y | Y | Y,I | PAIN | Y | N | N | 16 | PETERSEN 2007 |
| Y | Y | Y, I | Y,UN | Y | N | Y | Y | Y | Y | Y,GTN | N | Y | Y | Y,I | PAIN | Y | N | N | 15 | STEUNEBRINK 2013 |
| Y | Y | Y, I | Y,UN | Y | N | Y | Y | Y | Y | Y,ESWT | Y | Y | Y | Y,I | PAIN | Y | N | Y | 17 | ROMPE 2009 |
| Y | Y | Y, I | Y,UN | Y | N | Y | Y | Y | Y | Y,EX ONLY | N | Y | Y | Y,I | PAIN | Y | N | Y | 16 | YOUNG 2005 |
| Y | Y | Y, I | Y,UN | N | N | Y | Y | Y | Y | Y,SPLINT | N | Y | Y | Y,I | PAIN | Y | N | N | 14 | DE JONGE 2010 |
| Y | Y | Y, I | Y,UN | Y | N | Y | Y | Y | Y | Y,SUPP | Y | Y | Y | Y,I | PAIN | Y | N | Y | 17 | PRAET 2019 |
| Y | Y | Y, I | Y,UN | N | N | Y | Y | Y | Y | Y,ORTHOSES | Y | Y | Y | Y,G | N | Y | N | N | 14 | RATHLEFF 2015 |
| Y | YY | Y, I | Y,UN | N | N | N | N | Y | Y | Y,BRACE | N | Y | Y | Y,G | N | Y | N | N | 11 | KNOBLOCH 2008 |
| Y | Y | Y, I | Y,UN | N | N | N | N | N | Y | Y,SPLINT | N | Y | N | Y,G | N | N | N | N | 8 | WHEELER 2017 |
| Y | Y | Y,I | Y,UN | N | N | N | N | Y | Y | Y,SUPP | N | Y | Y | Y,I | PAIN | Y | N | N | 12 | CHOUDHARY 2021 |
| Y | Y | Y,I | Y,UN | Y | N | Y | Y | Y | Y | Y,MHT | Y | Y | Y | Y,I | DIFFICULTY | Y | N | Y | 17 | COWAN 2021 |
| Y | Y | Y,I | Y,UN | Y | N | Y | Y | Y | Y | Y,EX ONLY | N | Y | Y | Y,I | PAIN | Y | N | Y | 16 | HABETS 2021 |
| Y | Y | Y,I | Y,UN | Y | N | Y | Y | Y | Y | Y,EX ONLY | Y | Y | Y | Y,I | PAIN | Y | N | Y | 17 | RUFFINO 2021 |
| Y | Y | Y,I | Y,UN | N | N | Y | Y | Y | Y | Y,IGF-1 | N | Y | Y | Y,I | PAIN | Y | N | N | 14 | OLESEN 2021 |
| Y | Y | Y,I | Y,UN | Y | N | Y | Y | Y | Y | Y,EX ONLY | Y | Y | Y | Y,I | PAIN | Y | Y | Y | 18 | HASANI 2021 |
| Y | Y | Y,I | Y,UN | N | N | N | N | Y | Y | Y,ESWT | Y | Y | Y | Y,G | N | Y | N | N | 12 | MANSUR 2021 |
| Y | Y | Y,I | Y,UN | Y | N | Y | Y | Y | Y | Y,EX ONLY | Y | Y | Y | Y,I | PAIN | Y | Y | Y | 18 | SPRAGUE 2021 |
| Y | Y | Y,I | Y,UN | Y | N | Y | Y | Y | Y | Y,EX ONLY | N | Y | Y | Y,I | PAIN | Y | N | Y | 17 | AGERGAARD 2021 |
| Y | Y | Y,I | Y,UN | N | N | Y | Y | Y | Y | Y,DN,PNE | N | Y | Y | Y,I | PAIN | Y | N | N | 14 | LOPEZ-ROYO 2021 |
| Y | Y | Y,I | Y,UN | N | N | N | N | Y | Y | Y,ESWT | N | Y | Y | Y,G | N | Y | N | N | 11 | ABDELKADER 2021 |
| Y | Y | Y,I | Y,UN | Y | N | Y | Y | Y | Y | Y,HVIGI | Y | Y | Y | Y,I | PAIN | Y | N | Y | 17 | VAN DER VLIST 2020 |
| Y | Y | Y,I | Y,UN | Y | N | Y | Y | Y | Y | Y,EX ONLY | Y | Y | Y | Y,I | PAIN | Y | N | Y | 17 | BREDA 2020 |
| Y | Y | Y,I | Y,UN | Y | N | Y | Y | Y | Y | Y,HEEL LIFTS | Y | Y | Y | Y,I | PAIN | Y | N | Y | 17 | RABUSIN 2021 |
| Y | Y | Y,I | Y,UN | N | N | N | N | Y | Y | Y,DN | Y | N | N | Y,I | PAIN | N | N | Y | 11 | SOLOMONS 2020 |
| Y | Y | Y,I | Y,UN | N | N | N | N | Y | Y | Y,ESWT | Y | Y | Y | Y,G | N | Y | N | N | 12 | RAMON 2020 |
| N | Y | Y,I | Y,UN | N | N | N | N | N | N | Y,PRP | N | N | N | Y,G | N | N | N | N | 5 | SCOTT 2019 |
| Y | Y | Y,I | Y,UN | N | N | Y | Y | Y | Y | Y,PM | N | Y | Y | Y,I | PAIN | Y | N | N | 14 | STEFANSSON 2019 |
| Y | Y | Y,I | Y,UN | Y | N | N | N | Y | Y | Y,PRP,HVIGI | Y | Y | Y | Y,I | PAIN | Y | N | Y | 15 | BOESEN 2017 |
| Y | Y | Y,I | Y,UN | Y | N | N | N | N | N | Y,ORTHOSES | Y | Y | Y | Y,I | PAIN | Y | Y | Y | 14 | CHESTERTON 2021 |
| N | N | N | N | N | N | N | N | N | N | N | N | N | N | N | N | N | N | N | 0 | RASENBERG 2020 |
| N | Y | Y,I | Y,UN | Y | N | N | N | N | N | Y,SURGERY,CSI | Y | N | N | N | N | Y | N | Y | 8 | JOHANNSEN 2020 |
| Y | Y | Y,I | Y,UN | Y | N | Y | Y | Y | Y | Y,EX ONLY | Y | Y | Y | Y,I | DIFFICULTY | Y | N | Y | 17 | THONG-ON 2019 |
| N | Y | Y,I | Y,UN | N | N | Y | Y | Y | Y | Y,MT | N | N | N | Y,G | N | Y | N | N | 10 | CIL 2019 |
| Y | Y | Y,I | Y,UN | N | N | Y | Y | Y | Y | Y,EX | N | Y | Y | Y,G | N | Y | N | N | 13 | KAMONSEKI 2016 |
| N | N | N | N | Y | N | N | N | N | N | N | N | N | N | N | N | N | N | N | 1 | BROWN 2006 |
| Y | Y | Y,I | Y,UN | Y | N | Y | Y | Y | Y | Y,EX | Y | Y | Y | Y,I | PAIN | Y | N | Y | 17 | NIESEN-VERTOMMEN |
| Y | Y | Y,I | Y,BOTH | Y | N | Y | Y | Y | Y | Y,EX ONLY | N | Y | Y | Y,I | DIFFICULTY | Y | N | Y | 16 | JENSEN 1989 |
| Y | Y | Y,I | Y,UN | N | Y | Y | Y | Y | Y | Y,EX ONLY | N | Y | Y | Y,I | PAIN | Y | N | N | 15 | YU 2013 |
| Y | Y | Y,I | Y,UN | N | N | Y | Y | N | Y | Y,ESWT | N | Y | Y | Y,I | PAIN | Y | N | N | 13 | WHEELER 2021 |
| Y | Y | Y,I | Y,UN | N | N | Y | Y | Y | Y | Y,EX ONLY | N | Y | Y | Y,I | PAIN | Y | N | N | 14 | ZHANG 2013 |
| Y | Y | Y,I | Y,UN | Y | N | N | N | N | Y | Y,EX ONLY | Y | Y | Y | Y,I | PAIN | Y | N | Y | 14 | BELL 2013 |
| Y | Y | Y,I | Y,SUP | N | N | N | N | Y | Y | Y,EX ONLY | N | Y | Y | Y,G | N | Y | N | Y | 12 | PIETROSIMONE |
| Y | Y | Y,I | Y,UN | Y | N | N | N | N | Y | Y,PRP | N | N | Y | Y,I | PAIN | Y | N | N | 11 | DE JONGE 2011 |
| Y | Y | Y,I | Y,UN | Y | N | N | N | N | Y | Y,PRP | N | N | Y | Y,I | PAIN | Y | N | N | 11 | DE VOS 2010 |
| Y | Y | Y,I | Y,UN | Y | N | Y | Y | Y | Y | Y,US | Y | Y | Y | Y,I | PAIN | Y | N | Y | 17 | WARDEN 2008 |
| Y | Y | Y,I | Y,UN | Y | N | Y | Y | Y | Y | Y,EX | N | Y | Y | Y,I | PAIN | Y | N | N | 15 | VISNES 2005 |
| Y | Y | Y,I | Y,UN | N | N | Y | Y | Y | Y | Y,EX ONLY | N | Y | Y | Y,I | PAIN | Y | N | N | 14 | VAN ARK 2018 |
| N | Y | Y,I | Y,UN | N | N | N | Y | N | Y | Y,PRP | N | Y | Y | Y,I | PAIN | N | N | N | 10 | THOMPSON 2019 |
| Y | N | Y,I | Y,UN | N | N | N | N | Y | N | Y,CON RX | N | N | Y | N | N | Y | N | N | 7 | CACCHIO 2011 |
| Y | Y | Y,I | Y,UN | Y | N | Y | Y | Y | Y | Y,ORTHOSES | Y | Y | Y | Y,I | PAIN | Y | N | N | 16 | MUNTEANU 2014 |
| Y | Y | Y,I | Y,UN | Y | N | Y | Y | Y | Y | Y,ESWT | Y | Y | Y | Y,I | PAIN | Y | N | N | 16 | VAN DER WORP 2014 |
| Y | Y | Y,I | Y,UN | Y | N | Y | Y | Y | Y | Y, VIB/CRY | N | Y | Y | Y,I | PAIN | Y | N | N | 15 | ROMER-MORALES 2018 |
| Y | Y | Y,I | Y,UN | Y | N | Y | Y | Y | Y | Y,VIB/CRY | N | Y | Y | Y,I | PAIN | Y | N | N | 15 | ROMERO-MORALES 2020 |
| Y | Y | Y,I | Y,UN | Y | N | N | N | N | Y | Y,EX ONLY | N | Y | Y | Y,G | N | Y | N | N | 11 | RYAN 2014 |
| Y | Y | Y,I | Y,SUP | N | N | Y | Y | Y | Y | Y,EX ONLY | N | Y | Y | Y,G | RESISTANCE | Y | N | N | 14 | RIEL 2018 |
| Y | Y | Y,I | Y,SUP | N | N | N | N | Y | N | Y,MT | N | Y | Y | Y,G | N | Y | N | N | 10 | KOSZALINSKI 2020 |
| N | Y | Y,I | N | N | N | N | N | N | N | Y,ABI | N | N | N | Y,G | PAIN | N | N | N | 5 | PEARSON 2012 |
| N | N | Y,I | N | N | N | N | N | N | N | Y,ESWT | N | N | N | Y,G | N | N | N | N | 3 | WANG 2007 |
| N | N | Y,I | N | N | N | N | N | N | N | Y,CHELT | N | N | N | Y,G | N | N | N | N | 3 | NOTARNICOLA 2013 |
| N | Y | Y,I | N | N | N | N | N | N | N | Y,PRP | Y | N | N | Y,G | N | N | N | N | 5 | DRAGOO 2014 |
| Y | Y | Y,I | Y,SUP | N | N | Y | Y | Y | Y | Y,PRP,HAI | N | Y | Y | Y,G | Y | N | N | N | 13 | KAUX 2019 |
| Y | Y | Y,I | N | N | N | N | N | Y | N | Y,USGET | N | N | Y | Y,G | N | Y | N | N | 8 | ABAT 2016 |
| Y | Y | Y,I | Y,UN | N | N | Y | Y | Y | Y | Y,EX ONLY | N | Y | Y | Y,I | PAIN | Y | N | N | 14 | BIERNAT 2014 |
| Y | Y | Y,I | Y,SUP | Y | N | N | N | Y | Y | Y,EX ONLY | N | Y | Y | Y,G | N | Y | N | Y | 13 | RIO 2015 |
| Y | Y | Y,I | Y,SUP | Y | N | N | N | Y | Y | Y,EX ONLY | N | Y | Y | Y,G | N | Y | N | Y | 13 | HOLDEN 2020 |
| Y | Y | Y,I | Y,SUP | Y | N | Y | Y | Y | Y | Y,EX ONLY | N | Y | Y | Y,I | FATIGUE | Y | N | Y | 16 | RIO 2017 |
| Y | Y | Y,I | Y,UN | Y | N | Y | Y | Y | Y | Y,EDUCATION | Y | Y | Y | Y,I | PAIN | Y | Y | Y | 18 | SANCHO 2019 |
| Y | Y | Y,I | Y,SUP | Y | N | Y | Y | Y | Y | Y,CON RX | N | Y | Y | Y,I | PAIN | Y | N | Y | 16 | CROISIER 2001 |
| Y | Y | Y,I | Y,UN | N | N | Y | Y | Y | Y | Y,EX ONLY | N | Y | Y | Y,I | PAIN | Y | N | N | 14 | OHBERG 2004 |
| Y | Y | Y,I | Y,UN | Y | N | Y | Y | Y | Y | Y,EX ONLY | N | Y | Y | Y,I | PAIN | Y | N | Y | 16 | SAYANA 2007 |
| Y | Y | Y,I | Y,SUP | Y | N | N | N | Y | N | Y, EPI | Y | Y | Y | Y,G | N | Y | N | N | 12 | ABAT 2014 |
| Y | Y | Y,I | Y,UN | Y | N | Y | Y | Y | Y | Y,CSI | Y | Y | Y | Y,I | RESISTANCE | Y | Y | Y | 18 | RIEL 2019 |
| Y | Y | Y,I | Y,SUP | Y | N | Y | Y | Y | Y | Y,EX ONLY | N | Y | Y | Y,I | PAIN | Y | N | N | 15 | KONGSGAARD 2010 |
| Y | Y | Y,I | Y,UN | Y | N | Y | Y | Y | Y | Y,CSI | N | Y | Y | Y,I | PAIN | Y | N | Y | 16 | WETKE 2015 |
| Y | Y | Y,I | Y,UN | Y | N | Y | Y | Y | Y | Y,EX ONLY | N | Y | Y | Y,I | PAIN | Y | N | Y | 16 | MAFFULLI 2008 |
| Y | Y | Y,I | Y,UN | Y | N | Y | Y | Y | Y | Y,EX ONLY | N | Y | Y | Y,I | PAIN | Y | N | Y | 16 | SHALABI 2004 |
| Y | Y | Y,I | Y,UN | N | N | Y | Y | Y | Y | Y,ESWT | N | Y | Y | Y,I | PAIN | Y | N | N | 14 | MANSUR 2019 |
| Y | Y | Y,I | Y,SUP | N | N | N | N | Y | N | Y, EPI | N | Y | Y | Y,G | N | Y | N | N | 10 | ABAT 2015 |
| Y | Y | Y,I | Y,UN | N | N | Y | Y | Y | Y | Y,EX ONLY | N | Y | Y | Y,I | PAIN | Y | N | N | 14 | ALFREDSON 1999 |
| Y | Y | Y,I | Y,UN | N | N | Y | Y | Y | Y | Y,EX ONLY | N | Y | Y | Y,I | PAIN | Y | N | N | 14 | ALFREDSON 2003 |
| Y | Y | Y,I | Y,SUP | N | N | N | N | Y | Y | Y,EX ONLY | N | Y | Y | Y,G | N | Y | N | N | 11 | O'NEILL 2019 |
| N | N | Y,I | N | N | N | N | N | N | N | N | N | N | Y | N | N | N | N | N | 2 | OOI 2019 |
| N | Y | Y,I | Y,SUP | N | N | N | N | N | N | Y,PRP | N | N | N | N | N | N | N | N | 4 | KAUX 2015 |
| N | N | Y,I | N | N | N | N | N | N | N | Y,PRP | N | N | N | N | N | N | N | N | 2 | DE JONGE 2015 |
| N | N | Y,I | N | N | N | N | N | N | N | N | N | N | N | Y,G | N | N | N | N | 2 | PANNI 2000 |
| Y | Y | Y,I | Y,UN | Y | N | Y | Y | Y | Y | Y,CON RX | N | Y | Y | Y,I | PAIN | Y | N | N | 15 | ANGERMANN 1999 |
| Y | Y | Y,I | Y,UN | N | N | N | N | Y | Y | Y,EX ONLY | N | Y | Y | Y,G | N | Y | N | N | 11 | VON WEHREN 2019 |
| Y | Y | Y,I | Y,UN | Y | N | Y | Y | Y | Y | Y,PRP | N | Y | Y | Y,I | PAIN | Y | N | Y | 16 | KAUX 2014 |
| Y | Y | Y,I | Y,UN | Y | N | Y | Y | Y | Y | Y,EX ONLY | N | Y | Y | Y,I | PAIN | Y | N | N | 15 | WEI 2017 |
| Y | Y | Y,I | Y,SUP | N | N | Y | Y | Y | Y | Y,ES | N | Y | Y | Y,I | PAIN | Y | N | N | 14 | BASAS 2018 |
| Y | Y | Y,I | Y,UN | N | N | Y | Y | Y | Y | Y,EX ONLY | N | Y | Y | Y,I | PAIN | Y | N | N | 14 | FAHLSTROM 2007 |
| Y | Y | Y,I | Y,UN | N | N | Y | Y | Y | Y | Y,EX | N | Y | Y | Y,I | PAIN | Y | N | N | 14 | JONSSON 2008 |
| Y | Y | Y,I | Y,UN | Y | N | Y | Y | Y | Y | Y,PRP | Y | Y | Y | Y,I | PAIN | Y | N | N | 16 | ABATE 2020 |
| N | N | Y,I | Y,UN | N | N | N | N | N | N | Y,ESWT,HVIGI | N | N | N | N | N | N | N | N | 3 | WHEELER 2020 |
| N | N | N | N | N | N | N | N | N | N | N | N | N | N | N | N | N | N | N | 0 | LAGAS 2021 |
| Y | Y | Y,I | Y,UN | N | N | N | N | Y | Y | Y,ESWT | Y | Y | Y | Y,G | N | Y | N | N | 12 | ROBINSON 2021 |
| N | Y | Y,I | N | N | N | N | N | Y | N | Y,ESWT | N | N | N | Y,G | N | N | N | N | 5 | WHEELER 2021 |
| Y | Y | Y,I | Y,SUP | Y | Y | Y | Y | Y | Y | Y,EX ONLY | Y | Y | Y | Y,I | PAIN, RPE | Y | N | Y | 18 | MANTOVANI 2020 |
| Y | Y | Y,I | Y,UN | Y | N | Y | Y | Y | Y | Y,EX ONLY | N | Y | Y | Y,I | RESISTANCE | Y | N | N | 15 | KULIG 2009 |
| Y | N | Y,I | N | N | N | N | N | N | N | Y,ACP | N | N | N | Y,G | N | N | N | N | 4 | DEANS 2012 |
| Y | N | Y,I | N | N | N | N | N | N | N | Y,ESWT | N | N | N | Y,G | N | N | N | N | 4 | PAVONE 2016 |
| Y | Y | Y,I | Y,SUP | N | Y | Y | Y | Y | Y | Y,EX ONLY | N | Y | Y | Y,I | RESISTANCE | Y | N | N | 15 | ROMERO-RODRIGUEZ 2011 |
| N | Y | Y,I | Y,UN | N | N | N | N | N | N | Y,ESWT | N | N | N | Y,I | N | N | N | N | 5 | WHEELER 2019 |
| Y | Y | Y,I | Y,UN | N | N | N | N | Y | Y | Y,MT | N | Y | Y | Y,I | N | Y | N | N | 11 | SYVERSTON 2017 |
| Y | Y | Y,I | Y,UN | N | N | N | N | Y | Y | Y,ESWT | N | Y | Y | Y,I | N | Y | N | N | 11 | ROBINSON 2020 |
| Y | Y | Y,I | Y,UN | N | N | Y | Y | Y | Y | Y,MT | N | Y | Y | Y,I | N | Y | N | N | 13 | BENITO 2016 |
| Y | Y | Y,I | Y,UN | N | N | Y | Y | Y | Y | Y,EX ONLY | N | Y | Y | Y,I | PAIN | Y | N | N | 14 | SILBERNAGEL 2011 |
| N | Y | Y,I | Y,UN | N | N | N | N | N | N | Y,HVIGI | N | N | N | Y,I | N | N | N | N | 5 | MORTON 2014 |
| Y | Y | Y,I | Y,UN | Y | N | Y | Y | Y | Y | Y,EX ONLY | N | Y | Y | Y,I | PAIN | Y | N | Y | 16 | MASOOD 2014 |
| Y | Y | Y,I | Y,UN | Y | N | Y | Y | Y | Y | Y,EX ONLY | N | Y | Y | Y,I | PAIN | Y | N | Y | 16 | VAN ARK 2013 |
| Y | Y | Y,I | Y,UN | N | N | N | N | Y | Y | Y,UGPE | N | Y | Y | Y,I | PAIN | Y | N | N | 12 | MUNOZ FERNANDEZ 2021 |
| Y | Y | Y,I | Y,SUP | Y | N | Y | Y | Y | Y | Y,EX ONLY | Y | Y | Y | Y,I | PAIN | Y | N | Y | 17 | SKOVLUND 2020 |
| Y | Y | Y,I | Y,UN | Y | N | N | N | Y | Y | Y,MT | N | Y | Y | Y,G | N | Y | N | N | 12 | JAYASEELAN 2017 |
| Y | Y | Y,I | Y,SUP | N | N | Y | Y | Y | Y | Y,MT | N | Y | Y | Y,I | PAIN | Y | N | N | 14 | BIANCO 2019 |
| Y | Y | Y,I | Y,UN | N | N | Y | Y | Y | Y | Y,E-STIM | N | Y | Y | Y,I | PAIN | Y | N | N | 14 | ECKENRODE 2015 |
| Y | Y | Y,I | Y,UN | N | N | Y | Y | Y | Y | Y,MT | N | Y | Y | Y,G | N | Y | N | N | 13 | PAPA 2012 |
| Y | Y | Y,I | Y,UN | N | N | N | N | N | N | Y,MT | N | Y | Y | Y,G | N | Y | N | N | 9 | DOSS ANTOS 2016 |
| N | N | Y,I | Y,BOTH | N | N | N | N | N | N | Y,MT | N | N | N | Y,G | N | N | N | N | 4 | LEE 2019 |
| Y | Y | Y,I | Y,UN | Y | N | Y | Y | Y | Y | Y,MT | N | Y | Y | Y,I | PAIN | Y | N | Y | 16 | ROSS 2017 |
| Y | Y | Y,I | Y,SUP | N | N | Y | Y | Y | Y | Y,EX ONLY | N | Y | Y | Y,I | PAIN | Y | N | Y | 15 | CUDDEFORD 2020 |
| Y | Y | Y,I | Y,UN | Y | N | Y | Y | Y | Y | Y,EX ONLY | N | Y | Y | Y,I | PAIN,RPE | Y | N | N | 15 | KRUEGER 2020 |
| Y | Y | Y,I | Y,UN | N | N | N | N | Y | Y | Y,MT | N | Y | Y | Y,G | N | Y | N | N | 11 | BORDA 2017 |
| Y | Y | Y,I | Y,UN | Y | N | Y | Y | Y | Y | Y,EX ONLY | N | Y | Y | Y,I | PAIN | Y | N | N | 15 | RAUSEO 2017 |
| Y | Y | Y,I | Y,UN | N | N | Y | Y | Y | Y | Y,MT | N | Y | Y | Y,I | PAIN | Y | N | N | 14 | MCCORMACK 2012 |
| Y | Y | Y,I | Y,UN | N | N | Y | Y | Y | Y | Y,MT | N | Y | Y | Y,G | N | Y | N | N | 13 | PATLA 2015 |
| Y | Y | Y,I | Y,SUP | N | N | Y | Y | Y | Y | Y,MT | N | Y | Y | Y,I | PAIN | Y | N | N | 14 | PINKELMAN 2012 |
| Y | Y | Y,I | Y,UN | Y | N | Y | Y | Y | Y | Y,ESWT | N | Y | Y | Y,G | N | Y | N | Y | 15 | FRANCIS 2020 |
| Y | Y | Y,I | Y,UN | N | N | Y | Y | Y | Y | Y,EX ONLY | N | Y | Y | Y,G | N | Y | N | N | 13 | MCCREESH 2013 |
| Y | Y | Y,I | Y,UN | N | N | Y | Y | Y | Y | Y,MT | N | Y | Y | Y,G | N | Y | N | N | 13 | HENSLEY 2012 |
| Y | Y | Y,I | Y,SUP | N | N | N | N | Y | Y | Y,EX ONLY | N | Y | Y | Y,I | PAIN | Y | N | N | 12 | CUSHMAN 2015 |
| Y | Y | Y,I | Y,UN | N | N | Y | Y | Y | Y | Y,EX ONLY | N | Y | Y | Y,I | LOAD | Y | N | N | 14 | THOMPSON 2017 |
| Y | Y | Y,I | Y,UN | N | N | Y | Y | Y | Y | Y,MT | N | Y | Y | Y,I | PAIN, DIFFICULTLY | Y | N | N | 14 | JAYASEELAN 2014 |
| Y | Y | Y,I | Y,UN | Y | N | Y | Y | Y | Y | Y,EX ONLY | N | Y | Y | Y,I | PAIN | Y | N | Y | 16 | DUMONT 2006 |
| Y | Y | Y,I | Y,SUP | N | N | Y | Y | Y | Y | Y,EX ONLY | N | Y | Y | Y,I | PAIN | Y | N | N | 14 | SILVA 2015 |
| Y | Y | Y,I | Y,SUP | N | N | N | N | Y | Y | Y,MT | Y | Y | Y | Y,I | DIFFICULTLY | Y | N | N | 13 | MCCORMACK 2012 |
| Y | Y | Y,I | Y,UN | N | N | N | N | Y | Y | Y,MT | N | Y | Y | Y,I | PAIN | Y | N | N | 12 | VAN ROOY 2009 |
| Y | Y | Y,I | Y,BOTH | Y | N | Y | Y | Y | Y | Y,EX ONLY | N | Y | Y | Y,G | N | Y | N | N | 14 | GREENE 2002 |
| Y | Y | Y,I | Y,UN | N | N | N | N | Y | N | Y,PRP | N | Y | Y | Y,G | N | Y | N | N | 10 | ROWAN 2013 |
| Y | Y | Y,I | Y,BOTH | N | N | Y | Y | Y | Y | Y,EX ONLY | N | Y | Y | Y,I | PAIN | Y | N | N | 14 | GOLDMAN 2010 |
| Y | Y | Y,I | Y,SUP | N | N | Y | Y | Y | Y | Y,EX ONLY | N | Y | Y | Y,I | DIFFICULTLY | Y | N | Y | 15 | CUDDEFORD 2018 |
| Y | N | Y,I | Y,UN | N | N | N | N | Y | Y | Y,EX ONLY | N | Y | Y | Y,G | N | Y | N | N | 10 | GARDIN 2010 |
| Y | Y | Y,I | Y,UN | N | N | Y | Y | Y | Y | Y,EX ONLY | N | Y | Y | Y,I | PAIN | Y | N | Y | 15 | LANGBERG 2007 |
| Y | Y | Y,I | Y,UN | Y | N | Y | Y | Y | Y | Y,EX ONLY | Y | Y | Y | Y,I | PAIN | Y | N | Y | 17 | STASINOPOULOS 2013 |
| Y | Y | Y,I | Y,UN | N | N | N | N | Y | Y | Y,EX ONLY | N | Y | Y | Y,I | N | Y | N | N | 11 | KANNIAPPAN 2020 |
| Y | Y | Y,I | Y,UN | N | N | Y | Y | Y | Y | Y,EX ONLY | N | Y | Y | Y,I | PAIN | Y | N | N | 14 | PURDAM 2004 |
| Y | Y | Y,I | Y,SUP | Y | Y | Y | Y | Y | Y | Y,EX ONLY | N | Y | Y | Y,I | PAIN,RPE | Y | N | Y | 17 | VAN DER VLIST 2020 |
| N | Y | Y,I | Y,UN | N | N | N | N | N | N | Y,EX ONLY | N | N | Y | Y,G | N | N | N | N | 6 | MORGAN 2018 |
| Y | Y | Y,I | Y,UN | N | N | Y | Y | Y | Y | Y,EX ONLY | N | Y | Y | Y,I | PAIN | Y | N | N | 14 | DE VOS 2012 |
| Y | Y | Y,I | Y,UN | N | N | Y | Y | Y | Y | Y,EX ONLY | N | Y | Y | Y,I | PAIN | Y | N | N | 14 | PARK 2021 |
| Y | Y | Y,I | Y,UN | Y | N | N | N | N | Y | Y,EX ONLY | N | Y | Y | Y,I | PAIN | Y | N | Y | 13 | RAM 2013 |
| Y | Y | Y,I | Y,BOTH | N | N | Y | Y | Y | Y | Y,EX ONLY | Y | Y | Y | Y,G | N | Y | N | N | 14 | VANDER DOELEN 2020 |

**Abbreviations:** Y: yes, N: no, UN: unsupervised,, SUP: supervised, G: general, I: individualised, CERT: consensus on exercise reporting template, EX: exercise, RPE: rating of perceived exertion, MT: manual therapy, ESWT: extracorporeal shockwave therapy, PRP: platelet-rich plasma, UGPE: ultrasound guided percutaneous electrolysis, LLLT: low-level laser therapy, ESTIM: electrical stimulation, CON RX: conventional rehabilitation, CSI: corticosteroid injection, ABI: autologous blood injection: ACP: autologous conditioned plasma
